# Statistical distributions of test statistics used for quantitative trait association mapping in structured populations

**DOI:** 10.1186/1297-9686-44-32

**Published:** 2012-11-12

**Authors:** Simon Teyssèdre, Jean-Michel Elsen, Anne Ricard

**Affiliations:** 1 INRA, UR 631 Station d’Amélioration Génétique des Animaux, Castanet-Tolosan, F-31326, France; 2 INRA, UMR 1313 Génétique Animale et Biologie Intégrative, Jouy-en-Josas F-78352, France

## Abstract

**Background:**

Spurious associations between single nucleotide polymorphisms and phenotypes are a major issue in genome-wide association studies and have led to underestimation of type 1 error rate and overestimation of the number of quantitative trait loci found. Many authors have investigated the influence of population structure on the robustness of methods by simulation. This paper is aimed at developing further the algebraic formalization of power and type 1 error rate for some of the classical statistical methods used: simple regression, two approximate methods of mixed models involving the effect of a single nucleotide polymorphism (SNP) and a random polygenic effect (GRAMMAR and FASTA) and the transmission/disequilibrium test for quantitative traits and nuclear families. Analytical formulae were derived using matrix algebra for the first and second moments of the statistical tests, assuming a true mixed model with a polygenic effect and SNP effects.

**Results:**

The expectation and variance of the test statistics and their marginal expectations and variances according to the distribution of genotypes and estimators of variance components are given as a function of the relationship matrix and of the heritability of the polygenic effect. These formulae were used to compute type 1 error rate and power for any kind of relationship matrix between phenotyped and genotyped individuals for any level of heritability. For the regression method, type 1 error rate increased with the variability of relationships and with heritability, but decreased with the GRAMMAR method and was not affected with the FASTA and quantitative transmission/disequilibrium test methods.

**Conclusions:**

The formulae can be easily used to provide the correct threshold of type 1 error rate and to calculate the power when designing experiments or data collection protocols. The results concerning the efficacy of each method agree with simulation results in the literature but were generalized in this work. The power of the GRAMMAR method was equal to the power of the FASTA method at the same type 1 error rate. The power of the quantitative transmission/disequilibrium test was low. In conclusion, the FASTA method, which is very close to the full mixed model, is recommended in association mapping studies.

## Background

Single Nucleotide Polymorphism (SNP) information has enabled the use of linkage disequilibrium to detect and localize loci affecting phenotypes. The first methods developed searched for disequilibrium between one or a few marker loci and loci responsible for disease susceptibility. Case–control designs were used [1]. Typically, data were analyzed to compare the frequency of marker alleles between healthy and diseased individuals, for instance using the relative risk criterion [2]. A similar approach for quantitative traits (including production traits in animals or plants) was to model the expectation of their distribution as a linear combination of marker genotype, allele or haplotype effects. Grapes et al. [3] and Zhao et al. [4] demonstrated that the single marker regression model is as powerful and precise as other more sophisticated techniques, such as multiple regression, regression on haplotypes, or the IBD method proposed by Meuwissen and Goddard [5].

Detection of spurious associations is a major issue that has been investigated by many authors. Such errors occur when population classification based on marker information is confounded with another source of heterogeneity that affects the trait being analyzed. The problem of genetic heterogeneity has been the most widely studied. Two non-exclusive situations can occur: (i) the population consists of genetically different subpopulations and (ii) the population consists of related individuals, which may be recorded through pedigree or not. Several studies have clearly shown that neither relative risk nor simple regression is robust to genetic stratification of the population resulting from the mixture of different groups (breeds, lines, etc.) or families [6-9].

Many approaches have been proposed to avoid the effects of spurious associations. The first was to restrict the analysis to within-family comparisons, linking association analysis to transmission studies. Within this framework, samples have to be carefully organized and *ad hoc* families have to be recruited. They are based on the association, within heterozygous parents family, of segregation distortion at a marker locus and progeny phenotypes. This idea was first implemented in the transmission disequilibrium test (tdt) designed by Spielman et al. [10] and then further developed by others. Ewans and Spielman [11], when comparing tdt and a “within-family contingency statistic” that is similar to the haplotype relative risk developed by Falk and Rubinstein [12], demonstrated the robustness of tdt in various subdivision and admixture scenarios.

Two widely represented families of methods extend these within-family comparisons to quantitative traits: the “quantitative tdt” or QTDT by Abecassis et al. ([13-16]) and the family-based association tests or fbat [17-20]. All these methods are robust to population stratifications, have similar power [21,22], and are more powerful than the first tests developed for family-based association studies [14].

Although limiting spurious associations by using within-family analyses was very successful, case–control association studies in populations consisting of individuals assumed to be unrelated were nevertheless frequently performed, in particular because the recruitment of the corresponding samples is much easier [23]. A number of techniques were derived to limit false positives: “genomic control” corrects the test statistic [24,25], a structure effect can be added to the model of analysis [26-31], and marker transmission used in family-based tests can be generalized and used between generations [5,32].

Concerning quantitative traits, known or hidden population structures can be modeled in mixed models where the phenotype expectation is modeled as the sum of fixed effects, including the effect of the genetic marker being tested, and a random individual polygenic effect. Covariances between the individual polygenic effects are proportional to the polygenic variance and coancestry coefficients, which can be estimated from pedigree or marker information [33-36]. This mixed model is a standard that has been used in animal breeding and genetics for many years [37,38] and more recently in human genetics [39,40].

In these mixed models, polygenic and residual variances have to be estimated separately for each marker fitted before its significance is tested. This estimation phase, to be repeated for each marker tested, can be a limiting factor in large designs and simpler approaches have been proposed. The GRAMMAR method was developed by Aulchenko et al. [41,42] and by Amin et al. [43] to test marker effects on phenotypes that have been corrected for an estimate of the individual’s polygenic effect in a restricted model that is free of the polygenic effect. The FASTA approach described by Chen and Abecasis [44] is a score test, derived from the generalized FBAT [18]. In a first step, environmental fixed effects and polygenic and residual variances are estimated from a mixed model excluding the marker effect. Then, corrected phenotypes are successively correlated to each marker’s genotypes using these estimations, giving FBAT type scores. A similar approach can be considered in which the second step would be based on a simple fixed effect model as in GRAMMAR.

Other approaches have been proposed, with the aim of accelerating computations (emma for efficient mixed-model association, [45], emmax for eXpedited, [46] and P3D for Population Parameters Previously Determined, [40]). Finally, a few models deal with spurious associations arising from subpopulations and family structures [39,43,47-49].

The above methods have been evaluated by simulations. Aulchenko et al. [41] compared GRAMMAR to the full mixed model, to the regression model without a polygenic effect, to the QTDT method, and to a simple fbat by using simulated datasets that corresponded to typical pedigrees. Genomic control was compared in [43] using GRAMMAR and GRAMMAR-GC. Price et al. [39] compared Pca (eigenstrat), Armitage test, emmax with or without pca and roadtrips proposed by Thornton and McPeek [50], in which genomic data are modeled as random variables. Pca-based approaches ([26], eigenstrat; [51], pca-based logistic regression; [52], lapstruct (which makes use of spectral graph theory to build principal components) were compared in [53] to the genomic control described by Devlin and Roeder [24] and to roadtrips. Three GWAS (genome-wide association studies) techniques were compared in [54]: simple regression, GRAMMAR and a “mtdt”, which is a QTDT applied to Mendelian sampling terms.

On the whole, these numerical studies have shown that within-family approaches are less powerful than case control analyses in populations of unrelated individuals [41,48] and that there are no major differences between the latter [3]. These studies have clearly demonstrated the non-robustness of the simplest methods such as the Armitage test or simple regression [47,53-55] and that more elaborate models are robust to any type of stratification [39,47,49]. Furthermore, these studies have shown that approximate techniques such as GRAMMAR and emmax are very efficient in terms of error control when family structures exist, as well as in computing speed, but are less powerful in certain situations e.g. [41,46].

One of the main limits of comparing methods based on simulations is that the simulation results cannot be generalized and only a few studies have provided algebraic results but for simple situations. For instance, Fan and Xiong [56] formalized single- or bi-marker association analyses by regression, deriving their power as a function of the non-centrality parameter of the test statistic, which depends on the linkage disequilibrium (LD) between the markers and the quantitative trait locus (QTL). In [11], the relative risk, the within-family contingency statistic and the tdt were compared algebraically using a few admixture scenarios. The Cochran Armitage test was studied by different authors [57-59]. The power of ANOVA or regression-based association analyses was derived by Ambrosius et al. [60] as a function of allelic or genotypic frequencies, and recently completed by Kozlitina et al. [61]. Abecacis et al. [13] obtained results for the QTDT in population mixture situations, by deriving within- and between-family expectations with and without parental information. Boitard et al. [62] generalized the corresponding formulae for variances and tests. In [21], Lange et al. provided algebraic formulae representing the power of fbat, depending on parental and progeny genotypes.

The aim of the work presented here was to further develop the algebraic formulation of power and type 1 error rate for four of the aforementioned methods: simple regression, the approximate methods GRAMMAR [41,43] and FASTA [44], and the QTDT described by [13]. Our goal was to explore the effect of population structure but focusing on hidden familial relationships rather than on population mixtures. In such situations, phenotypes are both under the influence of the QTL that is linked to tested markers and the polygenic background. The model of reference used in this study was the standard mixed model which includes the coancestry coefficients as parameters. Results show in which situations the methods studied here can be considered as appropriate and provides some guidance for population sampling.

## Methods

### Statistical concepts

The statistics compared in this paper are testing whether, or not, the variability of a quantitative trait, *y*, is associated with the genotype at a SNP considered one by one. Trait *y* is assumed to be polygenic, i.e. under the influence of many QTL. When testing a particular SNP-phenotype association, the random variable *y* can be described as the sum of the putative fixed effect *β* of a QTL linked to this SNP, a random polygenic effect *u* that represents the collective effect of all other (unlinked) QTL, and random noise *e* (**y** = **1***μ* + **x***β* + **u** + **e**). Hereafter, this model is designated as the “true model”. The approximate methods, mentioned in the introduction, estimate *β* using simplified models. Generally, for each of these simplified models *(i)*, the regression coefficient of the SNP effect (fitted as a covariate according to the number of reference alleles in the genotype, i.e. 0, 1 or 2) is estimated by the general least squares estimator ${\hat{\beta }}^{\left(i\right)}$. A standard Student’s test is then constructed to test the null hypothesis that the SNP effect is zero. Let $E^{\left(i\right)}\left({\hat{\beta }}^{\left(i\right)}\right)$ and $V^{\left(i\right)}\left({\hat{\beta }}^{\left(i\right)}\right)$ be the expectation and variance of the estimator ${\hat{\beta }}^{\left(i\right)}$, and ${\hat{\sigma }}_{E^{\left(i\right)}}^{2}$ and $E^{\left(i\right)}\left({\hat{\sigma }}_{E^{\left(i\right)}}^{2}\right)$ be an estimator of the residual variance and its expectation, all assuming model *(i)*. The t-tests can then be formulated as:

$$\tau^{\left(i\right)}=\frac{{\hat{\beta }}^{\left(i\right)}/\sqrt{V^{\left(i\right)}\left({\hat{\beta }}^{\left(i\right)}\right)}}{\sqrt{{\hat{\sigma }}_{e^{\left(i\right)}}^{2}/E^{\left(i\right)}\left({\hat{\sigma }}_{e^{\prime i)}}^{2}\right)}}$$

As the ratio of a normal distribution with unit variance and an independent square root *χ*^2^ distribution, these tests are assumed to follow non-central t-distributions with non-centrality parameter $E^{\left(i\right)}\left({\hat{\beta }}^{\left(i\right)}\right)/\sqrt{V^{\left(i\right)}\left({\hat{\beta }}^{\left(i\right)}\right)}$. However, these tests do not follow these distributions because **y** does not follow the simplified model *(i)*; only if the tests are computed with expectations and variance of ${\hat{\beta }}^{\left(i\right)}$ corresponding to the true model for **y**, do the tests follow a t-distribution. Let $E\left({\hat{\beta }}^{\left(i\right)}\right)$ and $V\left({\hat{\beta }}^{\left(i\right)}\right)$ be the expectation and variance of the estimator ${\hat{\beta }}^{\left(i\right)}$ and $E\left({\hat{\sigma }}_{E^{\left(i\right)}}^{2}\right)$ the expectation of the estimator of residual variance assuming **y** follows the true model. Then, the valid Student’s tests are:

$$t^{\left(i\right)}=\frac{{\hat{\beta }}^{\left(i\right)}/\sqrt{V\left({\hat{\beta }}^{\left(i\right)}\right)}}{\sqrt{{\hat{\sigma }}_{e^{\left(i\right)}}^{2}/E\left({\hat{\sigma }}_{e^{\left(i\right)}}^{2}\right)}}\text{.}$$

These Student’s distributions tend to normal distributions when the number of animals involved in the analysis is sufficiently high (100 animals). These normal distributions have mean $\frac{E\left({\hat{\beta }}^{\left(i\right)}\right)}{\sqrt{V\left({\hat{\beta }}^{\left(i\right)}\right)}}$ and variance 1 [63]. The test *τ*^(*i*)^ that is used instead of *t*^(*i*)^ can be expressed as $\tau^{\left(i\right)}=t^{\left(i\right)}\sqrt{\frac{V\left({\hat{\beta }}^{\left(i\right)}\right)}{V^{\left(i\right)}\left({\hat{\beta }}^{\left(i\right)}\right)}}\sqrt{\frac{E^{\left(i\right)}\left({\hat{\sigma }}_{E^{\left(i\right)}}^{2}\right)}{E\left({\hat{\sigma }}_{E^{\left(i\right)}}^{2}\right)}}$. Thus, the test *τ*^(*i*)^ will have a normal distribution with mean:

$$E\left(\tau^{\left(i\right)}\right)=E\left({\hat{\beta }}^{\left(i\right)}\right)\sqrt{\frac{1}{V^{\left(i\right)}\left({\hat{\beta }}^{\left(i\right)}\right)}}\sqrt{\frac{E^{\left(i\right)}\left({\hat{\sigma }}_{e^{\left(i\right)}}^{2}\right)}{E\left({\hat{\sigma }}_{e^{\left(i\right)}}^{2}\right)}}$$

and variance:

$$V\left(\tau^{\left(i\right)}\right)=\frac{V\left({\hat{\beta }}^{\left(i\right)}\right)}{V^{\left(i\right)}\left({\hat{\beta }}^{\left(i\right)}\right)}\frac{E^{\left(i\right)}\left({\hat{\sigma }}_{e^{\left(i\right)}}^{2}\right)}{E\left({\hat{\sigma }}_{e^{\left(i\right)}}^{2}\right)}\text{.}$$

The aim of the present study was to express these moments as a function of the parameters of the true model for **y,** i.e. the matrix of relationships among individuals and the polygenic variance. The true type 1 error rate and power of the tests of model *(i)* were analytically determined. Under the null hypothesis (H0, *β* = 0), the tests *τ*^(*i*)^ were assumed to have expectation 0 and variance 1. For a given expected type 1 error rate *α*, the threshold for rejecting the null hypothesis was set at *t*_*α*/2_ = *Φ*^− 1^(1 − *α*/2), where *Φ* is the standardized cumulative normal distribution. With the same threshold, knowledge of the true variance and expectation of the tests *τ*^(*i*)^ allowed us to compute the actual true type 1 error rate $\alpha^{\left(i\right)}=2\left[1-\Phi \left(\frac{t_{\alpha /2}-E_{\beta =0}\left(\tau^{\left(i\right)}\right)}{\sqrt{V_{\beta =0}\left(\tau^{\left(i\right)}\right)}}\right)\right]$, where *E*_*β* = 0_(*τ*^(*i*)^) is the expectation of the test statistic and *V*_*β* = 0_(*τ*^(*i*)^) the variance of the test statistic under the null hypothesis. Under the alternative hypothesis (H1, *β* = *b*), the statistical power was computed as $P_{\alpha ,b}^{\left(i\right)}=1-\Phi \left(\frac{t_{\alpha /2}-E_{\beta =b}\left(\tau^{\left(i\right)}\right)}{\sqrt{V_{\beta =b}\left(\tau^{\left(i\right)}\right)}})\right)$, using the same definition for the threshold and the true regression coefficient *b*. The bias of the estimator of the regression coefficient of the SNP effect was computed as $\left(E_{\beta =b}\left({\hat{\beta }}^{\left(i\right)}\right)-b\right)/b\text{.}$

In the following, the true model and the simple models *(i)* used for analysis are defined. The expectation and variance of the test *τ*^(*i*)^ used are expressed as a function of the parameters conditional on genotypes and on the variance of polygenic effects. Finally, the marginal type 1 error rate and power are given by integrating the SNP genotypes and polygenic variance estimators given the relationship matrix and the true variance parameters. It should be noted that power was calculated based on the SNP effect, not based on the effect of a QTL linked to the SNP. To calculate the power to detect a QTL, assuming LD *r*^2^ between the SNP and the QTL, the regression coefficient of the QTL effect is equal to the SNP effect divided by *r*.

### Statistical models

The true model was assumed to be the following mixed model:

y=1μ+xβ+u+e,

where **y** is the vector of the observed trait (one phenotype per animal), μ is the vector of the overall mean, *β* the regression coefficient of the fixed SNP effect, **u** the vector of random additive genetic effects of the animals and **e** the vector of random residuals. Let *E*(**u**) = **0**, *V*(**u**) = **A***σ*_*u*_^2^ with **A** being the relationship matrix and *σ*_*u*_^2^ the additive polygenic variance, and *V*(**e**) = **I***σ*_*e*_^2^ with *σ*_*e*_^2^ the residual variance. Heritability was defined as the ratio between the polygenic genetic variance and the sum of polygenic variance and residual variance: $h^{2}=\frac{\sigma_{u}^{2}}{\sigma_{u}^{2}+\sigma_{e}^{2}}$ and we defined the phenotypic variance as *σ*_*y*_^2^ = *σ*_*u*_^2^ + *σ*_*e*_^2^. The vector **x** is the incidence vector of the SNP effect, defined as $x=w-1\bar{w}$ (see for example [64]), where **w** is $-2p/\sqrt{2pq}$ for genotype 11, $\left(1-2p\right)/\sqrt{2pq}$ for genotype 12, and $2q/\sqrt{2pq}$ for genotype 22, with *p* being the frequency of allele 2 and *q* the frequency of allele 1, so that *E*(*w*) = 0 and *V*(*w*) = 1. Based on the definition of **x**, the relationship between the regression coefficient of the true model and the allele substitution effect (the difference between genotype 11 and 12 or 12 and 22) is:

$$\beta_{\mathrm{allele}}=\beta /\sqrt{2pq}\text{.}$$

So, the same statistical power was obtained for different allele substitution effects, depending on the allele frequencies. For the sake of simplicity, no other fixed effect was added to the model.

We analyzed four simpler models that were used to estimate the SNP effect instead of the true model. The first three models were association analyses and the fourth was a linkage and association analysis. The superscript *(i), i* = 1,…,4 was added to identify the effects specific to each of the four models.

1) The first model was a simple regression model with no polygenic effect:

(1) $$y=1\mu^{\left(1\right)}+x\beta^{\left(1\right)}+e^{\left(1\right)}\text{.}$$

2) The second model was the GRAMMAR method developed by [41] and [43]. GRAMMAR is a two-step method in which first the following model is fitted:

(2) $$y=1\mu^{\left(2a\right)}+u^{\left(2a\right)}+e^{\left(2a\right)}\text{,}$$

then the estimates of residuals are used to estimate the SNP effect:

(3) $${\hat{e}}^{\left(2a\right)}=1\mu^{\left(2b\right)}+x\beta^{\left(2b\right)}+e^{\left(2b\right)}\text{.}$$

3) The third model was derived from the FASTA approach from [44]. To homogenize comparisons, we did not use the score as formalized by the authors but simply considered the marker effect t-test from the following model:

(4) $$y=1\mu^{\left(3\right)}+x\beta^{\left(3\right)}+u^{\left(3\right)}+e^{\left(3\right)}\text{,}$$

but with variance components estimated from the same random model like in the first step of GRAMMAR (**y** = **1***μ*^(2*a*)^ + **u**^(2*a*)^ + **e**^(2*a*)^), i.e. with $V\left(u^{\left(3\right)}\right)=A{\hat{\sigma }}_{u^{\left(3\right)}}^{2}$ instead of $V\left(u^{\left(3\right)}\right)=A\sigma_{u^{\left(3\right)}}^{2}$ and $V\left(e^{\left(3\right)}\right)=I{\hat{\sigma }}_{e^{\left(2a\right)}}^{2}\text{.}$

4) The fourth model was the linkage analysis and association method, QTDT, developed by [13]. Let $z=\frac{x_{s}+x_{d}}{2}$, where **x**_*s*_ and **x**_*d*_ denote the genotype of the sire and dam of the animal. Then:

(5) $$y=1\mu^{\left(4\right)}+\left(z-1\bar{z}\right)\beta_{b}^{\left(4\right)}+\left(x-z\right)\beta_{w}^{\left(4\right)}+e^{\left(4\right)}\text{,}$$

where *β*_*b*_^(4)^ is the regression coefficient between families and *β*_*w*_^(4)^ the regression coefficient within families.

### Validation of the derivations

Details on the algebra used to obtain the results are provided in Additional file 1 [See Additional file 1]. Several approximations were used in the derivations, notably:

ignoring the variance of the estimator of the SNP effect caused by estimation of the variance component instead of using true variance [65],

replacing quadratic forms by their expectations in products and ratios.

Therefore, simulations were first performed to validate the formulae for each method. Validation was restricted to the family structures and heritability values used in the “Comparison of methods” section of the paper. The population used for the simulations therefore consisted of 600 genotyped individuals, offspring of 120, 20 and 10 sires that produced 5, 30 and 60 offspring, respectively. To do this, the genotypes for a SNP were simulated for sires and dams with allele frequencies of 0.5, and the genotypes of the offspring were extrapolated from their parents’ genotypes. Next, the polygenic values of the sires and offspring and the phenotypes of the offspring were computed with and without the effect of a corresponding QTL with an allele substitution effect of 0.20 (equivalent to a regression coefficient of 0.141 phenotypic standard deviations or a QTL explaining 2% of the phenotypic variance). The robustness and power of each method were then evaluated using these two phenotypes (with or without a QTL) with a significance threshold of 5% (which is different from the 1% threshold used in the application section). The simulations were performed with heritabilities ranging from 0 to 1 by 0.1 steps. 10 000 replicates were simulated for each scenario. In total, 1 320 000 simulations were performed. For the GRAMMAR and FASTA methods, the ASREML software [66] was used to estimate variance components. The relationship matrix used for these two methods was derived from pedigree data and not from genomic data. Details are provided in Additional file 2 [See Additional file 2].

An R program (see Additional file 3) was written to compute the type 1 error rate and the power of the four methods under any relationship matrix and heritability.

## Results

### Expectation and variance of the estimator of the SNP effect and of the test statistics

This section only considers the formulae for the expectation and variance of the estimator, the expectation of the sum of the squares of residuals and the expectation and variance of the test statistics. Details are provided in Additional file 1.

#### Model 1: regression model

Assuming model (1), the SNP effect was estimated by:

$${\hat{\beta }}^{\left(1\right)}={\left(x\prime x\right)}^{-}x\prime y\text{.}$$

If the vector **y** followed model (1), $E^{\left(1\right)}\left({\hat{\beta }}^{\left(1\right)}\right)=\beta$, $V^{\left(1\right)}\left({\hat{\beta }}^{\left(1\right)}\right)={\left(x\prime x\right)}^{-}\sigma_{e^{\left(1\right)}}^{2}$ and the residual variance is estimated from the sum of the squares of residuals assuming, $E^{\left(1\right)}\left({\hat{e}}^{\left(1\right)}\prime {\hat{e}}^{\left(1\right)}\right)=\left(n-2\right)\sigma_{e^{\left(1\right)}}^{2}$. But in fact, when considering that **y** follows the true model, the true expressions are as follows.

The expectation of this estimator is:

$$E\left({\hat{\beta }}^{\left(1\right)}\right)=\beta \text{.}$$

The variance of the estimator is:

$$V({\hat{\beta }}^{\left(1\right)})=\sigma_{e}^{2}\left[{\left(x\prime x\right)}^{-}+\frac{h^{2}}{1-h^{2}}{\left(x\prime x\right)}^{-}x\prime Ax{\left(x\prime x\right)}^{-}\right].$$

So the variance of the estimator of the SNP effect was a function of heritability and of the relationship matrix, in addition to the usual factor involving residual variance. The residual variance was estimated using the sum of squares of residuals. The expectation is:

$$\begin{array}{ll} E({\hat{e}}^{\left(1\right)}\prime {\hat{e}}^{\left(1\right)})= & \sigma_{e}^{2}\left[\left(n-2\right)+\frac{h^{2}}{\left(1-h^{2}\right)}\left(tr\left(A\right)\right)\right)\\ & \left(\left(-{\left(x\prime x\right)}^{-}x\prime Ax-{\left(1\prime 1\right)}^{-}1\prime A1\right)\right]\text{,} \end{array}$$

where *n* is the number of animals analyzed.

Finally, the mean and variance of the test statistic that was actually used are:

$$\begin{array}{l} E(\tau^{\left(1\right)})=\\ \frac{\left(\beta /\sigma_{y}\right)\sqrt{\left(x\prime x\right)}}{\sqrt{1+h^{2}\frac{tr\left(A\right)-{\left(x\prime x\right)}^{-}x\prime Ax-{\left(1\prime 1\right)}^{-1}1\prime A1-\left(n-2\right)}{\left(n-2\right)}}} \end{array}$$

$$\begin{array}{l} V(\tau^{\left(1\right)})=\\ \frac{1+h^{2}(x\prime Ax{\left(x\prime x\right)}^{-}-1)}{1+h^{2}\frac{tr(A)-{\left(x\prime x\right)}^{-}x\prime Ax-{\left(1\prime 1\right)}^{-1}1\prime A1-(n-2)}{n-2}}. \end{array}$$

#### Model 2: GRAMMAR model

Assuming model (2b), the SNP effect was estimated by:

$${\hat{\beta }}^{\left(2b\right)}={\left(x\prime x\right)}^{-}x\prime {\hat{e}}^{\left(2a\right)}={\left(x\prime x\right)}^{-}x\prime \left(y-1{\hat{\mu }}^{\left(2a\right)}-{\hat{u}}^{\left(2a\right)}\right)\text{.}$$

Assuming **y** followed model (2b), $V^{\left(2\right)}\left({\hat{\beta }}^{\left(2b\right)}\right)={\left(x\prime x\right)}^{-}\sigma_{e^{\left(2b\right)}}^{2}$ and $E^{\left(2\right)}\left({\hat{e}}^{\left(2b\right)}\prime {\hat{e}}^{\left(2b\right)}\right)=\left(n-2\right)\sigma_{e^{\left(2b\right)}}^{2}$. To develop the correct formulae, we need to know the expectation and variance of estimators of the polygenic effects in the random model (2a). The mixed model equation of model (2a) can be denoted as:

$$\left[\begin{array}{cc} 1\prime 1 & 1\prime \\ 1 & I+\lambda^{\left(2a\right)}A^{-1} \end{array}\right]\;\left[\begin{array}{c} {\hat{\mu }}^{\left(2a\right)}\\ {\hat{u}}^{\left(2a\right)} \end{array}\right]=\left[\begin{array}{c} 1\prime y\\ y \end{array}\right]\text{,}$$

where

$$\lambda^{\left(2a\right)}=\frac{\sigma_{e^{\left(2a\right)}}^{2}}{\sigma_{u^{\left(2a\right)}}^{2}}\;\text{and}\;\left[\begin{array}{c} {\hat{\mu }}^{\left(2a\right)}\\ {\hat{u}}^{\left(2a\right)} \end{array}\right]=\left[\begin{array}{cc} C_{11}^{\left(2a\right)} & C_{1u}^{\left(2a\right)}\\ C_{u1}^{\left(2a\right)} & C_{\mathrm{uu}}^{\left(2a\right)} \end{array}\right]\;\left[\begin{array}{c} 1\prime y\\ y \end{array}\right]\text{.}$$

Then, assuming that **y** followed the true model:

$$E\left({\hat{u}}^{\left(2a\right)}\right)=C_{\mathrm{uu}}^{\left(2a\right)}x\beta \text{.}$$

The estimates of the polygenic effects were biased, and:

$$\begin{array}{ll} V({\hat{u}}^{\left(2a\right)})= & \sigma_{u}^{2}(A-\lambda^{\left(2a\right)}C_{\mathrm{uu}}^{\left(2a\right)})\\ & +(\sigma_{e}^{2}-\lambda^{\left(2a\right)}\sigma_{u}^{2})C_{\mathrm{uu}}^{\left(2a\right)}(I-\lambda^{\left(2a\right)}A^{-1}C_{\mathrm{uu}}^{\left(2a\right)}) \end{array}$$

Thus, when computing the expectation of estimator of the SNP effect:

$$E\left({\hat{\beta }}^{\left(2b\right)}\right)=\beta -{\left(x\prime x\right)}^{-}x\prime C_{\mathrm{uu}}^{\left(2a\right)}x\beta \text{,}$$

the estimator of the SNP effect was biased,

$$\begin{array}{l} V({\hat{\beta }}^{{}^{\left(2b\right)}})=\sigma_{e}^{2}\left[{\left(x\prime x\right)}^{-}-{\left(x\prime x\right)}^{-}x\prime C_{uu}^{\left(2a\right)}x{\left(x\prime x\right)}^{-}\right]\\ -(\sigma_{e}^{2}-\lambda^{\left(2a\right)}\sigma_{u}^{2})\lambda^{\left(2a\right)}{\left(x\prime x\right)}^{-}x\prime C_{uu}^{\left(2a\right)}A^{-1}C_{uu}^{\left(2a\right)}x{\left(x\prime x\right)}^{-}, \end{array}$$

and the residual variance was estimated using the sum of squares of residuals:

$$\begin{array}{l} E({\hat{e}}^{\left(2b\right)}\prime {\hat{e}}^{\left(2b\right)})\\ =\sigma_{e}^{2}(n-2-tr(C_{uu}^{\left(2a\right)})+{\left(x\prime x\right)}^{-}x\prime C_{uu}^{\left(2a\right)}x+{\left(1\prime 1\right)}^{-}1\prime C_{uu}^{\left(2a\right)}1)\\ +\beta^{2}x\prime C_{uu}^{\left(2a\right)}(I-x{\left(x\prime x\right)}^{-}x\prime -1{\left(1\prime 1\right)}^{-}1\prime )C_{uu}^{\left(2a\right)}x\\ -(\sigma_{e}^{2}-\lambda^{\left(2a\right)}\sigma_{u}^{2})\lambda^{\left(2a\right)}\\ tr((I-x{\left(x\prime x\right)}^{-}x\prime -1{\left(1\prime 1\right)}^{-}1\prime )C_{uu}^{\left(2a\right)}A^{-1}C_{uu}^{\left(2a\right)}). \end{array}$$

Hence,

$$E\left(\tau^{\left(2b\right)}\right)=\left(\beta -{\left(x\prime x\right)}^{-}x\prime C_{\mathrm{uu}}^{\left(2a\right)}x\beta \right)\frac{\sqrt{\left(n-2\right)x\prime x}}{\sqrt{E\left({\hat{e}}^{\left(2b\right)}\prime {\hat{e}}^{\left(2b\right)}\right)}}$$

$$\begin{array}{l} V(\tau^{\left(2b\right)})=\frac{\left(n-2\right)}{E({\hat{e}}^{\left(2b\right)}{}^{\prime }{\hat{e}}^{\left(2b\right)})}(\sigma_{e}^{2}[1-x^{\prime }C_{uu}^{\left(2a\right)}x{\left(x^{\prime }x\right)}^{-}]\\ \;-(\sigma_{e}^{2}-\lambda^{\left(2a\right)}\sigma_{u}^{2})\lambda^{\left(2a\right)}x^{\prime }C_{uu}^{\left(2a\right)}A^{-1}C_{uu}^{\left(2a\right)}x{\left(x^{\prime }x\right)}^{-}). \end{array}$$

#### Model 3: FASTA model

The only difference between this model and the true model was the variance components used, which were the same as in the GRAMMAR model. The mixed model equation for model (3) is:$\left[\begin{array}{ccc} 1\prime 1 & 1\prime x & 1\prime \\ x\prime 1 & x\prime x & x\prime \\ 1 & x & I+\lambda^{\left(2a\right)}A^{-1} \end{array}\right]\;\left[\begin{array}{c} {\hat{\mu }}^{\left(3\right)}\\ {\hat{\beta }}^{\left(3\right)}\\ {\hat{u}}^{\left(3\right)} \end{array}\right]=\left[\begin{array}{c} 1\prime y\\ x\prime y\\ y \end{array}\right]$ with $\lambda^{\left(2a\right)}=\frac{\sigma_{e^{\left(2a\right)}}^{2}}{\sigma_{u^{\left(2a\right)}}^{2}}$, from the first model (2a) used in GRAMMAR.

If:

$$\begin{array}{l} \left[\begin{array}{ccc} C_{11}^{\left(3\right)} & C_{1\beta }^{\left(3\right)} & C_{1u}^{\left(3\right)}\\ C_{\beta 1}^{\left(3\right)} & C_{\beta \beta }^{\left(3\right)} & C_{\mathrm{\beta u}}^{\left(3\right)}\\ C_{u1}^{\left(3\right)} & C_{\mathrm{u\beta}}^{\left(3\right)} & C_{\mathrm{uu}}^{\left(3\right)} \end{array}\right]\;\left[\begin{array}{ccc} 1\prime 1 & 1\prime x & 1\prime \\ x\prime 1 & x\prime x & x\prime \\ 1 & x & I+\lambda^{\left(2a\right)}A^{-1} \end{array}\right]\\ =\left[\begin{array}{ccc} 1 & 0 & 0\\ 0 & 1 & 0\\ 0 & 0 & I \end{array}\right] \end{array}$$

the estimator of the SNP effect is:

$${\hat{\beta }}^{\left(3\right)}=\left(C_{\beta 1}^{\left(3\right)}1\prime +C_{\beta \beta }^{\left(3\right)}x\prime +C_{\mathrm{\beta u}}^{\left(3\right)}\right)y\text{.}$$

Assuming **y** follows model (3), $V^{\left(3\right)}\left({\hat{\beta }}^{\left(3\right)}\right)=C_{\beta \beta }^{\left(3\right)}\sigma_{e^{\left(3\right)}}^{2}$ and the sum of products between phenotypes and residuals were used to estimate the residual variance, as is customary in mixed models, so that $E^{\left(3\right)}\left(y\prime {\hat{e}}^{\left(3\right)}\right)=\left(n-2\right)\sigma_{e^{\left(3\right)}}^{2}$. Then, the expectation and variance of the estimator of the SNP effect, assuming a true model for **y**, are

$$E\left({\hat{\beta }}^{\left(3\right)}\right)=\beta$$

$$\begin{array}{l} V({\hat{\beta }}^{\left(3\right)})=\sigma_{e}^{2}{\left(x\prime x-x\prime C_{\mathrm{uu}}^{\left(2a\right)}x\right)}^{-1}-(\sigma_{e}^{2}-\lambda^{\left(2a\right)}\sigma_{u}^{2})\\ {\left({\left(x\prime x-x\prime C_{\mathrm{uu}}^{\left(2a\right)}x\right)}^{-1}\right)}^{2}\left(x\prime C_{\mathrm{uu}}^{\left(2a\right)}x-x\prime C_{\mathrm{uu}}^{\left(2a\right)}C_{\mathrm{uu}}^{\left(2a\right)}x\right) \end{array}$$

$$\begin{array}{l} E({\hat{e}}^{\left(3\right)}\prime y)=(n-2)\sigma_{e}^{2}-(\sigma_{e}^{2}-\sigma_{u}^{2}\lambda^{\left(2a\right)})\\ \left(\begin{array}{l} tr(C_{\mathrm{uu}}^{\left(2a\right)})-\frac{1}{n}1\prime C_{\mathrm{uu}}^{\left(2a\right)}1\\ -\frac{\left(x\prime C_{\mathrm{uu}}^{\left(2a\right)}x+\frac{1}{n}x\prime C_{\mathrm{uu}}^{\left(2a\right)}11\prime C_{\mathrm{uu}}^{\left(2a\right)}x-x\prime C_{\mathrm{uu}}^{\left(2a\right)}C_{\mathrm{uu}}^{\left(2a\right)}x\right)}{\left(x\prime x-x\prime C_{\mathrm{uu}}^{\left(2a\right)}x\right)} \end{array}\right)\text{.} \end{array}$$

Hence,

$$E\left(\tau^{\left(3\right)}\right)=\beta \sqrt{x\prime x-x\prime C_{\mathrm{uu}}^{\left(2a\right)}x}\frac{\sqrt{\left(n-2\right)}}{\sqrt{E\left({\hat{e}}^{\left(3\right)}\prime y\right)}}$$

$$\begin{array}{ll} V(\tau^{\left(3\right)})= & \left(\sigma_{e}^{2}-\left(\sigma_{e}^{2}-\lambda^{\left(2a\right)}\sigma_{u}^{2}\right)\frac{\left(x\prime C_{\mathrm{uu}}^{\left(2a\right)}x-x\prime C_{\mathrm{uu}}^{\left(2a\right)}C_{\mathrm{uu}}^{\left(2a\right)}x\right)}{\left(x\prime x-x\prime C_{\mathrm{uu}}^{\left(2a\right)}x\right)}\right)\\ & \times \left(\frac{n-2}{E\left({\hat{e}}^{\left(3\right)}\prime y\right)}\right)\text{.} \end{array}$$

#### Model 4: QTDT model

Assuming model (4), two regression coefficients had to be estimated:

$$\begin{array}{l} \left[\begin{array}{c} {\hat{\mu }}^{\left(4\right)}\\ {\hat{\beta }}_{b}^{\left(4\right)}\\ {\hat{\beta }}_{w}^{\left(4\right)} \end{array}\right]=\\ {\left[\begin{array}{ccc} 1\prime 1 & 1\prime \left(z-1\bar{z}\right) & 1\prime \left(x-z\right)\\ \left(z-1\bar{z}\right)\prime 1 & \left(z-1\bar{z}\right)\prime \left(z-1\bar{z}\right) & \left(z-1\bar{z}\right)\prime \left(x-z\right)\\ \left(x-z\right)\prime 1 & \left(x-z\right)\prime \left(z-1\bar{z}\right) & \left(x-z\right)\prime \left(x-z\right) \end{array}\right]}^{-}\left[\begin{array}{c} 1\prime y\\ \left(z-1\bar{z}\right)\prime y\\ \left(x-z\right)\prime y \end{array}\right]\text{.} \end{array}$$

If $\hat{\theta }=\left[\begin{array}{c} {\hat{\mu }}^{\left(4\right)}\\ {\hat{\beta }}_{b}^{\left(4\right)}\\ {\hat{\beta }}_{w}^{\left(4\right)} \end{array}\right]={\left(Q\prime Q\right)}^{-}Q\prime y$ with $Q=\left[\begin{array}{ccc} 1 & \left(z-1\bar{z}\right) & \left(x-z\right) \end{array}\right]$_,_

assuming model (4), the variance and expectation of the estimators are:

$$V^{\left(4\right)}\left(\hat{\theta }\right)={\left(Q\prime Q\right)}^{-}\sigma_{e^{\left(4\right)}}^{2}\;\text{and}\;E^{\left(4\right)}\left({\hat{e}}^{\left(4\right)}\prime {\hat{e}}^{\left(4\right)}\right)=\left(n-3\right)\sigma_{e^{\left(4\right)}}^{2}\text{,}$$

and assuming the true model, the expectation and variance of estimates of the regression coefficients are in fact:

$$E\left(\hat{\theta }\right)={\left(Q\prime Q\right)}^{-}Q\prime \left(x\beta +1\mu \right)$$

$$V(\hat{\theta })={\left(Q\prime Q\right)}^{-}Q\prime AQ{\left(Q\prime Q\right)}^{-}\sigma_{u}^{2}+{\left(Q\prime Q\right)}^{-}\sigma_{e}^{2},$$

and the sum of the squares of residuals:

$$\begin{array}{ll} \text{E}({\hat{e}}^{\left(4\right)}\prime {\hat{e}}^{\left(4\right)})= & (n-3)\sigma_{e}^{2}+\left(tr\left(A\right)-tr\left(Q{\left(Q\prime Q\right)}^{-}Q\prime A\right)\right)\sigma_{u}^{2}\\ & +\mu^{2}(n-1\prime Q{\left(Q\prime Q\right)}^{-}Q\prime 1)\\ & +\beta^{2}(x\prime x-x\prime Q{\left(Q\prime Q\right)}^{-}Q\prime x)\\ & -\mu \beta (1\prime Q{\left(Q\prime Q\right)}^{-}Q\prime x+x\prime Q{\left(Q\prime Q\right)}^{-}Q\prime 1)\text{.} \end{array}$$

Thus, the expectation and variance of the test on ${\hat{\beta }}_{w}^{\left(4\right)}$ (the within-family regression) is:

$$\text{E}\left(\tau^{\left(4\right)}\right)=\frac{{\left[{\left(Q\prime Q\right)}^{-}Q\prime \left(x\beta +1\mu \right)\right]}_{3,1}}{\sqrt{{\left[{\left(Q\prime Q\right)}^{-}\right]}_{3,3}}}\frac{\sqrt{\left(n-3\right)}}{\sqrt{E\left({\hat{\text{e}}}^{\left(4\right)}\prime {\hat{\text{e}}}^{\left(4\right)}\right)}}$$

$$\begin{array}{l} V(\tau^{\left(4\right)})=\\ {}[\frac{{\left(Q\prime Q\right)}^{-}Q\prime AQ{\left(Q\prime Q\right)}^{-}\sigma_{u}^{2}+{\left(Q\prime Q\right)}^{-}\sigma_{e}^{2}{}_{3,3}}{{\left[{\left(Q\prime Q\right)}^{-}\right]}_{3,3}}\text{,}\frac{\left(n-3\right)}{E({\hat{e}}^{\left(4\right)}\prime {\hat{e}}^{\left(4\right)})}, \end{array}$$

where [**M**]_3,3_ denotes the coefficient of line 3 and column 3 that of matrix **M.**

#### True model

With the true model, the classical formulae are:

$$E\left(\hat{\beta }\right)=\beta \text{,}$$

$$V\left(\hat{\beta }\right)=\sigma_{e}^{2}C_{\beta \beta }\text{,}$$

$$E\left(\hat{\text{e}}\prime y\right)=\left(n-2\right)\sigma_{e}^{2}\text{,}$$

$$E\left(\tau \right)=\frac{\beta }{\sigma_{e}\sqrt{C_{\beta \beta }}}\text{,}$$

$$V\left(\tau \right)=1.$$

### Marginal expectation and variance of test statistics

The above formulae give the conditional expectation of the estimators of the SNP effects and the conditional expectation and variance of test statistics based on specific data, i.e., given **w**, the marker genotypes (or **x,** the centered genotypes defined in the true model) and the known variance component of the polygenic effects. These formulae can be applied to any kind of data.

The aim of this section is to derive the marginal expectation and variance of the test statistics, by integrating over the distribution of genotypes and the variance components of the random polygenetic effects, given the relationship matrix and variance components of the true model. For this purpose, the quadratic forms involving **x** and **z** and the variance components of the random model (2) were replaced by their expectation. If *E*_*x*_ denotes these expectations and *a*_*ij*_ is the relationship coefficient between animals *i* and *j,* then the relationship coefficient for the Mendelian sampling variance *d*_*ii*_ can be defined as:

$$d_{\mathrm{ii}}=a_{\mathrm{ii}}-\left(\frac{1}{4}a_{s_{i}s_{i}}+\frac{1}{4}a_{d_{i}d_{i}}+\frac{1}{2}a_{s_{i}d_{i}}\right)$$

where *s*_*i*_ is the sire of animal *i* and *d*_*i*_ the dam.

**D** is the diagonal matrix with elements *d*_*ii*_. Assuming Hardy Weinberg equilibrium, we know that [67]:*E*_*x*_(*w*_*i*_*w*_*j*_) = *a*_*ij*_, *E*_*x*_(*w*_*i*_*w*_*i*_) = *a*_*ii*_, *E*_*x*_(*w*_*i*_*z*_*i*_) = *E*_*x*_(*z*_*i*_*z*_*i*_) = *a*_*ii*_ − *d*_*ii*_, and *E*_*x*_(*z*_*i*_*z*_*j*_) = *a*_*ij*_,when the genotype, **w**, is expressed in a standardized form, as shown in the introduction. Thus:

$$E_{x}\left(x\prime x\right)=tr\left(A\right)-\frac{1}{n}1\prime A1$$

$$E_{x}\left(x\prime Ax\right)=tr\left(AA\right)-\frac{2}{n}1\prime AA1+\frac{1}{n^{2}}1\prime A11\prime A1$$

$$\begin{array}{ll} E_{x}(x\prime C_{\mathrm{uu}}^{\left(2a\right)}x)= & tr(A)-\lambda^{\left(2a\right)}tr(C_{\mathrm{uu}}^{\left(2a\right)})\\ & +\frac{1}{n^{2}}1\prime A11\prime C_{\mathrm{uu}}^{\left(2a\right)}1-\frac{1}{n}1\prime AC_{\mathrm{uu}}^{\left(2a\right)}1 \end{array}$$

$$\begin{array}{l} E_{x}(x\prime C_{\mathrm{uu}}^{\left(2\right)}C_{\mathrm{uu}}^{\left(2\right)}x)=\\ tr(A)-\lambda^{\left(2\right)}tr(C_{\mathrm{uu}}^{\left(2a\right)})-\lambda^{\left(2a\right)}tr(C_{\mathrm{uu}}^{\left(2a\right)}C_{\mathrm{uu}}^{\left(2a\right)})\\ +\frac{1}{n}1\prime AC_{\mathrm{uu}}^{\left(2a\right)}1-\frac{1}{n}1\prime AC_{\mathrm{uu}}^{\left(2a\right)}C_{\mathrm{uu}}^{\left(2a\right)}1+\frac{1}{n^{2}}1\prime A11\prime C_{\mathrm{uu}}^{\left(2a\right)}C_{\mathrm{uu}}^{\left(2a\right)}1\\ E_{x}(x\prime C_{\mathrm{uu}}^{\left(2a\right)}11\prime C_{\mathrm{uu}}^{\left(2a\right)}x)\\ =1\prime A1-\lambda^{\left(2a\right)}1\prime C_{\mathrm{uu}}^{\left(2a\right)}1\\ -\lambda^{\left(2a\right)}1\prime C_{\mathrm{uu}}^{\left(2a\right)}C_{\mathrm{uu}}^{\left(2a\right)}1+\frac{1}{n}\lambda^{\left(2a\right)}1\prime C_{\mathrm{uu}}^{\left(2a\right)}11\prime C_{\mathrm{uu}}^{\left(2a\right)}1\text{,} \end{array}$$

and for the sums involved in the QTDT (as in [13]):

$$E_{x}\left(Q\prime Q\right)=\left[\begin{array}{ccc} n & 0 & 0\\ 0 & tr\left(A\right)-\frac{1}{n}1\prime A1-tr\left(D\right)\frac{n-1}{n} & 0\\ 0 & 0 & tr\left(D\right) \end{array}\right]$$

$$E_{x}\left(Q\prime x\right)=\left[\begin{array}{c} 0\\ tr\left(A\right)-\frac{1}{n}1\prime A1-tr\left(D\right)\frac{n-1}{n}\\ tr\left(D\right)\frac{n-1}{n} \end{array}\right]$$

$$E_{x}\left(Q\prime 1\right)=\left[\begin{array}{c} n\\ 0\\ 0 \end{array}\right]$$

$$\begin{array}{l} \;E_{x}(Q\prime AQ)=\\ \left[\begin{array}{ccc} 1\prime A1 & 0 & 0\\ 0 & \begin{array}{l} tr\left(A\prime A\right)-tr\left(AD\right)-\frac{2}{n}\left(1\prime A\prime A1-1\prime AD1\right)\\ \;+\frac{1}{n^{2}}\left(1\prime A11\prime A1-1\prime A1tr\left(D\right)\right) \end{array} & 0\\ 0 & 0 & tr\left(AD\right) \end{array}\right]\text{.} \end{array}$$

These expectations replace their corresponding terms in the preceding formulae in order to express all expectations and variances of the tests used for detection of the SNP effect as a function of heritability and the relationship matrix. To this end, the following approximations were made: expectations of ratios and products were replaced by ratios and products of expectations. For the expectation of variance components given the relationships, the following expectations were used:

$$E_{x}\left(\sigma_{u^{\left(2\right)}}^{2}\right)=\sigma_{u}^{2}+\beta^{2}\text{,}$$

$$E_{x}\left(\sigma_{e^{\left(2\right)}}^{2}\right)=\sigma_{e}^{2}\text{,}$$

$$E_{x}\left(\lambda^{\left(2\right)}\right)=\frac{\sigma_{e}^{2}}{\sigma_{u}^{2}+\beta^{2}}\text{.}$$

### Validation of deterministic formulae

As pointed out in the Methods section, simulations were performed in order to validate the previous analytical results. Table 1 summarizes these results as absolute deviations of type 1 error rate and power from analytical results. Details and standard errors of these simulations are provided in Additional file 2 [See Additional file 2]. The average deviation of simulation results from analytical results were in general of the same order as the standard deviation of the simulation results (0.22% for 5% estimated) for type 1 error rate and slightly higher for power (0.36% for example for 85% estimated). Thus, simulations were in very good agreement with analytical results and make a general discussion possible. The only exception may be for extremely high values of heritability.

**Table 1:** Average and maximum absolute differences for type 1 error rate and power between simulated* and theoretical results

	**Model**			
	**Regression**	**GRAMMAR**	**FASTA**	**QTDT**
**Type 1 error (%)**				
Average difference	0.26	0.22	0.18	0.28
Maximum difference	1.09	0.70	0.50	0.74
**Power (%)**				
Average difference	0.37	0.58	0.90	1.12
Maximum difference	0.76	2.97	1.74	2.19

*see text: total population size = 600 with 120, 20 and 10 sires that respectively produced 5, 30 and 60 offspring, MAF of SNP 0.5, QTL effect 2% of phenotypic variance, assumed type 1 error rate 5%, heritability from 0 to 1 in steps of 0.1.

### Comparison of methods

The above formulae can be applied to any data without simulation when the relationship matrix is known. The results presented here are an illustration based on 600 recorded and genotyped progenies belonging to 120, 20 and 10 families of respectively *n*_*d*_ = 5, 30 and 60 half-sibs, which is typical for animal breeding data. The power was calculated for a SNP with a regression coefficient of 0.14 in phenotypic standard deviations (or 2% of phenotypic variance, which is equivalent to an allele substitution effect of 0.20 for a minor allele frequency (MAF) of 50% or an effect of 0.33 for a MAF of 10%. The effect of changes in the total number of animals, and estimates of variance components used in GRAMMAR and FASTA was also analyzed.

For families of half-sibs, the preceding formulae concerning expectation and variance of the tests throughout the Results section were calculated using:$c_{\mathrm{ii}}=\frac{h^{2}\left[16+h^{2}\left(3n_{d}-7\right)\right]}{\left(4-h^{2}\right)\left(4+h^{2}\left(n_{d}-1\right)\right)}+\frac{{\left(h^{2}\left(n_{d}+3\right)\right)}^{2}}{4n\left(1-h^{2}\right)\left(4+h^{2}\left(n_{d}-1\right)\right)}$, the diagonal term of **C**_*uu*_^(2)^, $c_{\mathrm{ij}}=\frac{4\left(1-h^{2}\right)h^{2}}{\left(4-h^{2}\right)\left(4+h^{2}\left(n_{d}-1\right)\right)}+\frac{{\left(h^{2}\left(n_{d}+3\right)\right)}^{2}}{4n\left(1-h^{2}\right)\left(4+h^{2}\left(n_{d}-1\right)\right)}$, the off-diagonal term of **C**_*uu*_^(2)^ between half-sibs, $c_{\mathrm{ij}}=\frac{{\left(h^{2}\left(n_{d}+3\right)\right)}^{2}}{4n\left(1-h^{2}\right)\left(4+h^{2}\left(n_{d}-1\right)\right)}$ the off diagonal term of **C**_*uu*_^(2)^ between animals from different families. Diagonal coefficients of the relationship matrix **A** were 1 and off-diagonal coefficients were Â¼ between half-sibs and 0 elsewhere. Matrix **D** was diagonal with coefficients ½. It should be noted that with families of equal sizes:

$$\begin{array}{ll} \lambda^{\left(2\right)}C_{\mathrm{uu}}^{\left(2\right)}A^{-1}C_{\mathrm{uu}}^{\left(2\right)} & =C_{\mathrm{uu}}^{\left(2\right)}-C_{\mathrm{uu}}^{\left(2\right)}C_{\mathrm{uu}}^{\left(2\right)}+\frac{1}{n}C_{\mathrm{uu}}^{\left(2\right)}11\prime C_{\mathrm{uu}}^{\left(2\right)}\\ & =C_{\mathrm{uu}}^{\left(2\right)}-C_{\mathrm{uu}}^{\left(2\right)}C_{\mathrm{uu}}^{\left(2\right)}. \end{array}$$

**Figure 1:**
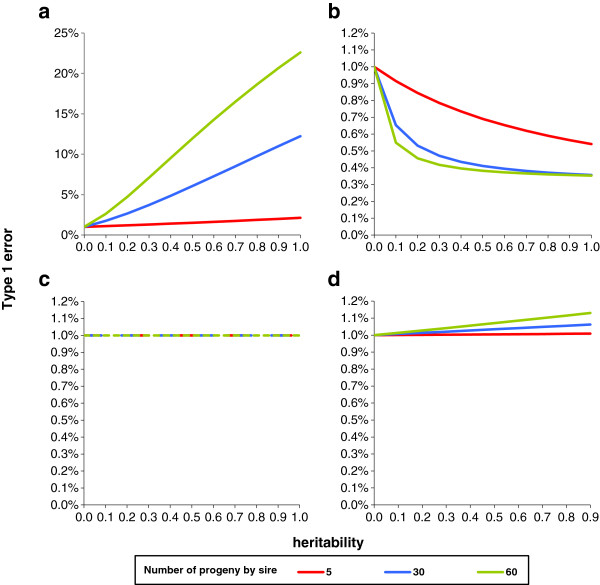
**True type 1 error rate for an assumed type 1 error rate of 1% with equal half-sib families in a sample of 600 animals for different models.a**: regression model; **b**: GRAMMAR model; **c**: FASTA model; **d**: QTDT model.

For an assumed type 1 error rate of 1%, the expected true type 1 error rate is plotted in Figures 1a to 1d according to the heritability of the polygenic effect and the number of half-sibs per family, for the same overall number of genotyped animals (600). For the regression model, there was a marked increase in type 1 error rate with heritability and family size; the type 1 error rate was equal to 12% with *h*^2^ = 0.50 and families of 60 half-sibs. With the GRAMMAR model, the type 1 error rate decreased with heritability and family size. FASTA and QTDT models were practically not affected by polygenic variance and relationships.

**Figure 2:**
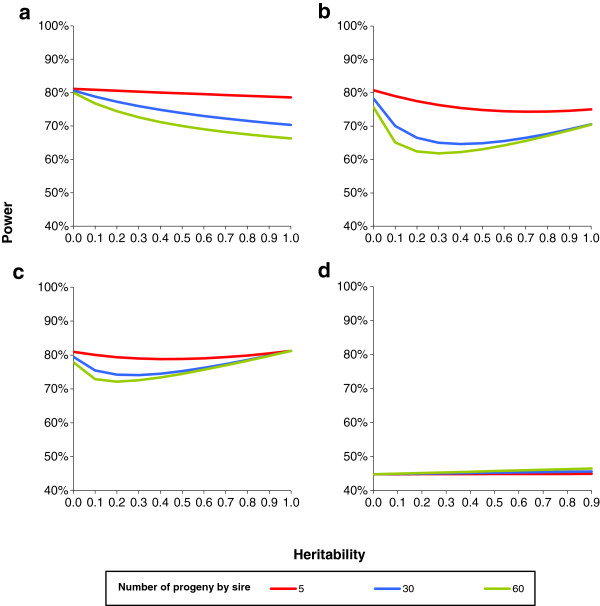
**Power with an assumed type 1 error rate of 1% in the case of equal half-sib families in a sample of 600 animals.** The following conditions were tested i.e. true regression coefficient of 0.20 phenotypic standard deviation (*σ*_*y*_) with minimum allele frequency (MAF) of 50%, which is equivalent to 0.33 *σ*_*y*_with MAF 10% or equivalent to 2% of phenotypic variance for **a**: regression model; **b**: GRAMMAR model; **c**: FASTA model; **d**: QTDT model.

Figures 2a to 2d show the power of the methods. With the regression method, the power decreased with heritability and family size. With both the FASTA and GRAMMAR methods, the power first decreased to a minimum at a heritability of about 0.30 and then increased with heritability towards a value equal to the power obtained with a heritability of 0. The power was always higher with smaller families. The power of the QTDT method was not affected by population structure or by the polygenic effect but was very low compared to the other models.

**Figure 3:**
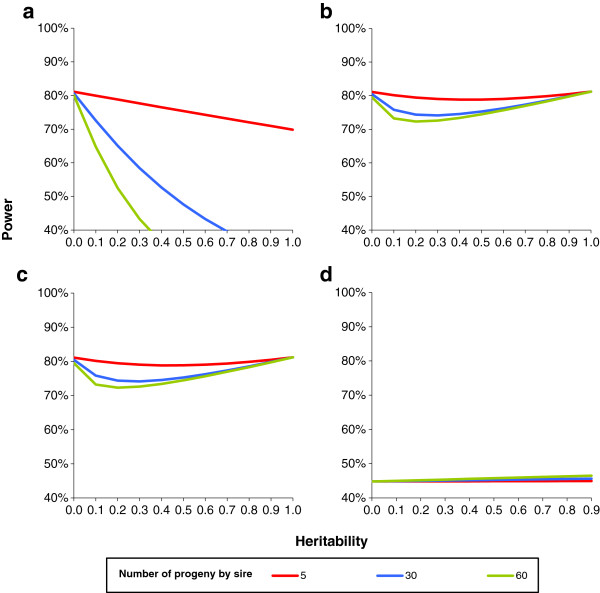
**Power with a true type 1 error rate of 1% in the case of equal half-sib families in a sample of 600 animals.** The following conditions were tested i.e. true regression coefficient of 0.20 phenotypic standard deviation (*σ*_*y*_) with minimum allele frequency (MAF) of 50%, which is equivalent to 0.33 *σ*_*y*_ with MAF 10% or equivalent to 2% of phenotypic variance for **a**: regression model; **b**: GRAMMAR model; **c**: FASTA model; **d**: QTDT model.

The power was also calculated for the same true type 1 error rate (Figure 3). In that case, the power of the regression model was always lower than that of the FASTA model, which was equal to the power of the GRAMMAR model. The power of the true mixed model is not shown in Figure 3 because it was almost the same as the power of the FASTA model, except for very low heritabilities and large families (for example, for h^2^ = 0.10 and a family size of 60 half-sibs, the power of the FASTA model and the true mixed model were 73.2% and 73.3%, respectively).

**Figure 4:**
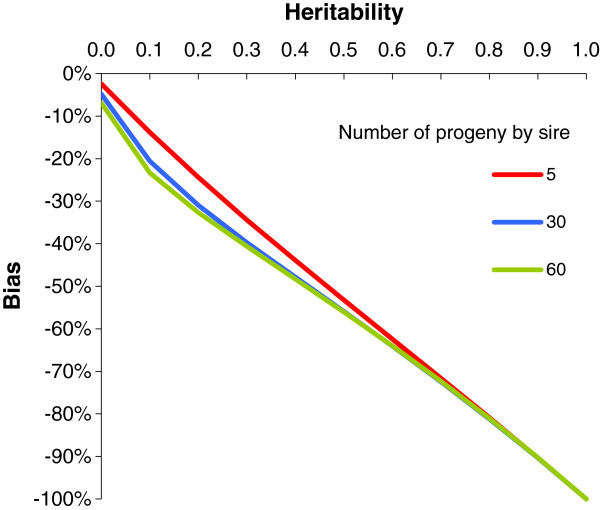
Bias of the GRAMMAR model as a percentage of true SNP effect in the case of equal half-sib families in a sample of 600 animals.

Only the GRAMMAR model resulted in biased estimators of the SNP effect and this is plotted in Figure 4. The value of the SNP effect was underestimated and the bias increased sharply as heritability increased (−56% for *h*^2^ = 0.50 and families of 60 half-sibs).

Robustness did not deviate greatly with total sample size. For example, with the regression method and for *h*^2^ = 0.50, families of 60 half-sibs and an assumed type 1 error rate of 1%, true type 1 error rate was 11.9% with a total sample of 600 animals and 12.6% with 6000 animals. With the same data structure and the GRAMMAR method, type 1 error rate was 0.38% with 600 animals and 0.35% with 6000 animals.

**Figure 5:**
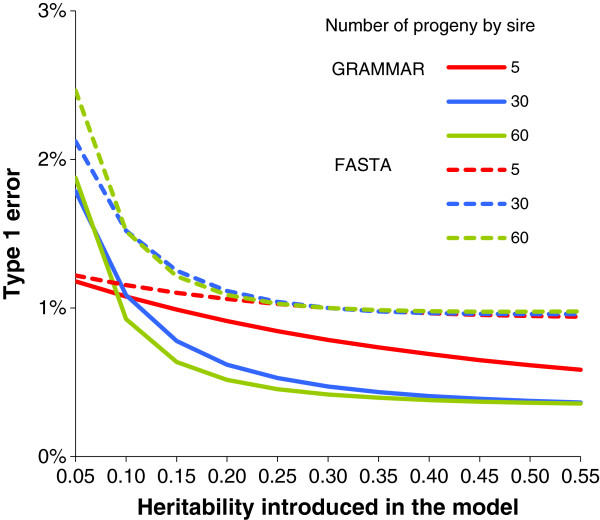
**True type 1 error rate for an assumed type 1 error rate of 1% with equal half-sib families in a sample of 600 animals with erroneous heritability.** The calculations were done with the GRAMMAR model and the FASTA model with erroneous heritability introduced in the models and a true heritability of 0.30.

For both the GRAMMAR and FASTA methods, the final models use variance components that are estimated with the same simple random model. Results presented in the previous section were marginal expectations using the distribution of the estimator of the variance components. However, one question is: what happens if the variance components are not estimated using the same sample as that used to estimate the SNP effect? Heritability can be introduced in the model if the user considers that a better estimate of heritability was obtained using other data. Figure 5 shows type 1 error rate for the GRAMMAR and FASTA methods assuming that true heritability was 0.30 but that the heritability used in models (2b) and (3) was under- or overestimated by 0.05 to 0.55. In the case of underestimated heritability, the type 1 error rate increased with decreasing heritability. For example, consider a large family (60 half-sibs) and a much smaller heritability than the true value (0.05 vs. 0.30). In that case, assuming a 1% type 1 error rate, the expected true type 1 error rate reached 1.9% for the GRAMMAR model, and 2.5% for the FASTA model.

Our algebraic results can be used as a tool to design populations or estimate the success of a given design before starting the genotyping process. FASTA statistics, which are not subject to type 1 error rate due to population stratification, should be used for this purpose.

**Figure 6:**
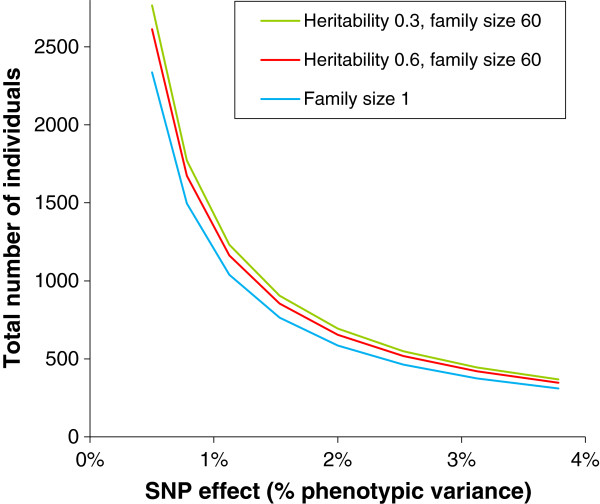
Population size required to reach 80% power with a 1% type 1 error, as a function of the SNP effect and heritability (half-sib families of 60 individuals) using the FASTA model.

**Figure 7:**
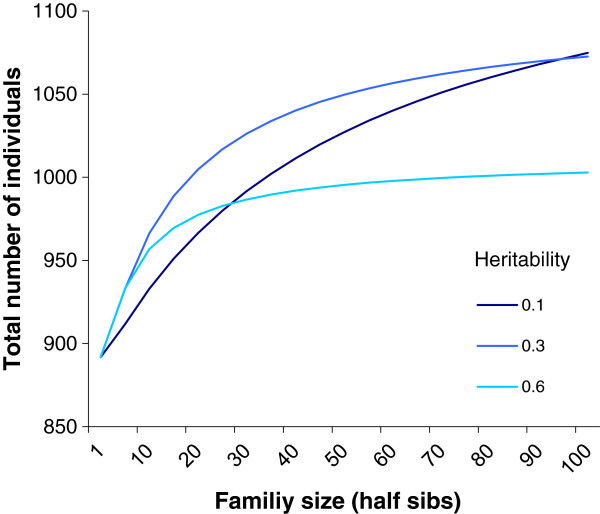
Population required to reach 80% power with a 1% type 1 error rate with the FASTA model, with the SNP effect explaining 2% of phenotypic variance as a function of family size with different polygenic heritabilities.

As shown in Figure 6, the power of the method mainly depends on the total number of individuals included in the analysis. Although power is only marginally affected by the family structure of the data and by the heritability of the trait, the experimenter may be limited (e.g., for budgetary reasons) to a fixed total size and may consequently only be able to adjust family structure. Figure 7 shows how the total population size should be adjusted to obtain a power of 80% for a given family structure for a SNP with a moderate effect of 2% of the phenotypic variance. Results show a difference of 183 individuals between the least and most favorable situations. Although this difference may appear reasonable, 183 individuals represented one fifth of the genotyping costs.

**Figure 8:**
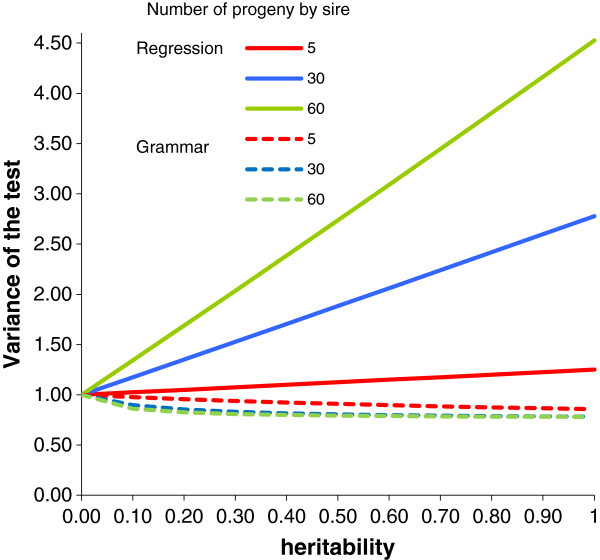
Variance of the test, or genomic control (GC) inflation factor, for the regression model and the GRAMMAR model, under the null hypothesis of no SNP effect, in the case of equal half-sib families in a sample of 600 animals.

The genomic control (GC) inflation factor developped by Delvin and Roeder [24] is a very common measure of the deviation of a test’s empirical distribution from its theoretical distribution in association studies. As pointed out by Bacanu et al. [25], in the case of multiple Student t tests for quantitative traits, the GC inflation factor may be interpreted as the variance of the normal distribution approximately followed by the Student t distribution. Even if, as presented here, the expectation of the test’s distribution is influenced by population structure under the alternate hypothesis, its variance is closely related to the GC inflation factor. Figure 8 presents the GC inflation factor, as approximated by this variance as a function of heritability and family structure. It clearly shows that inflation is very limited with the GRAMMAR method but may be considerable with the regression method when families are large and heritability is high.

## Discussion

The formulae presented in the Methods section of this paper are not easy to interpret. In the following, we explain the behavior of each method in common terms.

### Regression method

The high type 1 error rate with high heritability for this method was caused by the probability of two half-sibs sharing the same SNP because of their relationship, rather than the effect of a common QTL genotype. If a polygenic effect is present, this local similarity in SNP is confounded with the similarity of relatives in phenotype due to the polygenic effect. The expectation of polygenic effect is null. Thus, the expectation of the estimate of SNP effect is not affected by this confusion between SNP and polygenes: the test is unbiased. However, the variance of the test increases according to the variability of the relationship level in the data. If all animals in the sample share the same level of relationship (e.g. all sample are half-sibs of the same family), they would all have a similar phenotype and the same probability of sharing the same SNP. Therefore, the increase in type 1 error rate was not caused by close relationships between genotyped animals but by the presence of a mixture of close and more distant relationships. This occurs when independent large families (half-sibs, full-sibs) are present in the data. The effect of this family structure on the variance of the test was proportional to the ratio of the polygenic variance and residual variance and hence increased exponentially with heritability. However, the increase in type 1 error rate with heritability and family size did not systematically result in an increase in power. Under the alternate hypothesis (*β* = *b*), the variance of the test was still higher than 1 and increased with heritability, while the expectation of the test did not vary greatly with heritability. So when the threshold chosen for type 1 error rate (*t*_*α*/2_) was lower than the expectation of the test (power greater than 50%), a smaller proportion of the normal distribution is expected to be greater than *t*_*α*/2_ as heritability increases. This explains why the power for the regression method decreased with heritability and the variance of relationships.

### GRAMMAR model

In the GRAMMAR model, differences in the type 1 error rate and power with respect to heritability were due to the relationships between animals that were used to simultaneously estimate the polygenic effects and the SNP effect. In this case, the variance of the new phenotype, i.e. the residual of model (2a), used to test the SNP effect was approximately equal to the residual variance of the true model minus the genetic variance times (1 minus the reliability of estimates of polygenic effects). Reliability is defined as the square correlation between estimate of polygenic effect and true effect. However, due to the covariance between estimates of the polygenic effect of relatives, which are also likely to share the same SNP genotype, the variance of $\hat{\beta }$ was proportional to the residual variance of the true model times (1 minus the reliability of estimates of polygenic effects). The difference in these evolutions of the variance of the new phenotype and variance of $\hat{\beta }$ as a function of heritability explained the decrease in the variance of the test for a medium value of heritability and hence the decrease in type 1 error rate. The fact that the GRAMMAR estimate effect was greatly biased (and the only one to be so in this comparison of models) did not play a role in the changes in power with heritability, compared to these changes in the variances. If most phenotypes used to estimate the polygenic value of the animal were those of animals that were not genotyped and that had no relationships with the other genotyped animals, the GRAMMAR test would not show these type 1 error rate and power patterns. This may be the case when unrelated genotyped sires are analyzed and their phenotypes are the mean phenotype of non-genotyped progeny. In this case, the estimator of the SNP effect would still be biased downwards but the type 1 error rate and power would be practically unaffected by heritability and family structure.

### FASTA model

The only difference between the FASTA model and the true mixed model is the error in variance components since they were estimated with a pure random model. Therefore, under the null hypothesis, i.e. without a SNP effect, the variance components were the same and the type 1 error rate was not affected by heritability of the trait or relationships within the sample. Under the alternate hypothesis, the influence of heritability on power was only moderate and affected only with low to medium heritabilities. This is caused by the variance of $\hat{\beta }$_,_ which depends on the reliability of the estimates of polygenic effects, due to the mixed model ($V^{\left(3\right)}\left({\hat{\beta }}^{\left(3\right)}\right)=\sigma_{e^{\left(3\right)}}^{2}/\left[n\left(1-reliability\right)\right]$). This was particularly important when reliability differed from heritability and thus when $V\left(\hat{\beta }\right)$ decreased more rapidly than residual variance as a function of heritability. This was the case when half-sibs affected the reliability of estimates of polygenic effects, i.e. when heritability was low. As heritability increased, the reliability tended to heritability so the power became less sensitive to changes in heritability and equaled the power observed without a polygenic effect. These differences were observed both with the true mixed model and the FASTA model. The error in the estimation of heritability via the two-step procedure in FASTA had only a very small effect on these differences and was noticeable only for low heritabilities, in which case estimation errors for heritability were higher.

### QTDT method

As the QTDT method uses information within families, the variance of ${\hat{\beta }}_{w}$ was not affected by the relationships that exist between phenotyped animals in the dataset. The variance of ${\hat{\beta }}_{w}$ depended only on the trace of Mendelian sampling variance matrix and thus possibly on inbreeding within the data but not on relationships between phenotyped animals (regardless of the type of family, assuming the genotypes of the parents are known). The expectation of $V\left({\hat{\beta }}_{w}^{\left(4\right)}\right)$ integrating a given relationship matrix over SNP genotypes [See Additional file 1] without inbreeding, is $\frac{1}{n/2}\left(\sigma_{u}^{2}+\sigma_{e}^{2}\right)=\frac{2}{n}\sigma_{y}^{2}$. So, the polygenic effect has no influence on this variance. For the same reason, power was not affected by heritability or by the relationship matrix. However, power of the qdtd method was much lower than that of the other models because the test uses only half the genetic variance (only the Mendelian sampling variance). This reduced the expectation of the test by a factor $\sqrt{2}:E\left(\tau^{\left(4\right)}\right)≃\sqrt{n/2}\beta /\sigma_{y}$ and consequently decreased power.

### Comparison between methods

Type 1 error rate increased with relationships and heritability with the regression method, decreased with the GRAMMAR method, and was not affected by heritability with the FASTA and QTDT methods. The power calculated with an assumed type 1 error rate (not the real type 1 error rate) was higher with the regression method than with the FASTA method for low (large families) to moderate (small families) heritability values. Power was always lower with the GRAMMAR method than with the fasta METHOD. However, for the same true type 1 error rate (i.e. with the threshold chosen to reach the same true type 1 error rate), power was always lower with the regression method than with the FASTA method and decreased very rapidly with heritability and family size. In this situation, powers of the GRAMMAR and FASTA methods were identical. Thus, using the true type 1 error rate, these two methods have the same power. The power of the two methods was also almost identical to that of the true mixed model, except for very low heritabilities, for which a very slight difference was observed between the FASTA and true mixed models.

These results are in general agreement with the few papers on the subject that are present in the literature. Using a simple example with three pedigrees, [41] demonstrated that the type 1 error rate of the regression method increased with heritability and family size (from unrelated small nuclear families to a mixture of half- and full-sib families in pig-type pedigrees), while the opposite was observed with the GRAMMAR method, which is fully consistent with our Figures 1a and 1b. The authors also ranked the methods in order of decreasing empirical power: FASTA > GRAMMAR > regression > TDT, and found very little difference in power between the true model and the FASTA method. Using a limited range of family sizes from 1 to 4, Zhang et al. [48] found that the power of the QTDT method increased with family size, a result that is in agreement with the slight increase we observed for increases in family size from 5 to 60. Erbe et al. [54] confirmed that the GRAMMAR method allowed for better control of the type 1 error rate than the regression method, and found that in a population of 500 progenies, the type 1 error rate was greater when the progeny came from 25 rather than from 250 sires.

Therefore, as a general result, we do not recommend the regression and GRAMMAR models but do recommend the FASTA method. The FASTA method is very close to the full mixed model but is expected to be computationally faster. However, situations do exist for which the first two methods are preferred and using the FASTA method could be dangerous. The advantage of the regression model is that no heritability is required, so it could be useful when heritability is unknown or when the number of animals is too low to estimate heritability based on the data. The regression method may also be useful in situations in which having a large type 1 error rate is not a problem, for example if the objective is to first select markers before performing another type of analysis, since here the aim is to select only good markers, regardless of the number of bad ones. The advantage of the GRAMMAR method is that it has the same power as the FASTA method when corrected for underestimation of the type 1 error rate and that it allows derivation of empirical p-values, as residuals can be permuted. Correcting for underestimation of the type 1 error rate can be performed easily using analytical formulae or by analyzing the QQ plot [43], which would allow for a faster analysis than with the FASTA model. Moreover, if the GRAMMAR method uses an estimate of the polygenic heritability from another experiment and from animals that have no relationships with other genotyped animals, the GRAMMAR method is as robust and powerful as the FASTA method. Concerning the situations in which use of FASTA could be dangerous, the FASTA (and GRAMMAR) method depends on the variance components that are introduced. The difference between the expected heritability estimated in the pure random model and the true one was small when the fixed SNP effect was small, so the final effect of this error in the heritability used was not significant (the low performance of the GRAMMAR method was due to the use of residuals, not to the error on heritability). This explains why the FASTA method is close to the full mixed model (in type 1 error rate and power). However, what would happen if a variance component other than the one estimated in the sample was used or if fortuitously, the variance component given by the sample was very different from the true one? What happens to the conditional distribution of the test when using an incorrect heritability? In this case, the coefficient in the GRAMMAR method involving the difference in heritability is important and increases the variance of the test. The difference between true and used heritabilities produces a high coefficient for low values of used heritabilities and increases the variance of the test and then type 1 error rates. Since GRAMMAR is supposed to be a very conservative method, the difference observed between expected and obtained type 1 error rates may be surprising. The FASTA method behaved similarly but only a considerably underestimated heritability produced moderate increases in type 1 error rates. In this case, the true power (for true type 1 error rate) was reduced when heritability was underestimated (−4% when heritability was 0.10 instead of the true value of 0.30) but the decrease remained limited. Therefore, it appears that the fasta method works regardless of which estimate of heritability is used. When using the FASTA method, underestimating heritability was actually more risky (in terms of type 1 error rate and power) than overestimating it. However, it should be kept in mind that the power of even the true mixed model is lower for moderate heritabilities than for heritabilities of 0 or 1, regardless of the method used.

It should be noted that this discussion concerned only the first and second moments of the test statistics and did not compare higher moments such as skewness and kurtosis, which could also be of interest.

## Conclusions

Analytical formulae of the first and second moments of the distribution of the test statistics used to detect the SNP effect in four of the most common models are given in the case of structured populations due to relationships between individuals. These formulae were used to compute the type 1 error rate and power of these methods for any type of genetic relationships between phenotyped and genotyped individuals in any situation of heritability for a polygenic effect. The objective was to determine if these formulae can be easily used to obtain the correct type 1 error rate and to calculate the power in order to design data collection. An R program is provided in Additional file 3 [See Additional file 3]. This paper also gives general results concerning the efficacy of each method. The type 1 error rate increased with the variability of relationships among phenotyped and genotyped individuals and with heritability for the regression method, decreased for the GRAMMAR method and was not affected for the FASTA and QTDT methods. For the same true type 1 error rate, powers of the GRAMMAR and FASTA methods were the same but that of the QTDT method was low. In conclusion, we do not recommend the regression and GRAMMAR models but do recommend the FASTA method, which gives results very close to the full mixed model.

## Competing interests

The authors declare that they have no competing interests.

## Authors’ contributions

All authors were involved in the conception of the study. JME and AR wrote the manuscript. AR derived analytical results. ST performed simulations and wrote the R package. All authors read and approved the final manuscript.

## Supplementary Material

Additional file 1 **Details on the algebraic formulae used to obtain the results.** Details of matrix algebra used to construct the formulae in the main text. Click here for file

Additional file 2 **Simulations.** Details and standard errors of the simulations used to confirm the analytical results. Click here for file

Additional file 3 **RobPower.** This is the directory including Rpackage and documentation. This package computes all type 1 errors and the power of any kind of design with: 1. The A matrix obtained either from the pedigree file or from a known matrix. 2. The D matrix obtained either from the pedigree file or from a known matrix. 3. A value of heritability (can be a single value or a vector). 4. A threshold (for example the nominal 5% threshold, 0.05). 5. The phenotypic variance explained by the QTL (can be a single value or a vector). 6. The methods to compute (can be a single value or a vector of characters). Methods are “reg” for regression model, “GRAMMAR”, “QTDT” and “FASTA”. 7. Genomic control (GC). If GC = TRUE then the GC value is computed (available for “reg” and “GRAMMAR” only). 8. Linkage disequilibrium (r^2^) between the marker tested and the QTL. Click here for file

## References

[B1] Risch NJ Searching for genetic determinants in the new millennium Nature 2000 405 847 856 10.1038/35015718 10866211

[B2] Woolf B On estimating the relation between blood group and disease Ann Hum Genet 1955 19 251 253 10.1111/j.1469-1809.1955.tb01348.x 14388528

[B3] Grapes L Dekkers JCM Rothschild MF Fernando RL Comparing linkage disequilibrium-based methods for fine mapping quantitative trait loci Genetics 2004 166 1561 1570 10.1534/genetics.166.3.1561 15082569 PMC1470790

[B4] Zhao HH Fernando RL Dekkers JCM Power and precision of alternate methods for linkage disequilibrium mapping of quantitative trait loci Genetics 2007 175 1975 1986 10.1534/genetics.106.066480 17277369 PMC1855130

[B5] Meuwissen THE Goddard ME Fine mapping of quantitative trait loci using linkage disequilibria with closely linked marker loci Genetics 2000 155 421 430 10790414 10.1093/genetics/155.1.421 PMC1461086

[B6] Pritchard JK Rosenberg NA Use of unlinked genetic markers to detect population stratification in association studies Am J Hum Genet 1999 65 220 228 10.1086/302449 10364535 PMC1378093

[B7] Cardon LR Palmer LJ Population stratification and spurious allelic association Lancet 2003 361 598 604 10.1016/S0140-6736(03)12520-2 12598158

[B8] Marchini J Cardon LR Phillips MS Donnelly P The effects of human population structure on large genetic association studies Nature Genet 2004 36 512 517 10.1038/ng1337 15052271

[B9] Clayton DG Walker NM Smyth DJ Pask R Cooper JD Maier LM Smink LJ Lam AC Ovington NR Stevens HE Nutland S Howson JMM Faham M Moorhead M Jones HB Falkowski M Hardenbol P Willis TD Todd JA Population structure, differential bias and genomic control in a large-scale, case–control association study Nat Genet 2005 37 1243 1246 10.1038/ng1653 16228001

[B10] Spielman RS McGinnis RE Ewens WJ Transmission test for linkage disequilibrium: the insulin gene region and insulin-dependent diabetes mellitus (IDDM) Am J Hum Genet 1993 52 506 516 8447318 PMC1682161

[B11] Ewens WJ Spielman RS The transmission/disequilibrium test: history, subdivision, and admixture Am J Hum Genet 1995 57 455 464 10.1002/ajmg.1320570319 7668272 PMC1801556

[B12] Falk CT Rubinstein P Haplotype relative risks: an easy reliable way to construct a proper control sample for risk calculations Ann Hum Genet 1987 51 227 233 10.1111/j.1469-1809.1987.tb00875.x 3500674

[B13] Abecasis GR Cardon LR Cookson WO A general test of association for quantitative traits in nuclear families Am J Hum Genet 2000 66 279 292 10.1086/302698 10631157 PMC1288332

[B14] Abecasis GR Cookson WO Cardon LR Pedigree tests of transmission disequilibrium Eur J Hum Genet 2000 8 545 551 10.1038/sj.ejhg.5200494 10909856

[B15] Allison DB Transmission-disequilibrium tests for quantitative traits Am J Hum Genet 1997 60 676 690 9042929 PMC1712500

[B16] Fulker DW Cherny SS Sham PC Hewitt JK Combined linkage and association sib-pair analysis for quantitative traits Am J Hum Genet 1999 64 259 267 10.1086/302193 9915965 PMC1377724

[B17] Rabinowitz D A transmission disequilibrium test for quantitative trait loci Hum Hered 1997 47 342 350 10.1159/000154433 9391826

[B18] Laird NM Horvath S Xu X Implementing a unified approach to family-based tests of association Genet Epidemiol 2000 19 S36 S42 10.1002/1098-2272(2000)19:1+<::AID-GEPI6>3.0.CO;2-M 11055368

[B19] Laird NM Lange C Family-based designs in the age of large-scale gene-association studies Nat Rev Genet 2006 7 385 394 16619052 10.1038/nrg1839

[B20] Laird NM Lange C Family-based methods for linkage and association analysis Adv Genet 2008 60 219 252 18358323 10.1016/S0065-2660(07)00410-5

[B21] Lange C DeMeo DL Laird NM Power and design considerations for a general class of family-based association tests: quantitative traits Am J Hum Genet 2002 71 1330 1341 10.1086/344696 12454799 PMC378574

[B22] Ewens WJ Li M Spielman RS A review of family-based tests for linkage disequilibrium between a quantitative trait and a genetic marker PLoS Genet 2008 4 e1000180 10.1371/journal.pgen.1000180 18818728 PMC2528965

[B23] Balding DJ A tutorial on statistical methods for population association studies Nat Rev Genet 2006 7 781 791 10.1038/nrg1916 16983374

[B24] Devlin B Roeder K Genomic control for association studies Biometrics 1999 55 997 1004 10.1111/j.0006-341X.1999.00997.x 11315092

[B25] Bacanu SA Devlin B Roeder K Association studies for quantitative traits in structured populations Genet Epidemiol 2002 22 78 93 10.1002/gepi.1045 11754475

[B26] Price AL Patterson NJ Plenge RM Weinblatt ME Shadick NA Reich D Principal components analysis corrects for stratification in genome-wide association studies Nature Genet 2006 38 904 909 10.1038/ng1847 16862161

[B27] Pritchard JK Stephens M Donnelly P Inference of population structure using multilocus genotype data Genetics 2000 155 945 959 10835412 10.1093/genetics/155.2.945 PMC1461096

[B28] Pritchard JK Stephens M Rosenberg NA Donnelly P Association mapping in structured populations Am J Hum Genet 2000 67 170 181 10.1086/302959 10827107 PMC1287075

[B29] Satten GA Flanders WD Yang QH Accounting for unmeasured population substructure in case–control studies of genetic association using a novel latent-class model Am J Hum Genet 2001 68 466 477 10.1086/318195 11170894 PMC1235279

[B30] Zhu XF Li SC Cooper RS Elston RC A unified association analysis approach for family and unrelated samples correcting for stratification Am J Hum Genet 2008 82 352 365 10.1016/j.ajhg.2007.10.009 18252216 PMC2427300

[B31] Zhu XF Zhang SL Zhao HY Cooper RS Association mapping, using a mixture model for complex traits Genet Epidemiol 2002 23 181 196 10.1002/gepi.210 12214310

[B32] Meuwissen THE Karlsen A Lien S Olsaker I Goddard ME Fine mapping of a quantitative trait locus for twinning rate using combined linkage and linkage disequilibrium mapping Genetics 2002 161 373 379 12019251 10.1093/genetics/161.1.373 PMC1462098

[B33] Hayes BJ Chamberlain AJ McPartlan H Macleod I Sethuraman L Goddard ME Accuracy of marker-assisted selection with single markers and marker haplotypes in cattle Genet Res 2007 89 215 220 18208627 10.1017/S0016672307008865

[B34] Ritland K Estimators for pairwise relatedness and individual inbreeding coefficients Genet Res 1996 67 175 185 10.1017/S0016672300033620

[B35] VanRaden PM Efficient methods to compute genomic predictions J Dairy Sci 2008 91 4414 4423 10.3168/jds.2007-0980 18946147

[B36] Yang J Benyamin B McEvoy BP Gordon S Henders AK Nyholt DR Madden PA Heath AC Martin NG Montgomery GW Goddard ME Visscher PM Common SNPs explain a large proportion of the heritability for human height Nat Genet 2010 42 565 569 10.1038/ng.608 20562875 PMC3232052

[B37] Henderson CR Comparison of alternative sire evaluation methods J Anim Sci 1975 41 760 770

[B38] Quaas RL Pollak EJ Mixed model methodology for farm and ranch beef cattle testing programs J Anim Sci 1980 51 1277 1287

[B39] Price AL Zaitlen NA Reich D Patterson N New approaches to population stratification in genome-wide association studies Nat Rev Genet 2010 11 459 463 20548291 10.1038/nrg2813 PMC2975875

[B40] Zhang ZW Ersoz E Lai CQ Todhunter RJ Tiwari HK Gore MA Bradbury PJ Yu JM Arnett DK Ordovas JM Buckler ES Mixed linear model approach adapted for genome-wide association studies Nat Genet 2010 42 355 360 10.1038/ng.546 20208535 PMC2931336

[B41] Aulchenko YS de Koning DJ Haley C Genomewide rapid association using mixed model and regression: a fast and simple method for genomewide pedigree-based quantitative trait loci association analysis Genetics 2007 177 577 585 10.1534/genetics.107.075614 17660554 PMC2013682

[B42] Aulchenko YS Ripke S Isaacs A Van Duijn CM GenABEL: an R library for genome-wide association analysis Bioinformatics 2007 23 1294 1296 10.1093/bioinformatics/btm108 17384015

[B43] Amin N van Duijn CM Aulchenko YS A genomic background based method for association analysis in related individuals PLoS One 2007 2 e1274 10.1371/journal.pone.0001274 18060068 PMC2093991

[B44] Chen WM Abecasis GR Family-based association tests for genomewide association scans Am J Hum Genet 2007 81 913 926 10.1086/521580 17924335 PMC2265659

[B45] Kang HM Zaitlen NA Wade CM Kirby A Heckerman D Daly MJ Eskin E Efficient control of population structure in model organism association mapping Genetics 2008 178 1709 1723 10.1534/genetics.107.080101 18385116 PMC2278096

[B46] Kang HM Sul JH Service SK Zaitlen NA Kong SY Freimer NB Sabatti C Eskin E Variance component model to account for sample structure in genome-wide association studies Nat Genet 2010 42 348354 10.1038/ng.548 PMC3092069 20208533

[B47] Yu JM Pressoir G Briggs WH Vroh BI Yamasaki M Doebley JF McMullen MD Gaut BS Nielsen DM Holland JB Kresovich S Buckler ES A unified mixed-model method for association mapping that accounts for multiple levels of relatedness Nat Genet 2006 38 203 208 10.1038/ng1702 16380716

[B48] Zhang L Li J Pei YF Liu YJ Deng HW Tests of association for quantitative traits in nuclear families using principal components to correct for population stratification Ann Hum Genet 2009 73 601 613 10.1111/j.1469-1809.2009.00539.x 19702646 PMC2764806

[B49] Zhao KY Aranzana MJ Kim S Lister C Shindo C Tang CL Toomajian C Zheng HG Dean C Marjoram P Nordborg M An Arabidopsis example of association mapping in structured samples PLoS Genet 2007 3 e4 10.1371/journal.pgen.0030004 17238287 PMC1779303

[B50] Thornton T McPeek MS ROADTRIPS: Case–control association testing with partially or completely unknown population and pedigree structure Am J Hum Genet 2010 86 172 184 10.1016/j.ajhg.2010.01.001 20137780 PMC2820184

[B51] Zeggini E Scott LJ Saxena R Voight BF Marchini JL Hu T de Bakker PIW Abecasis GR Almgren P Andersen G Ardlie K Boström KB Bergman RN Bonnycastle LL Borch-Johnsen K Burtt NP Chen H Chines PS Daly MJ Deodhar P Ding CJ Doney AS Duren WL Elliott KS Erdos MR Frayling TM Freathy RM Gianniny L Grallert H Grarup N Meta-analysis of genome-wide association data and large-scale replication identifies additional susceptibility loci for type 2 diabetes Nature Genet 2008 40 638 645 10.1038/ng.120 18372903 PMC2672416

[B52] Lee AB Luca D Klei L Devlin B Roeder K Discovering genetic ancestry using spectral graph theory Genet Epidemiol 2010 34 51 59 19455578 10.1002/gepi.20434 PMC4610359

[B53] Wu CQ DeWan A Hoh J Wang ZH A comparison of association methods correcting for population stratification in case–control studies Ann Hum Genet 2011 75 418 427 10.1111/j.1469-1809.2010.00639.x 21281271 PMC3215268

[B54] Erbe M Ytournel F Pimentel ECG Sharifi AR Simianer H Power and robustness of three whole genome association mapping approaches in selected populations J Anim Breed Genet 2011 128 3 14 10.1111/j.1439-0388.2010.00885.x 21214639

[B55] Astle W Balding DJ Population structure and cryptic relatedness in genetic association studies Stat Sci 2009 24 451 471 10.1214/09-STS307

[B56] Fan RZ Xiong MM High resolution mapping of quantitative trait loci by linkage disequilibrium analysis Eur J Hum Genet 2002 10 607 615 10.1038/sj.ejhg.5200843 12357331

[B57] Freidlin B Zheng G Li ZH Gastwirth JL Trend tests for case–control studies of genetic markers: power, sample size and robustness Hum Hered 2002 53 146 152 10.1159/000064976 12145550

[B58] Guedj M Della-Chiesa E Picard F Nuel G Computing power in case–control association studies through the use of quadratic approximations: application to meta-statistics Ann Hum Genet 2007 71 262 270 10.1111/j.1469-1809.2006.00316.x 17032289

[B59] Li TF Li ZH Ying ZL Zhang H Influence of population stratification on population-based marker-disease association analysis Ann Hum Genet 2010 74 351 360 10.1111/j.1469-1809.2010.00588.x 20529080 PMC2897957

[B60] Ambrosius WT Lange EM Langefeld CD Power for genetic association studies with random allele frequencies and genotype distributions Am J Hum Genet 2004 74 683 693 10.1086/383282 15024689 PMC1181944

[B61] Kozlitina J Xing C Pertsemlidis A Schucany WR Power of genetic association studies with fixed and random genotype frequencies Ann Hum Genet 2010 74 429 438 10.1111/j.1469-1809.2010.00598.x 20645958

[B62] Boitard S Mangin B Azais JM Asymptotic distribution of the “orthogonal” quantitative transmission disequilibrium test in a structured population: exact formula Stat Appl Genet Mol Biol 2010 9 11 10.2202/1544-6115.1521 20196746

[B63] Johnson NL Kotz S Distributions in Statistics: Continuous Univariate Distributions 1970 New York: Wiley

[B64] Meuwissen THE Solberg TR Shepherd R Woolliams JA A fast algorithm for BayesB type of prediction of genome-wide estimates of genetic value Genet Sel Evol 2009 41 2 10.1186/1297-9686-41-2 19284681 PMC2637029

[B65] Kenward MG Roger JH Small sample inference for fixed effects from restricted maximum likelihood Biometrics 1997 53 983 997 10.2307/2533558 9333350

[B66] Gilmour AR, Gogel BJ, Cullis BR, Thompson R ASReml user guide release 3.0 2009 Hemel Hempstead: VSN International Ltd

[B67] Habier D Fernando RL Dekkers JCM The impact of genetic relationship information on genome-assisted breeding values Genetics 2007 177 2389 2397 18073436 10.1534/genetics.107.081190 PMC2219482

