# Supplementary material for: Statistical distributions of test statistics used for quantitative trait association mapping in structured populations
Source: Genet Sel Evol. 2012 Nov 12;44(1):32. doi: 10.1186/1297-9686-44-32 (PMC3817592; doi:10.1186/1297-9686-44-32)
Supplement: Additional file 1 — Details on the algebraic formulae used to obtain the results. Details of matrix algebra used to construct the formulae in the main text. [file 1297-9686-44-32-S1.docx]

**Additional file 1**

**Details on the algebraic formulae used in the main text**

# Model 1: Regression model

$E(\hat{\beta}^{(1)})=\mathbf{(x'x)}^{\mathbf{-}}\mathbf{x'}E(\mathbf{y})=\mathbf{(x'x)}^{\mathbf{-}}\mathbf{x'}(\mathbf{1}\mu+\mathbf{x}\beta)=\beta$ because $\mathbf{x'1}=(\mathbf{w}-\bar{\mathbf{w}})'\mathbf{1}=0$, thus the estimator of the SNP effect is still unbiased.

$$V(\hat{\beta}^{(1)})\mathbf{=(x'x)}^{\mathbf{-}}\mathbf{x'}V(\mathbf{y})\mathbf{x(x'x)}^{\mathbf{-}}=\mathbf{(x'x)}^{\mathbf{-}}\mathbf{x'}(\mathbf{A}\sigma_{u}^{2}+\mathbf{I}\sigma_{e}^{2})\mathbf{x(x'x)}^{\mathbf{-}}=\sigma_{e}^{2}\left[\mathbf{(x'x)}^{\mathbf{-}}+\frac{h^{2}}{1-h^{2}}\mathbf{(x'x)}^{\mathbf{-}}\mathbf{x'Ax(x'x)}^{\mathbf{-}}\right]$$

,

developing $E(\mathbf{y})'(\mathbf{I}-\mathbf{x(x'x)}^{\mathbf{-}}\mathbf{x'}-\mathbf{1}(\mathbf{1'1})^{-}\mathbf{1}')E(\mathbf{y})$:

$\begin{matrix} E(\mathbf{y})'(\mathbf{I}-\mathbf{x(x'x)}^{\mathbf{-}}\mathbf{x'}-\mathbf{1}(\mathbf{1'1})^{-}\mathbf{1}')E(\mathbf{y})=(\mu\mathbf{1}'+\beta\mathbf{x}')(\mathbf{I}-\mathbf{x(x'x)}^{\mathbf{-}}\mathbf{x'}-\mathbf{1}(\mathbf{1'1})^{-}\mathbf{1}')(\mathbf{1}\mu+\mathbf{x}\beta) \\ =(\mu\mathbf{1}'+\beta\mathbf{x}')(\mathbf{I}-\mathbf{x(x'x)}^{\mathbf{-}}\mathbf{x'}-\mathbf{1}(\mathbf{1'1})^{-}\mathbf{1}')(\mathbf{1}\mu+\mathbf{x}\beta-\mathbf{x}\beta-\mathbf{1}\mu) \\ =0 \end{matrix}$,

because $\mathbf{x'1}=(\mathbf{w}-\mathbf{1}\bar{w})'\mathbf{1}=0$.

Finally:

.

# Model 2: GRAMMAR model

In order to simplify notation, in this section, reference (2a) was replaced by (2).

The following equalities were used:

$$\left[\begin{matrix} \mathbf{C}_{11}^{(2)} & \mathbf{C}_{\mathbf{1u}}^{(2)} \\ \mathbf{C}_{\mathbf{u1}}^{(2)} & \mathbf{C}_{\mathbf{uu}}^{(2)} \end{matrix}\right]\left[\begin{matrix} \mathbf{1'1} & \mathbf{1'} \\ \mathbf{1} & \mathbf{I+\lambda}^{(2)}\mathbf{A}^{\mathbf{-1}} \end{matrix}\right]=\left[\begin{matrix} 1 & \mathbf{0'} \\ \mathbf{0} & \mathbf{I} \end{matrix}\right]$$

$$\left[\begin{matrix} \mathbf{C}_{11}^{(2)} & \mathbf{C}_{\mathbf{1u}}^{(2)} \\ \mathbf{C}_{\mathbf{u1}}^{(2)} & \mathbf{C}_{\mathbf{uu}}^{(2)} \end{matrix}\right]\left[\begin{matrix} \mathbf{1'1} & \mathbf{1'} \\ \mathbf{1} & \mathbf{I} \end{matrix}\right]=\left[\begin{matrix} 1 & \mathbf{0} \\ \mathbf{0} & \mathbf{I} \end{matrix}\right]-\left[\begin{matrix} 0 & \mathbf{\lambda}^{(2)}\mathbf{C}_{1\mathbf{u}}^{(2)}\mathbf{A}^{\mathbf{-1}} \\ \mathbf{0} & \mathbf{\lambda}^{(2)}\mathbf{C}_{\mathbf{uu}}^{(2)}\mathbf{A}^{\mathbf{-1}} \end{matrix}\right]$$

So :

$\mathbf{C}_{\mathbf{u1}}^{(2)}\mathbf{1'}+\mathbf{C}_{\mathbf{uu}}^{(2)}=\mathbf{I}-\mathbf{\lambda}^{(2)}\mathbf{C}_{\mathbf{uu}}^{(2)}\mathbf{A}^{\mathbf{-1}}$ ou $\mathbf{1C}_{\mathbf{1u}}^{(2)}+\mathbf{C}_{\mathbf{uu}}^{(2)}=\mathbf{I}-\mathbf{\lambda}^{(2)}\mathbf{A}^{\mathbf{-1}}\mathbf{C}_{\mathbf{uu}}^{(2)}$

$\mathbf{C}_{\mathbf{u1}}^{(2)}=-\frac{1}{n}\mathbf{C}_{\mathbf{uu}}^{(2)}\mathbf{1}$ or $\mathbf{C}_{\mathbf{u1}}^{(2)}\mathbf{1'1}+\mathbf{C}_{\mathbf{uu}}^{(2)}\mathbf{1}=0$

$\mathbf{C}_{1\mathbf{1}}^{(2)}\mathbf{1'}+\mathbf{C}_{1\mathbf{u}}^{(2)}=-\mathbf{C}_{1\mathbf{u}}^{(2)}\mathbf{\lambda}^{(2)}\mathbf{A}^{\mathbf{-1}}$ or $\mathbf{1C}_{1\mathbf{1}}^{(2)}+\mathbf{C}_{u1}^{(2)}=-\mathbf{A}^{\mathbf{-1}}\mathbf{C}_{\mathbf{u}1}^{(2)}\mathbf{\lambda}^{(2)}$

$\mathbf{C}_{11}^{(2)}\mathbf{1'1}+\mathbf{C}_{\mathbf{1u}}^{(2)}\mathbf{1}=1$ or $\mathbf{1'1C}_{11}^{(2)}+\mathbf{1'C}_{\mathbf{u1}}^{(2)}=1$.

## calculation of $V(\hat{\mathbf{u}}^{(2)})$

To express $V(\hat{\mathbf{u}}^{(2)})$, it was necessary to separate the classical expression $(\mathbf{A}\sigma_{u}^{2}-\mathbf{C}_{\mathbf{uu}}^{(2)}\mathbf{\lambda}^{(2)}\sigma_{u}^{2})$from the part corresponding to the difference between variance components of the true model and variance components of the pure random model (2a): $(\sigma_{e}^{2}-\lambda^{(2)}\sigma_{u}^{2})\mathbf{C}_{\mathbf{uu}}^{(2)}(\mathbf{I}-\lambda^{(2)}\mathbf{A}^{-1}\mathbf{C}_{\mathbf{uu}}^{(2)})$ (if $\lambda^{(2)}=\frac{\sigma_{e}^{2}}{\sigma_{u}^{2}}$, instead of $\lambda^{(2)}=\frac{\sigma_{e^{(2a)}}^{2}}{\sigma_{u^{(2a)}}^{2}}$this last term disappears)

$$\begin{matrix} V(\hat{\mathbf{u}}^{(2)})=(\mathbf{C}_{\mathbf{u1}}^{(2)}\mathbf{1}'+\mathbf{C}_{\mathbf{uu}}^{(2)})V(\mathbf{y})(\mathbf{C}_{\mathbf{u1}}^{(2)}\mathbf{1}'+\mathbf{C}_{\mathbf{uu}}^{(2)})' \\ =(\mathbf{C}_{\mathbf{u1}}^{(2)}\mathbf{1}'+\mathbf{C}_{\mathbf{uu}}^{(2)})(\mathbf{A}\sigma_{u}^{2}+\mathbf{I}\sigma_{e}^{2})(\mathbf{C}_{\mathbf{u1}}^{(2)}\mathbf{1}'+\mathbf{C}_{\mathbf{uu}}^{(2)})' \end{matrix}$$

$$=(\mathbf{I}-\mathbf{C}_{\mathbf{uu}}^{(2)}\mathbf{\lambda}^{(2)}\mathbf{A}^{\mathbf{-1}})\mathbf{A}\sigma_{u}^{2}(\mathbf{C}_{\mathbf{u1}}^{(2)}\mathbf{1}'+\mathbf{C}_{\mathbf{uu}}^{(2)})'+\sigma_{e}^{2}(\mathbf{C}_{\mathbf{u1}}^{(2)}\mathbf{1}'+\mathbf{C}_{\mathbf{uu}}^{(2)})(\mathbf{C}_{\mathbf{u1}}^{(2)}\mathbf{1}'+\mathbf{C}_{\mathbf{uu}}^{(2)})'$$

(in the left part, we used $\mathbf{C}_{\mathbf{u1}}^{(2)}\mathbf{1'}+\mathbf{C}_{\mathbf{uu}}^{(2)}=\mathbf{I}-\mathbf{C}_{\mathbf{uu}}^{(2)}\mathbf{\lambda}^{(2)}\mathbf{A}^{\mathbf{-1}}$)

$$\begin{matrix} =\sigma_{u}^{2}\mathbf{A}(\mathbf{C}_{\mathbf{u1}}^{(2)}\mathbf{1}'+\mathbf{C}_{\mathbf{uu}}^{(2)})'-\sigma_{u}^{2}\mathbf{C}_{\mathbf{uu}}^{(2)}\mathbf{\lambda}^{(2)}(\mathbf{C}_{\mathbf{u1}}^{(2)}\mathbf{1}'+\mathbf{C}_{\mathbf{uu}}^{(2)})'+\sigma_{e}^{2}(\mathbf{C}_{\mathbf{u1}}^{(2)}\mathbf{1}'+\mathbf{C}_{\mathbf{uu}}^{(2)})(\mathbf{C}_{\mathbf{u1}}^{(2)}\mathbf{1}'+\mathbf{C}_{\mathbf{uu}}^{(2)})' \\ =\sigma_{u}^{2}\mathbf{A}(\mathbf{I}-\mathbf{A}^{\mathbf{-1}}\mathbf{C}_{\mathbf{uu}}^{(2)}\mathbf{\lambda}^{(2)})+(\sigma_{e}^{2}(\mathbf{C}_{\mathbf{u1}}^{(2)}\mathbf{1}'+\mathbf{C}_{\mathbf{uu}}^{(2)})-\sigma_{u}^{2}\mathbf{C}_{\mathbf{uu}}^{(2)}\mathbf{\lambda}^{(2)})(\mathbf{C}_{\mathbf{u1}}^{(2)}\mathbf{1}'+\mathbf{C}_{\mathbf{uu}}^{(2)})' \\ =(\mathbf{A}\sigma_{u}^{2}-\mathbf{C}_{\mathbf{uu}}^{(2)}\mathbf{\lambda}^{(2)}\sigma_{u}^{2})+(\sigma_{e}^{2}\mathbf{C}_{\mathbf{uu}}^{(2)}-\sigma_{u}^{2}\mathbf{C}_{\mathbf{uu}}^{(2)}\mathbf{\lambda}^{(2)})(\mathbf{C}_{\mathbf{u1}}^{(2)}\mathbf{1}'+\mathbf{C}_{\mathbf{uu}}^{(2)})' \end{matrix}$$

(because $\mathbf{C}_{\mathbf{u1}}^{(2)}\mathbf{1}'\mathbf{1C}_{\mathbf{1u}}^{(2)}+\mathbf{C}_{\mathbf{u1}}^{(2)}\mathbf{1}'\mathbf{C}_{\mathbf{uu}}^{(2)}=0$).

Thus:

$V(\hat{\mathbf{u}}^{(2)})=\sigma_{u}^{2}(\mathbf{A}-\lambda^{(2)}\mathbf{C}_{\mathbf{uu}}^{(2)})+(\sigma_{e}^{2}-\lambda^{(2)}\sigma_{u}^{2})\mathbf{C}_{\mathbf{uu}}^{(2)}(\mathbf{I}-\lambda^{(2)}\mathbf{A}^{-1}\mathbf{C}_{\mathbf{uu}}^{(2)})$.

## calculation of $E(\hat{\mathbf{u}}^{(2)})$

$$E(\hat{\mathbf{u}}^{(2)})=(\mathbf{C}_{u1}\mathbf{1}'+\mathbf{C}_{uu})E(\mathbf{y})=(\mathbf{C}_{u1}\mathbf{1}'+\mathbf{C}_{uu})(\mathbf{1}\mu+\mathbf{x}\beta)=(\mathbf{C}_{u1}\mathbf{1}'+\mathbf{C}_{uu})\mathbf{x}\beta=\mathbf{C}_{uu}\mathbf{x}\beta$$

(because $\mathbf{C}_{\mathbf{u1}}^{(2)}\mathbf{1'1}+\mathbf{C}_{\mathbf{uu}}^{(2)}\mathbf{1}=0$et $\mathbf{x'1}=0$).

## Calculation of$E(\hat{\beta}^{(2b)})$

$$\begin{matrix} E(\hat{\beta}^{(2b)})=\mathbf{(x'x)}^{\mathbf{-}}\mathbf{x'}E(\mathbf{y}-\mathbf{1}\hat{\mu}^{(2a)}-\hat{\mathbf{u}}^{(2a)}) \\ =\mathbf{(x'x)}^{\mathbf{-}}\mathbf{x'}E(\mathbf{y})-\mathbf{(x'x)}^{\mathbf{-}}\mathbf{x'}E(\hat{\mathbf{u}}^{(2a)}) \end{matrix}$$

(because $\mathbf{x'1}=(\mathbf{w}-\bar{\mathbf{w}})'\mathbf{1}=0$)

$\begin{matrix} E(\hat{\beta}^{(2b)})=\mathbf{(x'x)}^{\mathbf{-}}\mathbf{x'x}\beta-\mathbf{(x'x)}^{\mathbf{-}}\mathbf{x'C}_{uu}\mathbf{x}\beta\\ =\beta-\mathbf{(x'x)}^{\mathbf{-}}\mathbf{x'C}_{uu}\mathbf{x}\beta\end{matrix}$.

## Calculation of$V(\hat{\beta}^{(2b)})$

Replacing $\hat{\mu}^{(2)}$and $\hat{\mathbf{u}}^{(2)}$by their expression in the mixed model equations:

$$\begin{matrix} V(\hat{\beta}^{(2b)})=\mathbf{(x'x)}^{\mathbf{-}}\mathbf{x'}V(\mathbf{y}-\mathbf{1}\hat{\mu}^{(2)}-\hat{\mathbf{u}}^{(2)})\mathbf{x(x'x)}^{\mathbf{-}} \\ =\mathbf{(x'x)}^{\mathbf{-}}\mathbf{x'}V((\mathbf{I}-\mathbf{1}\left[\begin{matrix} \mathbf{C}_{11}^{(2)} & \mathbf{C}_{\mathbf{1u}}^{(2)} \end{matrix}\right]\left[\begin{matrix} \mathbf{1'} \\ \mathbf{I} \end{matrix}\right]-\left[\begin{matrix} \mathbf{C}_{\mathbf{u1}}^{(2)} & \mathbf{C}_{\mathbf{uu}}^{(2)} \end{matrix}\right]\left[\begin{matrix} \mathbf{1'} \\ \mathbf{I} \end{matrix}\right])\mathbf{y})\mathbf{x(x'x)}^{\mathbf{-}} \end{matrix}$$

$V(\hat{\beta}^{(2b)})=\mathbf{(x'x)}^{\mathbf{-}}\mathbf{x'}V((\mathbf{I}-\left[\begin{matrix} \mathbf{C}_{\mathbf{u1}}^{(2)} & \mathbf{C}_{\mathbf{uu}}^{(2)} \end{matrix}\right]\left[\begin{matrix} \mathbf{1'} \\ \mathbf{I} \end{matrix}\right])\mathbf{y})\mathbf{x(x'x)}^{\mathbf{-}}$,

because $\mathbf{x'1}=0$

$\begin{matrix} V(\hat{\beta}^{(2b)})= \\ \mathbf{(x'x)}^{\mathbf{-}}\mathbf{x'}(\mathbf{I}-\left[\begin{matrix} \mathbf{C}_{\mathbf{u1}}^{(2)} & \mathbf{C}_{\mathbf{uu}}^{(2)} \end{matrix}\right]\left[\begin{matrix} \mathbf{1'} \\ \mathbf{I} \end{matrix}\right])\mathbf{A}\sigma_{u}^{2}(\mathbf{I}-\left[\begin{matrix} \mathbf{1} & \mathbf{I} \end{matrix}\right]\left[\begin{matrix} \mathbf{C}_{\mathbf{1u}}^{(2)} \\ \mathbf{C}_{\mathbf{uu}}^{(2)} \end{matrix}\right])+\sigma_{e}^{2}(\mathbf{I}-\left[\begin{matrix} \mathbf{C}_{\mathbf{u1}}^{(2)} & \mathbf{C}_{\mathbf{uu}}^{(2)} \end{matrix}\right]\left[\begin{matrix} \mathbf{1'} \\ \mathbf{I} \end{matrix}\right])(\mathbf{I}-\left[\begin{matrix} \mathbf{1} & \mathbf{I} \end{matrix}\right]\left[\begin{matrix} \mathbf{C}_{\mathbf{1u}}^{(2)} \\ \mathbf{C}_{\mathbf{uu}}^{(2)} \end{matrix}\right]))\mathbf{x(x'x)}^{\mathbf{-}} \\ =\mathbf{(x'x)}^{\mathbf{-}}\mathbf{x'}((\mathbf{C}_{\mathbf{uu}}^{(2)}\mathbf{\lambda}^{(2)}\mathbf{A}^{\mathbf{-1}})\mathbf{A}\sigma_{u}^{2}(\mathbf{A}^{\mathbf{-1}}\mathbf{C}_{\mathbf{uu}}^{(2)}\mathbf{\lambda}^{(2)}) \\ +\sigma_{e}^{2}(\mathbf{I}-\mathbf{C}_{\mathbf{u1}}^{(2)}\mathbf{1'}-\mathbf{C}_{\mathbf{uu}}^{(2)}-\mathbf{1C}_{\mathbf{1u}}^{(2)}+\mathbf{C}_{\mathbf{u1}}^{(2)}\mathbf{1}'\mathbf{1C}_{\mathbf{1u}}^{(2)}+\mathbf{C}_{\mathbf{uu}}^{(2)}\mathbf{1C}_{\mathbf{1u}}^{(2)}-\mathbf{C}_{\mathbf{uu}}^{(2)}+\mathbf{C}_{\mathbf{u1}}^{(2)}\mathbf{1}'\mathbf{C}_{\mathbf{uu}}^{(2)}+\mathbf{C}_{\mathbf{uu}}^{(2)}\mathbf{C}_{\mathbf{uu}}^{(2)}))\mathbf{x(x'x)}^{\mathbf{-}} \end{matrix}$because $\mathbf{C}_{\mathbf{uu}}^{(2)}\mathbf{\lambda}^{(2)}\mathbf{A}^{\mathbf{-1}}=\mathbf{I}-\mathbf{C}_{\mathbf{u1}}^{(2)}\mathbf{1'}-\mathbf{C}_{\mathbf{uu}}^{(2)}$

$=\mathbf{(x'x)}^{\mathbf{-}}\mathbf{x'}(\mathbf{\lambda}^{(2)}\mathbf{\lambda}^{(2)}\sigma_{u}^{2}(\mathbf{C}_{\mathbf{uu}}^{(2)}\mathbf{A}^{\mathbf{-1}}\mathbf{C}_{\mathbf{uu}}^{(2)})+\sigma_{e}^{2}(\mathbf{I}-2\mathbf{C}_{\mathbf{uu}}^{(2)}+\mathbf{C}_{\mathbf{u1}}^{(2)}\mathbf{1}'\mathbf{1C}_{\mathbf{1u}}^{(2)}+\mathbf{C}_{\mathbf{uu}}^{(2)}\mathbf{1C}_{\mathbf{1u}}^{(2)}+\mathbf{C}_{\mathbf{u1}}^{(2)}\mathbf{1}'\mathbf{C}_{\mathbf{uu}}^{(2)}+\mathbf{C}_{\mathbf{uu}}^{(2)}\mathbf{C}_{\mathbf{uu}}^{(2)}))\mathbf{x(x'x)}^{\mathbf{-}}$because$\mathbf{x'1}=0$

$$=\mathbf{(x'x)}^{\mathbf{-}}\mathbf{x'}(\mathbf{\lambda}^{(2)}\mathbf{\lambda}^{(2)}\sigma_{u}^{2}(\mathbf{C}_{\mathbf{uu}}^{(2)}\mathbf{A}^{\mathbf{-1}}\mathbf{C}_{\mathbf{uu}}^{(2)})+\sigma_{e}^{2}(\mathbf{I}-2\mathbf{C}_{\mathbf{uu}}^{(2)}+\mathbf{C}_{\mathbf{u1}}^{(2)}\mathbf{1}'\mathbf{C}_{\mathbf{uu}}^{(2)}+\mathbf{C}_{\mathbf{uu}}^{(2)}\mathbf{C}_{\mathbf{uu}}^{(2)}))\mathbf{x(x'x)}^{\mathbf{-}}$$

because $\mathbf{C}_{\mathbf{u1}}^{(2)}\mathbf{1}'\mathbf{1}+\mathbf{C}_{\mathbf{uu}}^{(2)}\mathbf{1}=0$

$$=\mathbf{(x'x)}^{\mathbf{-}}\mathbf{x'}(\mathbf{\lambda}^{(2)}\mathbf{\lambda}^{(2)}\sigma_{u}^{2}(\mathbf{C}_{\mathbf{uu}}^{(2)}\mathbf{A}^{\mathbf{-1}}\mathbf{C}_{\mathbf{uu}}^{(2)})+\sigma_{e}^{2}(\mathbf{I}-2\mathbf{C}_{\mathbf{uu}}^{(2)}+(\mathbf{I}-\mathbf{\lambda}^{(2)}\mathbf{C}_{\mathbf{uu}}^{(2)}\mathbf{A}^{\mathbf{-1}})\mathbf{C}_{\mathbf{uu}}^{(2)}))\mathbf{x(x'x)}^{\mathbf{-}}$$

because $\mathbf{C}_{\mathbf{u1}}^{(2)}\mathbf{1'}+\mathbf{C}_{\mathbf{uu}}^{(2)}=\mathbf{I}-\mathbf{C}_{\mathbf{uu}}^{(2)}\mathbf{\lambda}^{(2)}\mathbf{A}^{\mathbf{-1}}$

$\begin{matrix} =\mathbf{(x'x)}^{\mathbf{-}}\mathbf{x'}(\mathbf{\lambda}^{(2)}\mathbf{\lambda}^{(2)}\sigma_{u}^{2}(\mathbf{C}_{\mathbf{uu}}^{(2)}\mathbf{A}^{\mathbf{-1}}\mathbf{C}_{\mathbf{uu}}^{(2)})+\sigma_{e}^{2}(\mathbf{I}-\mathbf{C}_{\mathbf{uu}}^{(2)}-\mathbf{\lambda}^{(2)}\mathbf{C}_{\mathbf{uu}}^{(2)}\mathbf{A}^{\mathbf{-1}}\mathbf{C}_{\mathbf{uu}}^{(2)}))\mathbf{x(x'x)}^{\mathbf{-}} \\ =\sigma_{e}^{2}\left(\mathbf{(x'x)}^{\mathbf{-}}-\mathbf{(x'x)}^{\mathbf{-}}\mathbf{x'C}_{\mathbf{uu}}^{(2)}\mathbf{x(x'x)}^{\mathbf{-}}\right)-(\sigma_{e}^{2}-\mathbf{\lambda}^{(2)}\sigma_{u}^{2})\mathbf{\lambda}^{(2)}\mathbf{(x'x)}^{\mathbf{-}}\mathbf{x'C}_{\mathbf{uu}}^{(2)}\mathbf{A}^{\mathbf{-1}}\mathbf{C}_{\mathbf{uu}}^{(2)}\mathbf{x(x'x)}^{\mathbf{-}} \end{matrix}$.

If this formula is expressed only with $\mathbf{A}$ and $\mathbf{C}_{\mathbf{uu}}^{(2)}$and not with $\mathbf{A}^{-1}$to directly evaluate the effect of relationships and since we know that:

$\mathbf{C}_{\mathbf{u1}}^{(2)}\mathbf{1'}+\mathbf{C}_{\mathbf{uu}}^{(2)}=\mathbf{I}-\mathbf{C}_{\mathbf{uu}}^{(2)}\mathbf{\lambda}^{(2)}\mathbf{A}^{\mathbf{-1}}$and $\mathbf{C}_{\mathbf{u1}}^{(2)}=-\frac{1}{n}\mathbf{C}_{\mathbf{uu}}^{(2)}\mathbf{1}$, so $\mathbf{C}_{\mathbf{uu}}^{(2)}\mathbf{\lambda}^{(2)}\mathbf{A}^{\mathbf{-1}}=\mathbf{I}-\mathbf{C}_{\mathbf{uu}}^{(2)}\left(\mathbf{I}-\frac{1}{n}\mathbf{11'}\right)$,Then$V(\hat{\beta}^{(2b)})=\sigma_{e}^{2}\left(\mathbf{(x'x)}^{\mathbf{-}}-\mathbf{(x'x)}^{\mathbf{-}}\mathbf{x'C}_{\mathbf{uu}}^{(2)}\mathbf{x(x'x)}^{\mathbf{-}}\right)-(\sigma_{e}^{2}-\mathbf{\lambda}^{(2)}\sigma_{u}^{2})\mathbf{(x'x)}^{\mathbf{-}}\mathbf{x'}\left(\mathbf{C}_{\mathbf{uu}}^{(2)}-\mathbf{C}_{\mathbf{uu}}^{(2)}\left(\mathbf{I}-\frac{1}{n}\mathbf{11'}\right)\mathbf{C}_{\mathbf{uu}}^{(2)}\right)\mathbf{x(x'x)}^{\mathbf{-}}$ $=\sigma_{e}^{2}\left(\mathbf{(x'x)}^{\mathbf{-}}-\mathbf{(x'x)}^{\mathbf{-}}\mathbf{x'C}_{\mathbf{uu}}^{(2)}\mathbf{x(x'x)}^{\mathbf{-}}\right)-(\sigma_{e}^{2}-\mathbf{\lambda}^{(2)}\sigma_{u}^{2})\mathbf{(x'x)}^{\mathbf{-}}\left(\mathbf{x'C}_{\mathbf{uu}}^{(2)}\mathbf{x}-\mathbf{x'C}_{\mathbf{uu}}^{(2)}\mathbf{C}_{\mathbf{uu}}^{(2)}\mathbf{x}+\frac{1}{n}\mathbf{x'C}_{\mathbf{uu}}^{(2)}\mathbf{11'C}_{\mathbf{uu}}^{(2)}\mathbf{x}\right)\mathbf{(x'x)}^{\mathbf{-}}$.

The term $\mathbf{x'C}_{\mathbf{uu}}^{(2)}\mathbf{11'C}_{\mathbf{uu}}^{(2)}\mathbf{x}$ was zero when families had equal sizes because:

$\begin{matrix} E_{x}(\mathbf{x}'\mathbf{C}_{\mathbf{uu}}^{(2)}\mathbf{11}'\mathbf{C}_{\mathbf{uu}}^{(2)}\mathbf{x})=\mathbf{1}'\mathbf{A1}-\lambda^{(2)}\mathbf{1}'\mathbf{C}_{\mathbf{uu}}^{(2)}\mathbf{1} \\ =\mathbf{1}'(\mathbf{A}-\lambda^{(2)}\mathbf{C}_{\mathbf{uu}}^{(2)})\mathbf{1} \\ =\mathbf{1}'(\mathbf{C}_{\mathbf{u1}}^{(2)}\mathbf{1'A}+\mathbf{C}_{\mathbf{uu}}^{(2)}\mathbf{A})\mathbf{1} \\ =\mathbf{1}'(-\frac{1}{n}\mathbf{C}_{\mathbf{uu}}^{(2)}\mathbf{11'A}+\mathbf{C}_{\mathbf{uu}}^{(2)}\mathbf{A})\mathbf{1} \\ =-\frac{1}{n}\mathbf{1}'\mathbf{C}_{\mathbf{uu}}^{(2)}\mathbf{11'A1}+\mathbf{1}'\mathbf{C}_{\mathbf{uu}}^{(2)}\mathbf{A1} \end{matrix}$,

which is the covariance between the sum of column of $\mathbf{A}$ and $\mathbf{C}_{\mathbf{uu}}^{(2)}$, equal to zero when the design has equal family type (half sibs or full sibs…) and size because there was no variance between columns.

## Calculation of

$$\begin{matrix} \hat{\mathbf{e}}^{(2b)}=\hat{\mathbf{e}}^{(2)}-\mathbf{1}\hat{\mu}^{(2b)}-\mathbf{x}\hat{\beta}^{(2b)} \\ =\left(\mathbf{I}-\left[\begin{matrix} \mathbf{1} & \mathbf{x} \end{matrix}\right]\left[\begin{matrix} \mathbf{1'1} & 0 \\ 0 & \mathbf{x'x} \end{matrix}\right]^{-}\left[\begin{matrix} \mathbf{1'} \\ \mathbf{x'} \end{matrix}\right]\right)\hat{\mathbf{e}}^{(2)} \\ =(\mathbf{I}-\mathbf{x(x'x)}^{\mathbf{-}}\mathbf{x'}-\mathbf{1}(\mathbf{1'1})^{-}\mathbf{1}')\hat{\mathbf{e}}^{(2)} \end{matrix}$$

because $\mathbf{x'1}=0$

.

## In the part with $V(\hat{\mathbf{e}}^{(2)})$

We had:

$\begin{matrix} V(\hat{\mathbf{e}}^{(2)})=V(\left(\mathbf{I}-\mathbf{1}\left[\begin{matrix} \mathbf{C}_{11}^{(2)} & \mathbf{C}_{\mathbf{1u}}^{(2)} \end{matrix}\right]\left[\begin{matrix} \mathbf{1'} \\ \mathbf{I} \end{matrix}\right]-\left[\begin{matrix} \mathbf{C}_{\mathbf{u1}}^{(2)} & \mathbf{C}_{\mathbf{uu}}^{(2)} \end{matrix}\right]\left[\begin{matrix} \mathbf{1'} \\ \mathbf{I} \end{matrix}\right]\right)\mathbf{y}) \\ =\left(\mathbf{I}-\mathbf{1}\left[\begin{matrix} \mathbf{C}_{11}^{(2)} & \mathbf{C}_{\mathbf{1u}}^{(2)} \end{matrix}\right]\left[\begin{matrix} \mathbf{1'} \\ \mathbf{I} \end{matrix}\right]-\left[\begin{matrix} \mathbf{C}_{\mathbf{u1}}^{(2)} & \mathbf{C}_{\mathbf{uu}}^{(2)} \end{matrix}\right]\left[\begin{matrix} \mathbf{1'} \\ \mathbf{I} \end{matrix}\right]\right)V(\mathbf{y})\left(\mathbf{I}-\left[\begin{matrix} \mathbf{1} & \mathbf{I} \end{matrix}\right]\left[\begin{matrix} \mathbf{C}_{11}^{(2)} \\ \mathbf{C}_{\mathbf{u1}}^{(2)} \end{matrix}\right]\mathbf{1}'-\left[\begin{matrix} \mathbf{1} & \mathbf{I} \end{matrix}\right]\left[\begin{matrix} \mathbf{C}_{\mathbf{1u}}^{(2)} \\ \mathbf{C}_{\mathbf{uu}}^{(2)} \end{matrix}\right]\right) \end{matrix}$.

So that the trace was:

$\begin{matrix} tr((\mathbf{I}-\mathbf{x(x'x)}^{\mathbf{-}}\mathbf{x'}-\mathbf{1}(\mathbf{1'1})^{-}\mathbf{1}')V(\hat{\mathbf{e}}^{(2)})) \\ =tr((\mathbf{I}-\mathbf{1}(\mathbf{1'1})^{-}\mathbf{1}')\left(\mathbf{I}-\mathbf{1}\left[\begin{matrix} \mathbf{C}_{11}^{(2)} & \mathbf{C}_{\mathbf{1u}}^{(2)} \end{matrix}\right]\left[\begin{matrix} \mathbf{1'} \\ \mathbf{I} \end{matrix}\right]-\left[\begin{matrix} \mathbf{C}_{\mathbf{u1}}^{(2)} & \mathbf{C}_{\mathbf{uu}}^{(2)} \end{matrix}\right]\left[\begin{matrix} \mathbf{1'} \\ \mathbf{I} \end{matrix}\right]\right)V(\mathbf{y})\left(\mathbf{I}-\left[\begin{matrix} \mathbf{1} & \mathbf{I} \end{matrix}\right]\left[\begin{matrix} \mathbf{C}_{11}^{(2)} \\ \mathbf{C}_{\mathbf{u1}}^{(2)} \end{matrix}\right]\mathbf{1}'-\left[\begin{matrix} \mathbf{1} & \mathbf{I} \end{matrix}\right]\left[\begin{matrix} \mathbf{C}_{\mathbf{1u}}^{(2)} \\ \mathbf{C}_{\mathbf{uu}}^{(2)} \end{matrix}\right]\right)) \\ -tr(\mathbf{(x'x)}^{\mathbf{-}}\mathbf{x'}V(\hat{\mathbf{e}}^{(2)})\mathbf{x}) \end{matrix}$In the first term, all the terms which began by $\mathbf{1}$or ended by $\mathbf{1}'$ were suppressed because$tr((\mathbf{I}-\mathbf{1}(\mathbf{1'1})^{-}\mathbf{1}')\mathbf{B})=0\text{ if }\mathbf{B}=\mathbf{1G1'}or\text{ }\mathbf{B}=\mathbf{1G}\text{ }or\text{ }\mathbf{B}=\mathbf{G1}'$. The second term was used in the calculation of $V(\hat{\beta}^{(2b)})$and we used the same developments:

$\begin{matrix} =tr((\mathbf{I}-\mathbf{1}(\mathbf{1'1})^{-}\mathbf{1}')\left(\mathbf{I}-\left[\begin{matrix} \mathbf{C}_{\mathbf{u1}}^{(2)} & \mathbf{C}_{\mathbf{uu}}^{(2)} \end{matrix}\right]\left[\begin{matrix} \mathbf{1'} \\ \mathbf{I} \end{matrix}\right]\right)V(\mathbf{y})\left(\mathbf{I}-\left[\begin{matrix} \mathbf{1} & \mathbf{I} \end{matrix}\right]\left[\begin{matrix} \mathbf{C}_{\mathbf{1u}}^{(2)} \\ \mathbf{C}_{\mathbf{uu}}^{(2)} \end{matrix}\right]\right)) \\ -tr(\sigma_{e}^{2}\left(1-\mathbf{(x'x)}^{\mathbf{-}}\mathbf{x'C}_{\mathbf{uu}}^{(2)}\mathbf{x}\right)-(\sigma_{e}^{2}-\mathbf{\lambda}^{(2)}\sigma_{u}^{2})\mathbf{\lambda}^{(2)}\mathbf{(x'x)}^{\mathbf{-}}\mathbf{x'C}_{\mathbf{uu}}^{(2)}\mathbf{A}^{\mathbf{-1}}\mathbf{C}_{\mathbf{uu}}^{(2)}\mathbf{x}) \end{matrix}$.

In the first term, we used $V(\mathbf{y})=\mathbf{A}\sigma_{u}^{2}+\mathbf{I}\sigma_{u}^{2}$ and $\mathbf{C}_{\mathbf{u1}}^{(2)}\mathbf{1'}+\mathbf{C}_{\mathbf{uu}}^{(2)}=\mathbf{I}-\mathbf{C}_{\mathbf{uu}}^{(2)}\mathbf{\lambda}^{(2)}\mathbf{A}^{\mathbf{-1}}$. For the second term, the trace was useless because it was a scalar.

$$\begin{matrix} =tr((\mathbf{I}-\mathbf{1}(\mathbf{1'1})^{-}\mathbf{1}')(\mathbf{C}_{\mathbf{uu}}^{(2)}\mathbf{\lambda}^{(2)}\mathbf{A}^{\mathbf{-1}}\mathbf{AA}^{\mathbf{-1}}\mathbf{\lambda}^{(2)}\mathbf{C}_{\mathbf{uu}}^{(2)}\sigma_{u}^{2}+\sigma_{e}^{2}(\mathbf{I}-2\mathbf{C}_{\mathbf{uu}}^{(2)}+(\mathbf{I}-\mathbf{C}_{\mathbf{uu}}^{(2)}\mathbf{\lambda}^{(2)}\mathbf{A}^{\mathbf{-1}})\mathbf{C}_{\mathbf{uu}}^{(2)})) \\ -\sigma_{e}^{2}\left(1-\mathbf{(x'x)}^{\mathbf{-}}\mathbf{x'C}_{\mathbf{uu}}^{(2)}\mathbf{x}\right)+(\sigma_{e}^{2}-\mathbf{\lambda}^{(2)}\sigma_{u}^{2})\mathbf{\lambda}^{(2)}\mathbf{(x'x)}^{\mathbf{-}}\mathbf{x'C}_{\mathbf{uu}}^{(2)}\mathbf{A}^{\mathbf{-1}}\mathbf{C}_{\mathbf{uu}}^{(2)}\mathbf{x} \end{matrix}$$

$$\begin{matrix} =tr((\mathbf{I}-\mathbf{1}(\mathbf{1'1})^{-}\mathbf{1}')(\mathbf{C}_{\mathbf{uu}}^{(2)}\mathbf{A}^{\mathbf{-1}}\mathbf{C}_{\mathbf{uu}}^{(2)}\mathbf{\lambda}^{(2)}\mathbf{\lambda}^{(2)}\sigma_{u}^{2}+\sigma_{e}^{2}(\mathbf{I}-\mathbf{C}_{\mathbf{uu}}^{(2)}-\mathbf{C}_{\mathbf{uu}}^{(2)}\mathbf{\lambda}^{(2)}\mathbf{A}^{\mathbf{-1}}\mathbf{C}_{\mathbf{uu}}^{(2)}))) \\ -\sigma_{e}^{2}\left(1-\mathbf{(x'x)}^{\mathbf{-}}\mathbf{x'C}_{\mathbf{uu}}^{(2)}\mathbf{x}\right)+(\sigma_{e}^{2}-\mathbf{\lambda}^{(2)}\sigma_{u}^{2})\mathbf{\lambda}^{(2)}\mathbf{(x'x)}^{\mathbf{-}}\mathbf{x'C}_{\mathbf{uu}}^{(2)}\mathbf{A}^{\mathbf{-1}}\mathbf{C}_{\mathbf{uu}}^{(2)}\mathbf{x} \end{matrix}$$

$\begin{matrix} =tr((\mathbf{I}-\mathbf{1}(\mathbf{1'1})^{-}\mathbf{1}')(\sigma_{e}^{2}(\mathbf{I}-\mathbf{C}_{\mathbf{uu}}^{(2)})-(\sigma_{e}^{2}-\mathbf{\lambda}^{(2)}\sigma_{u}^{2})(\mathbf{C}_{\mathbf{uu}}^{(2)}\mathbf{A}^{\mathbf{-1}}\mathbf{C}_{\mathbf{uu}}^{(2)}\mathbf{\lambda}^{(2)})) \\ -\sigma_{e}^{2}\left(1-\mathbf{(x'x)}^{\mathbf{-}}\mathbf{x'C}_{\mathbf{uu}}^{(2)}\mathbf{x}\right)+(\sigma_{e}^{2}-\mathbf{\lambda}^{(2)}\sigma_{u}^{2})\mathbf{\lambda}^{(2)}\mathbf{(x'x)}^{\mathbf{-}}\mathbf{x'C}_{\mathbf{uu}}^{(2)}\mathbf{A}^{\mathbf{-1}}\mathbf{C}_{\mathbf{uu}}^{(2)}\mathbf{x} \end{matrix}$.

Terms were then grouped depending on the variances:

$\begin{matrix} =\sigma_{e}^{2}(n-1-tr(\mathbf{C}_{\mathbf{uu}}^{(2)})+(\mathbf{1'1})^{-}\mathbf{1}'\mathbf{C}_{\mathbf{uu}}^{(2)}\mathbf{1})-(\sigma_{e}^{2}-\mathbf{\lambda}^{(2)}\sigma_{u}^{2})\left(\mathbf{\lambda}^{(2)}tr(\mathbf{C}_{\mathbf{uu}}^{(2)}\mathbf{A}^{\mathbf{-1}}\mathbf{C}_{\mathbf{uu}}^{(2)})-\mathbf{\lambda}^{(2)}(\mathbf{1'1})^{-}\mathbf{1}'\mathbf{C}_{\mathbf{uu}}^{(2)}\mathbf{A}^{\mathbf{-1}}\mathbf{C}_{\mathbf{uu}}^{(2)}\mathbf{1}\right) \\ -\sigma_{e}^{2}\left(1-\mathbf{(x'x)}^{\mathbf{-}}\mathbf{x'C}_{\mathbf{uu}}^{(2)}\mathbf{x}\right)+(\sigma_{e}^{2}-\mathbf{\lambda}^{(2)}\sigma_{u}^{2})\mathbf{\lambda}^{(2)}\mathbf{(x'x)}^{\mathbf{-}}\mathbf{x'C}_{\mathbf{uu}}^{(2)}\mathbf{A}^{\mathbf{-1}}\mathbf{C}_{\mathbf{uu}}^{(2)}\mathbf{x} \end{matrix}$ $\begin{matrix} =\sigma_{e}^{2}(n-2-tr(\mathbf{C}_{\mathbf{uu}}^{(2)})+\mathbf{(x'x)}^{\mathbf{-}}\mathbf{x'C}_{\mathbf{uu}}^{(2)}\mathbf{x}+(\mathbf{1'1})^{-}\mathbf{1}'\mathbf{C}_{\mathbf{uu}}^{(2)}\mathbf{1}) \\ -(\sigma_{e}^{2}-\mathbf{\lambda}^{(2)}\sigma_{u}^{2})\mathbf{\lambda}^{(2)}\left(tr(\mathbf{C}_{\mathbf{uu}}^{(2)}\mathbf{A}^{\mathbf{-1}}\mathbf{C}_{\mathbf{uu}}^{(2)})-(\mathbf{1'1})^{-}\mathbf{1}'\mathbf{C}_{\mathbf{uu}}^{(2)}\mathbf{A}^{\mathbf{-1}}\mathbf{C}_{\mathbf{uu}}^{(2)}\mathbf{1}-\mathbf{(x'x)}^{\mathbf{-}}\mathbf{x'C}_{\mathbf{uu}}^{(2)}\mathbf{A}^{\mathbf{-1}}\mathbf{C}_{\mathbf{uu}}^{(2)}\mathbf{x}\right) \end{matrix}$.

## In the part with $E(\hat{\mathbf{e}}^{(2)})$

$$\begin{matrix} E(\hat{\mathbf{e}}^{(2a)})=(\mathbf{I}-\mathbf{1}\left[\begin{matrix} \mathbf{C}_{11}^{(2)} & \mathbf{C}_{\mathbf{1u}}^{(2)} \end{matrix}\right]\left[\begin{matrix} \mathbf{1'} \\ \mathbf{I} \end{matrix}\right]-\left[\begin{matrix} \mathbf{C}_{\mathbf{u1}}^{(2)} & \mathbf{C}_{\mathbf{uu}}^{(2)} \end{matrix}\right]\left[\begin{matrix} \mathbf{1'} \\ \mathbf{I} \end{matrix}\right])E(\mathbf{y}) \\ =(\mathbf{I}-\mathbf{1}\left[\begin{matrix} \mathbf{C}_{11}^{(2)} & \mathbf{C}_{\mathbf{1u}}^{(2)} \end{matrix}\right]\left[\begin{matrix} \mathbf{1'} \\ \mathbf{I} \end{matrix}\right]-\left[\begin{matrix} \mathbf{C}_{\mathbf{u1}}^{(2)} & \mathbf{C}_{\mathbf{uu}}^{(2)} \end{matrix}\right]\left[\begin{matrix} \mathbf{1'} \\ \mathbf{I} \end{matrix}\right])(\mathbf{1}\mu+\mathbf{x}\beta) \end{matrix}$$

$\begin{matrix} E(\hat{\mathbf{e}}^{(2)})==\mathbf{1}\mu+\mathbf{x}\beta-\mathbf{1}\left[\begin{matrix} \mathbf{C}_{11}^{(2)} & \mathbf{C}_{\mathbf{1u}}^{(2)} \end{matrix}\right]\left[\begin{matrix} \mathbf{1'} \\ \mathbf{I} \end{matrix}\right]\left[\begin{matrix} \mathbf{1} & \mathbf{x} \end{matrix}\right]\left[\begin{matrix} \mu\\ \beta\end{matrix}\right]-\left[\begin{matrix} \mathbf{C}_{\mathbf{u1}}^{(2)} & \mathbf{C}_{\mathbf{uu}}^{(2)} \end{matrix}\right]\left[\begin{matrix} \mathbf{1'} \\ \mathbf{I} \end{matrix}\right]\left[\begin{matrix} \mathbf{1} & \mathbf{x} \end{matrix}\right]\left[\begin{matrix} \mu\\ \beta\end{matrix}\right] \\ =\mathbf{1}\mu+\mathbf{x}\beta-\mathbf{1}\left[\begin{matrix} \mathbf{C}_{11}^{(2)} & \mathbf{C}_{\mathbf{1u}}^{(2)} \end{matrix}\right]\left[\begin{matrix} \mathbf{1'1} & 0 \\ \mathbf{1} & \mathbf{x} \end{matrix}\right]\left[\begin{matrix} \mu\\ \beta\end{matrix}\right]-\left[\begin{matrix} \mathbf{C}_{\mathbf{u1}}^{(2)} & \mathbf{C}_{\mathbf{uu}}^{(2)} \end{matrix}\right]\left[\begin{matrix} \mathbf{1'1} & 0 \\ \mathbf{1} & \mathbf{x} \end{matrix}\right]\left[\begin{matrix} \mu\\ \beta\end{matrix}\right] \end{matrix}$.

Using $\mathbf{x'1}=0$

$\begin{matrix} =\mathbf{1}\mu+\mathbf{x}\beta-\mathbf{1}\left[\begin{matrix} 1 & \mathbf{C}_{\mathbf{1u}}^{(2)}\mathbf{x} \end{matrix}\right]\left[\begin{matrix} \mu\\ \beta\end{matrix}\right]-\left[\begin{matrix} 0 & \mathbf{C}_{\mathbf{uu}}^{(2)}\mathbf{x} \end{matrix}\right]\left[\begin{matrix} \mu\\ \beta\end{matrix}\right] \\ =\mathbf{1}\mu+\mathbf{x}\beta-\mathbf{1}\mu-\mathbf{1C}_{\mathbf{1u}}^{(2)}\mathbf{x}\beta-\mathbf{C}_{\mathbf{uu}}^{(2)}\mathbf{x}\beta\\ =\left(\mathbf{I}-\mathbf{1C}_{\mathbf{1u}}^{(2)}-\mathbf{C}_{\mathbf{uu}}^{(2)}\right)\mathbf{x}\beta\end{matrix}$.

Thus:

$$E(\mathbf{e}^{(2)})=\left(\mathbf{I}-\mathbf{1C}_{\mathbf{1u}}^{(2)}-\mathbf{C}_{\mathbf{uu}}^{(2)}\right)\mathbf{x}\beta$$

and in the expression of , the term with $E(\mathbf{e}^{(2)})$in was:

$$\begin{matrix} E(\hat{\mathbf{e}}^{(2)})'(\mathbf{I}-\mathbf{x(x'x)}^{\mathbf{-}}\mathbf{x'}-\mathbf{1}(\mathbf{1'1})^{-}\mathbf{1}')E(\hat{\mathbf{e}}^{(2)}) \\ =\mathbf{x}'\left(\mathbf{I}-\mathbf{C}_{\mathbf{u1}}^{(2)}\mathbf{1}'-\mathbf{C}_{\mathbf{uu}}^{(2)}\right)(\mathbf{I}-\mathbf{x(x'x)}^{\mathbf{-}}\mathbf{x'}-\mathbf{1}(\mathbf{1'1})^{-}\mathbf{1}')\left(\mathbf{I}-\mathbf{1C}_{\mathbf{1u}}^{(2)}-\mathbf{C}_{\mathbf{uu}}^{(2)}\right)\mathbf{x}\beta^{2} \end{matrix}$$

$\begin{matrix} =\mathbf{x}'\left(\mathbf{I}-\mathbf{C}_{\mathbf{u1}}^{(2)}\mathbf{1}'-\mathbf{C}_{\mathbf{uu}}^{(2)}\right) \\ (\mathbf{I}-\mathbf{1C}_{\mathbf{1u}}^{(2)}-\mathbf{C}_{\mathbf{uu}}^{(2)}-\mathbf{x(x'x)}^{\mathbf{-}}\mathbf{x'}+\mathbf{x(x'x)}^{\mathbf{-}}\mathbf{x'1C}_{\mathbf{1u}}^{(2)}+\mathbf{x(x'x)}^{\mathbf{-}}\mathbf{x'C}_{\mathbf{uu}}^{(2)}-\mathbf{1}(\mathbf{1'1})^{-}\mathbf{1}'+\mathbf{1}(\mathbf{1'1})^{-}\mathbf{1}'\mathbf{1C}_{\mathbf{1u}}^{(2)}+\mathbf{1}(\mathbf{1'1})^{-}\mathbf{1}'\mathbf{C}_{\mathbf{uu}}^{(2)})\mathbf{x}\beta^{2} \end{matrix}$knowing that $\mathbf{1C}_{\mathbf{1u}}^{(2)}=\mathbf{1}(\mathbf{1'1})^{-}\mathbf{1}'\mathbf{1C}_{\mathbf{1u}}^{(2)}$and $\mathbf{x'1}=0$and $\mathbf{1}'\mathbf{x}=0$,

$$\begin{matrix} =\mathbf{x}'\left(\mathbf{I}-\mathbf{C}_{\mathbf{u1}}^{(2)}\mathbf{1}'-\mathbf{C}_{\mathbf{uu}}^{(2)}\right) \\ (\mathbf{I}-\mathbf{C}_{\mathbf{uu}}^{(2)}-\mathbf{(x'x)}^{\mathbf{-}}\mathbf{xx'}+\mathbf{x(x'x)}^{\mathbf{-}}\mathbf{x'C}_{\mathbf{uu}}^{(2)}+\mathbf{1}(\mathbf{1'1})^{-}\mathbf{1}'\mathbf{C}_{\mathbf{uu}}^{(2)})\mathbf{x}\beta^{2} \end{matrix}$$

$$\begin{matrix} =\mathbf{x}'(\mathbf{I}-\mathbf{C}_{\mathbf{uu}}^{(2)}-\mathbf{(x'x)}^{\mathbf{-}}\mathbf{xx'}+\mathbf{x(x'x)}^{\mathbf{-}}\mathbf{x'C}_{\mathbf{uu}}^{(2)} \\ +\mathbf{C}_{\mathbf{u1}}^{(2)}\mathbf{1}'\mathbf{C}_{\mathbf{uu}}^{(2)}-\mathbf{C}_{\mathbf{u1}}^{(2)}\mathbf{1}'\mathbf{1}(\mathbf{1'1})^{-}\mathbf{1}'\mathbf{C}_{\mathbf{uu}}^{(2)} \\ -\mathbf{C}_{\mathbf{uu}}^{(2)}+\mathbf{C}_{\mathbf{uu}}^{(2)}\mathbf{C}_{\mathbf{uu}}^{(2)}+\mathbf{C}_{\mathbf{uu}}^{(2)}\mathbf{(x'x)}^{\mathbf{-}}\mathbf{xx'}-\mathbf{C}_{\mathbf{uu}}^{(2)}\mathbf{x(x'x)}^{\mathbf{-}}\mathbf{x'C}_{\mathbf{uu}}^{(2)}-\mathbf{C}_{\mathbf{uu}}^{(2)}\mathbf{1}(\mathbf{1'1})^{-}\mathbf{1}'\mathbf{C}_{\mathbf{uu}}^{(2)})\mathbf{x}\beta^{2} \end{matrix}$$

$$\begin{matrix} =\mathbf{x}'(\mathbf{I}-2\mathbf{C}_{\mathbf{uu}}^{(2)}-\mathbf{(x'x)}^{\mathbf{-}}\mathbf{xx'}+2\mathbf{x(x'x)}^{\mathbf{-}}\mathbf{x'C}_{\mathbf{uu}}^{(2)} \\ +\mathbf{C}_{\mathbf{uu}}^{(2)}\mathbf{C}_{\mathbf{uu}}^{(2)}-\mathbf{C}_{\mathbf{uu}}^{(2)}\mathbf{x(x'x)}^{\mathbf{-}}\mathbf{x'C}_{\mathbf{uu}}^{(2)}-\mathbf{C}_{\mathbf{uu}}^{(2)}\mathbf{1}(\mathbf{1'1})^{-}\mathbf{1}'\mathbf{C}_{\mathbf{uu}}^{(2)})\mathbf{x}\beta^{2} \end{matrix}$$

$$\begin{matrix} =\mathbf{x}'\mathbf{x}-2\mathbf{x'C}_{\mathbf{uu}}^{(2)}\mathbf{x}-\mathbf{x'(x'x)}^{\mathbf{-}}\mathbf{xx'x}+2\mathbf{x'x(x'x)}^{\mathbf{-}}\mathbf{x'C}_{\mathbf{uu}}^{(2)}\mathbf{x} \\ +\mathbf{x}'\left(\mathbf{C}_{\mathbf{uu}}^{(2)}\mathbf{C}_{\mathbf{uu}}^{(2)}-\mathbf{C}_{\mathbf{uu}}^{(2)}\mathbf{x(x'x)}^{\mathbf{-}}\mathbf{x'C}_{\mathbf{uu}}^{(2)}-\mathbf{C}_{\mathbf{uu}}^{(2)}\mathbf{1}(\mathbf{1'1})^{-}\mathbf{1}'\mathbf{C}_{\mathbf{uu}}^{(2)}\right)\mathbf{x}\beta^{2} \\ =\mathbf{x}'\left(\mathbf{C}_{\mathbf{uu}}^{(2)}\mathbf{C}_{\mathbf{uu}}^{(2)}-\mathbf{C}_{\mathbf{uu}}^{(2)}\mathbf{x(x'x)}^{\mathbf{-}}\mathbf{x'C}_{\mathbf{uu}}^{(2)}-\mathbf{C}_{\mathbf{uu}}^{(2)}\mathbf{1}(\mathbf{1'1})^{-}\mathbf{1}'\mathbf{C}_{\mathbf{uu}}^{(2)}\right)\mathbf{x}\beta^{2} \end{matrix}$$

$\begin{matrix} =\mathbf{x}'\left(\mathbf{C}_{\mathbf{uu}}^{(2)}\mathbf{C}_{\mathbf{uu}}^{(2)}-\mathbf{C}_{\mathbf{uu}}^{(2)}\mathbf{x(x'x)}^{\mathbf{-}}\mathbf{x'C}_{\mathbf{uu}}^{(2)}-\mathbf{C}_{\mathbf{uu}}^{(2)}\mathbf{1}(\mathbf{1'1})^{-}\mathbf{1}'\mathbf{C}_{\mathbf{uu}}^{(2)}\right)\mathbf{x}\beta^{2} \\ =\beta^{2}\mathbf{x}'\mathbf{C}_{\mathbf{uu}}^{(2)}(\mathbf{I}-\mathbf{(x'x)}^{\mathbf{-}}\mathbf{xx'}-(\mathbf{1'1})^{-}\mathbf{11}')\mathbf{C}_{\mathbf{uu}}^{(2)}\mathbf{x} \end{matrix}$.

## Total of

.

## expression of terms with$\mathbf{A}^{-1}$:

To translate :

$\mathbf{\lambda}^{(2)}\left(tr(\mathbf{C}_{\mathbf{uu}}^{(2)}\mathbf{A}^{\mathbf{-1}}\mathbf{C}_{\mathbf{uu}}^{(2)})-(\mathbf{1'1})^{-}\mathbf{1}'\mathbf{C}_{\mathbf{uu}}^{(2)}\mathbf{A}^{\mathbf{-1}}\mathbf{C}_{\mathbf{uu}}^{(2)}\mathbf{1}-\mathbf{(x'x)}^{\mathbf{-}}\mathbf{x'C}_{\mathbf{uu}}^{(2)}\mathbf{A}^{\mathbf{-1}}\mathbf{C}_{\mathbf{uu}}^{(2)}\mathbf{x}\right)$,

we know that

$\begin{matrix} \mathbf{C}_{\mathbf{u1}}^{(2)}\mathbf{1'}+\mathbf{C}_{\mathbf{uu}}^{(2)}=\mathbf{I}-\mathbf{C}_{\mathbf{uu}}^{(2)}\mathbf{\lambda}^{(2)}\mathbf{A}^{\mathbf{-1}} \\ \mathbf{C}_{\mathbf{uu}}^{(2)}\mathbf{\lambda}^{(2)}\mathbf{A}^{\mathbf{-1}}=\mathbf{I}-\mathbf{C}_{\mathbf{u1}}^{(2)}\mathbf{1'}-\mathbf{C}_{\mathbf{uu}}^{(2)} \\ \mathbf{C}_{\mathbf{uu}}^{(2)}\mathbf{\lambda}^{(2)}\mathbf{A}^{\mathbf{-1}}=\mathbf{I}+\frac{1}{n}\mathbf{C}_{\mathbf{uu}}^{(2)}\mathbf{11'}-\mathbf{C}_{\mathbf{uu}}^{(2)} \\ \mathbf{C}_{\mathbf{uu}}^{(2)}\mathbf{\lambda}^{(2)}\mathbf{A}^{\mathbf{-1}}=\mathbf{I}-\mathbf{C}_{\mathbf{uu}}^{(2)}\left(\mathbf{I}-\frac{1}{n}\mathbf{11'}\right) \end{matrix}$.

Thus:

$\begin{matrix} \mathbf{\lambda}^{(2)}\left(tr(\mathbf{C}_{\mathbf{uu}}^{(2)}\mathbf{A}^{\mathbf{-1}}\mathbf{C}_{\mathbf{uu}}^{(2)})-(\mathbf{1'1})^{-}\mathbf{1}'\mathbf{C}_{\mathbf{uu}}^{(2)}\mathbf{A}^{\mathbf{-1}}\mathbf{C}_{\mathbf{uu}}^{(2)}\mathbf{1}-\mathbf{(x'x)}^{\mathbf{-}}\mathbf{x'C}_{\mathbf{uu}}^{(2)}\mathbf{A}^{\mathbf{-1}}\mathbf{C}_{\mathbf{uu}}^{(2)}\mathbf{x}\right) \\ =tr(\left(\mathbf{I}-\mathbf{C}_{\mathbf{uu}}^{(2)}\left(\mathbf{I}-\frac{1}{n}\mathbf{11'}\right)\right)\mathbf{C}_{\mathbf{uu}}^{(2)})-(\mathbf{1'1})^{-}\mathbf{1}'\left(\mathbf{I}-\mathbf{C}_{\mathbf{uu}}^{(2)}\left(\mathbf{I}-\frac{1}{n}\mathbf{11'}\right)\right)\mathbf{C}_{\mathbf{uu}}^{(2)}\mathbf{1}-\mathbf{(x'x)}^{\mathbf{-}}\mathbf{x'}\left(\mathbf{I}-\mathbf{C}_{\mathbf{uu}}^{(2)}\left(\mathbf{I}-\frac{1}{n}\mathbf{11'}\right)\right)\mathbf{C}_{\mathbf{uu}}^{(2)}\mathbf{x} \\ =tr(\mathbf{C}_{\mathbf{uu}}^{(2)})-tr(\mathbf{C}_{\mathbf{uu}}^{(2)}\mathbf{C}_{\mathbf{uu}}^{(2)})+\frac{1}{n}\mathbf{1'C}_{\mathbf{uu}}^{(2)}\mathbf{C}_{\mathbf{uu}}^{(2)}\mathbf{1}-\frac{1}{n}\mathbf{1}'\mathbf{C}_{\mathbf{uu}}^{(2)}\mathbf{1}+\frac{1}{n}\mathbf{1}'\mathbf{C}_{\mathbf{uu}}^{(2)}\mathbf{C}_{\mathbf{uu}}^{(2)}\mathbf{1}-\frac{1}{n^{2}}\mathbf{1}'\mathbf{C}_{\mathbf{uu}}^{(2)}\mathbf{11'C}_{\mathbf{uu}}^{(2)}\mathbf{1}-\mathbf{(x'x)}^{\mathbf{-}}\mathbf{x'C}_{\mathbf{uu}}^{(2)}\mathbf{x}+\mathbf{(x'x)}^{\mathbf{-}}\mathbf{x'C}_{\mathbf{uu}}^{(2)}\mathbf{C}_{\mathbf{uu}}^{(2)}\mathbf{x}-\frac{1}{n}\mathbf{(x'x)}^{\mathbf{-}}\mathbf{x'C}_{\mathbf{uu}}^{(2)}\mathbf{11}'\mathbf{C}_{\mathbf{uu}}^{(2)}\mathbf{x} \end{matrix}$ $\begin{matrix} =tr(\mathbf{C}_{\mathbf{uu}}^{(2)})-\frac{1}{n}\mathbf{1}'\mathbf{C}_{\mathbf{uu}}^{(2)}\mathbf{1}+\frac{1}{n}\mathbf{1'C}_{\mathbf{uu}}^{(2)}\mathbf{C}_{\mathbf{uu}}^{(2)}\mathbf{1}-tr(\mathbf{C}_{\mathbf{uu}}^{(2)}\mathbf{C}_{\mathbf{uu}}^{(2)})+\frac{1}{n}\mathbf{1}'\mathbf{C}_{\mathbf{uu}}^{(2)}\mathbf{C}_{\mathbf{uu}}^{(2)}\mathbf{1}-\frac{1}{n^{2}}\mathbf{1}'\mathbf{C}_{\mathbf{uu}}^{(2)}\mathbf{11'C}_{\mathbf{uu}}^{(2)}\mathbf{1} \\ -\mathbf{(x'x)}^{\mathbf{-}}\mathbf{x'C}_{\mathbf{uu}}^{(2)}\mathbf{x}+\mathbf{(x'x)}^{\mathbf{-}}\mathbf{x'C}_{\mathbf{uu}}^{(2)}\mathbf{C}_{\mathbf{uu}}^{(2)}\mathbf{x}-\frac{1}{n}\mathbf{(x'x)}^{\mathbf{-}}\mathbf{x'C}_{\mathbf{uu}}^{(2)}\mathbf{11}'\mathbf{C}_{\mathbf{uu}}^{(2)}\mathbf{x} \\ =tr(\mathbf{C}_{\mathbf{uu}}^{(2)})-\frac{1}{n}\mathbf{1}'\mathbf{C}_{\mathbf{uu}}^{(2)}\mathbf{1}+2\left(\frac{1}{n}\mathbf{1}'\mathbf{C}_{\mathbf{uu}}^{(2)}\mathbf{C}_{\mathbf{uu}}^{(2)}\mathbf{1}-\frac{1}{n^{2}}\mathbf{1}'\mathbf{C}_{\mathbf{uu}}^{(2)}\mathbf{11'C}_{\mathbf{uu}}^{(2)}\mathbf{1}\right)-\left(tr(\mathbf{C}_{\mathbf{uu}}^{(2)}\mathbf{C}_{\mathbf{uu}}^{(2)})-\frac{1}{n^{2}}\mathbf{1}'\mathbf{C}_{\mathbf{uu}}^{(2)}\mathbf{11'C}_{\mathbf{uu}}^{(2)}\mathbf{1}\right) \\ -\mathbf{(x'x)}^{\mathbf{-}}\mathbf{x'C}_{\mathbf{uu}}^{(2)}\mathbf{x}+\mathbf{(x'x)}^{\mathbf{-}}\mathbf{x'C}_{\mathbf{uu}}^{(2)}\mathbf{C}_{\mathbf{uu}}^{(2)}\mathbf{x}-\frac{1}{n}\mathbf{(x'x)}^{\mathbf{-}}\mathbf{x'C}_{\mathbf{uu}}^{(2)}\mathbf{11}'\mathbf{C}_{\mathbf{uu}}^{(2)}\mathbf{x} \end{matrix}$.

# model3: FASTA model

$$E(\hat{\beta}^{(3)})\mathbf{=}E(\mathbf{C}_{\beta1}^{(3)}\mathbf{1'y}+\mathbf{C}_{\beta\beta}^{(3)}\mathbf{x'y}+\mathbf{C}_{\beta u}^{(3)}\mathbf{y})=\beta$$

We used:

$$\left[\begin{matrix} \mathbf{C}_{11}^{(3)} & \mathbf{C}_{1\beta}^{(3)} & \mathbf{C}_{1u}^{(3)} \\ \mathbf{C}_{\beta1}^{(3)} & \mathbf{C}_{\beta\beta}^{(3)} & \mathbf{C}_{\beta u}^{(3)} \\ \mathbf{C}_{u1}^{(3)} & \mathbf{C}_{u\beta}^{(3)} & \mathbf{C}_{uu}^{(3)} \end{matrix}\right]\left[\begin{matrix} \mathbf{1'1} & \mathbf{1'x} & \mathbf{1'} \\ \mathbf{x}'\mathbf{1} & \mathbf{x}'\mathbf{x} & \mathbf{x}' \\ \mathbf{1} & \mathbf{x} & \mathbf{I+\lambda}^{(2a)}\mathbf{A}^{\mathbf{-1}} \end{matrix}\right]=\left[\begin{matrix} 1 & 0 & \mathbf{0} \\ 0 & 1 & \mathbf{0} \\ \mathbf{0} & \mathbf{0} & \mathbf{I} \end{matrix}\right]$$

$$\left[\begin{matrix} \mathbf{1'1} & \mathbf{1'x} & \mathbf{1'} \\ \mathbf{x}'\mathbf{1} & \mathbf{x}'\mathbf{x} & \mathbf{x}' \\ \mathbf{1} & \mathbf{x} & \mathbf{I+\lambda}^{(2a)}\mathbf{A}^{\mathbf{-1}} \end{matrix}\right]\left[\begin{matrix} \mathbf{C}_{11}^{(3)} & \mathbf{C}_{1\beta}^{(3)} & \mathbf{C}_{1u}^{(3)} \\ \mathbf{C}_{\beta1}^{(3)} & \mathbf{C}_{\beta\beta}^{(3)} & \mathbf{C}_{\beta u}^{(3)} \\ \mathbf{C}_{u1}^{(3)} & \mathbf{C}_{u\beta}^{(3)} & \mathbf{C}_{uu}^{(3)} \end{matrix}\right]=\left[\begin{matrix} 1 & 0 & \mathbf{0} \\ 0 & 1 & \mathbf{0} \\ \mathbf{0} & \mathbf{0} & \mathbf{I} \end{matrix}\right]$$

$$\left[\begin{matrix} \mathbf{1'1} & \mathbf{1'x} & \mathbf{1'} \\ \mathbf{x}'\mathbf{1} & \mathbf{x}'\mathbf{x} & \mathbf{x}' \\ \mathbf{1} & \mathbf{x} & \mathbf{I} \end{matrix}\right]\left[\begin{matrix} \mathbf{C}_{11}^{(3)} & \mathbf{C}_{1\beta}^{(3)} & \mathbf{C}_{1u}^{(3)} \\ \mathbf{C}_{\beta1}^{(3)} & \mathbf{C}_{\beta\beta}^{(3)} & \mathbf{C}_{\beta u}^{(3)} \\ \mathbf{C}_{u1}^{(3)} & \mathbf{C}_{u\beta}^{(3)} & \mathbf{C}_{uu}^{(3)} \end{matrix}\right]=\left[\begin{matrix} 1 & 0 & \mathbf{0} \\ 0 & 1 & \mathbf{0} \\ \mathbf{0} & \mathbf{0} & \mathbf{I} \end{matrix}\right]-\left[\begin{matrix} 0 & 0 & \mathbf{0} \\ 0 & 0 & \mathbf{0} \\ \mathbf{0} & \mathbf{0} & \mathbf{\lambda}^{(2a)}\mathbf{A}^{\mathbf{-1}} \end{matrix}\right]\left[\begin{matrix} \mathbf{C}_{11}^{(3)} & \mathbf{C}_{1\beta}^{(3)} & \mathbf{C}_{1u}^{(3)} \\ \mathbf{C}_{\beta1}^{(3)} & \mathbf{C}_{\beta\beta}^{(3)} & \mathbf{C}_{\beta u}^{(3)} \\ \mathbf{C}_{u1}^{(3)} & \mathbf{C}_{u\beta}^{(3)} & \mathbf{C}_{uu}^{(3)} \end{matrix}\right]$$

$\left[\begin{matrix} \mathbf{1'1} & \mathbf{1'x} & \mathbf{1'} \\ \mathbf{x}'\mathbf{1} & \mathbf{x}'\mathbf{x} & \mathbf{x}' \\ \mathbf{1} & \mathbf{x} & \mathbf{I} \end{matrix}\right]\left[\begin{matrix} \mathbf{C}_{11}^{(3)} & \mathbf{C}_{1\beta}^{(3)} & \mathbf{C}_{1u}^{(3)} \\ \mathbf{C}_{\beta1}^{(3)} & \mathbf{C}_{\beta\beta}^{(3)} & \mathbf{C}_{\beta u}^{(3)} \\ \mathbf{C}_{u1}^{(3)} & \mathbf{C}_{u\beta}^{(3)} & \mathbf{C}_{uu}^{(3)} \end{matrix}\right]=\left[\begin{matrix} 1 & 0 & \mathbf{0} \\ 0 & 1 & \mathbf{0} \\ \mathbf{0} & \mathbf{0} & \mathbf{I} \end{matrix}\right]-\left[\begin{matrix} 0 & 0 & \mathbf{0} \\ 0 & 0 & \mathbf{0} \\ \mathbf{\lambda}^{(2a)}\mathbf{A}^{\mathbf{-1}}\mathbf{C}_{u1}^{(3)} & \mathbf{\lambda}^{(2a)}\mathbf{A}^{\mathbf{-1}}\mathbf{C}_{u\beta}^{(3)} & \mathbf{\lambda}^{(2a)}\mathbf{A}^{\mathbf{-1}}\mathbf{C}_{uu}^{(3)} \end{matrix}\right]$.

## Calculation of $V(\hat{\beta}^{(3)})$

$$\begin{matrix} V(\hat{\beta})\mathbf{=}V(\mathbf{C}_{\beta1}\mathbf{1'y}+\mathbf{C}_{\beta\beta}\mathbf{x'y}+\mathbf{C}_{\beta u}\mathbf{y}) \\ =(\mathbf{C}_{\beta1}\mathbf{1'}+\mathbf{C}_{\beta\beta}\mathbf{x'}+\mathbf{C}_{\beta u})(\mathbf{A}\sigma_{u}^{2}+\mathbf{I}\sigma_{e}^{2})(\mathbf{1C}_{1\beta}+\mathbf{xC}_{\beta\beta}+\mathbf{C}_{u\beta}) \\ =\sigma_{u}^{2}\left(\left[\begin{matrix} \mathbf{C}_{\beta1} & \mathbf{C}_{\beta\beta} & \mathbf{C}_{\beta u} \end{matrix}\right]\left[\begin{matrix} \mathbf{1'} \\ \mathbf{x'} \\ \mathbf{I} \end{matrix}\right]\mathbf{A}\left[\begin{matrix} \mathbf{1} & \mathbf{x} & \mathbf{I} \end{matrix}\right]\left[\begin{matrix} \mathbf{C}_{1\beta} \\ \mathbf{C}_{\beta\beta} \\ \mathbf{C}_{u\beta} \end{matrix}\right]+\frac{\sigma_{e}^{2}}{\sigma_{u}^{2}}\left[\begin{matrix} \mathbf{C}_{\beta1} & \mathbf{C}_{\beta\beta} & \mathbf{C}_{\beta u} \end{matrix}\right]\left[\begin{matrix} \mathbf{1'} \\ \mathbf{x'} \\ \mathbf{I} \end{matrix}\right]\left[\begin{matrix} \mathbf{1} & \mathbf{x} & \mathbf{I} \end{matrix}\right]\left[\begin{matrix} \mathbf{C}_{1\beta} \\ \mathbf{C}_{\beta\beta} \\ \mathbf{C}_{u\beta} \end{matrix}\right]\right) \\ =\sigma_{u}^{2}\left(\left[-\lambda^{(2)}\mathbf{C}_{\beta u}\mathbf{A}^{-1}\right]\mathbf{A}\left[-\lambda^{(2)}\mathbf{A}^{-1}\mathbf{C}_{u\beta}\right]+\frac{\sigma_{e}^{2}}{\sigma_{u}^{2}}\left[\begin{matrix} \mathbf{C}_{\beta1} & \mathbf{C}_{\beta\beta} & \mathbf{C}_{\beta u} \end{matrix}\right]\left[\begin{matrix} \mathbf{1'1} & \mathbf{1'1} & \mathbf{1'} \\ \mathbf{x'1} & \mathbf{x'x} & \mathbf{x'} \\ \mathbf{1} & \mathbf{x} & \mathbf{I} \end{matrix}\right]\left[\begin{matrix} \mathbf{C}_{1\beta} \\ \mathbf{C}_{\beta\beta} \\ \mathbf{C}_{u\beta} \end{matrix}\right]\right) \end{matrix}$$

$\begin{matrix} V(\hat{\beta})=\sigma_{u}^{2}\left(\lambda^{(2a)}\lambda^{(2a)}\mathbf{C}_{\beta u}\mathbf{A}^{-1}\mathbf{C}_{u\beta}+\frac{\sigma_{e}^{2}}{\sigma_{u}^{2}}(\mathbf{C}_{\beta\beta}-\lambda^{(2a)}\mathbf{C}_{\beta u}\mathbf{A}^{-1}\mathbf{C}_{u\beta})\right) \\ V(\hat{\beta}^{(3)})=\sigma_{e}^{2}\mathbf{C}_{\beta\beta}^{(3)}-(\sigma_{e}^{2}-\lambda^{(2a)}\sigma_{u}^{2})\lambda^{(2a)}\mathbf{C}_{\beta u}^{(3)}\mathbf{A}^{-1}\mathbf{C}_{u\beta}^{(3)} \end{matrix}$.

And with : $\mathbf{C}_{\beta u}^{(3)}=-\mathbf{C}_{\beta\beta}^{(3)}\mathbf{x}'\mathbf{C}_{uu}^{(2a)}$, $\mathbf{C}_{\beta\beta}^{(3)}=\left[\mathbf{x}'\mathbf{x}-\mathbf{x}'\mathbf{C}_{uu}^{(2a)}\mathbf{x}\right]^{-1}$and $\lambda^{(2a)}\mathbf{C}_{uu}^{(2a)}\mathbf{A}^{-1}=\mathbf{I}-\mathbf{C}_{u1}^{(2a)}\mathbf{1}'-\mathbf{C}_{uu}^{(2a)}$

$$V(\hat{\beta}^{(3)})=\sigma_{e}^{2}\mathbf{C}_{\beta\beta}^{(3)}-(\sigma_{e}^{2}-\lambda^{(2a)}\sigma_{u}^{2})\lambda^{(2a)}\mathbf{C}_{\beta\beta}^{(3)}\mathbf{x}'\mathbf{C}_{uu}^{(2a)}\mathbf{A}^{-1}\mathbf{C}_{uu}^{(2a)}\mathbf{xC}_{\beta\beta}^{(3)}$$

$$V(\hat{\beta}^{(3)})=\sigma_{e}^{2}\mathbf{C}_{\beta\beta}^{(3)}-(\sigma_{e}^{2}-\lambda^{(2a)}\sigma_{u}^{2})\lambda^{(2a)}\left[\mathbf{x}'\mathbf{x}-\mathbf{x}'\mathbf{C}_{uu}^{(2a)}\mathbf{x}\right]^{-1}\mathbf{x}'\mathbf{C}_{uu}^{(2a)}\mathbf{A}^{-1}\mathbf{C}_{uu}^{(2a)}\mathbf{x}\left[\mathbf{x}'\mathbf{x}-\mathbf{x}'\mathbf{C}_{uu}^{(2a)}\mathbf{x}\right]^{-1}$$

$$V(\hat{\beta}^{(3)})=\sigma_{e}^{2}\mathbf{C}_{\beta\beta}^{(3)}-(\sigma_{e}^{2}-\lambda^{(2a)}\sigma_{u}^{2})\left[\mathbf{x}'\mathbf{x}-\mathbf{x}'\mathbf{C}_{uu}^{(2a)}\mathbf{x}\right]^{-2}\mathbf{x}'\left(\mathbf{I}-\mathbf{C}_{u1}^{(2a)}\mathbf{1}'-\mathbf{C}_{uu}^{(2a)}\right)\mathbf{C}_{uu}^{(2a)}\mathbf{x}$$

$V(\hat{\beta}^{(3)})=\sigma_{e}^{2}\left[\mathbf{x}'\mathbf{x}-\mathbf{x}'\mathbf{C}_{uu}^{(2a)}\mathbf{x}\right]^{-1}-(\sigma_{e}^{2}-\lambda^{(2a)}\sigma_{u}^{2})\left[\mathbf{x}'\mathbf{x}-\mathbf{x}'\mathbf{C}_{uu}^{(2a)}\mathbf{x}\right]^{-2}\left(\mathbf{x}'\mathbf{C}_{uu}^{(2a)}\mathbf{x}-\mathbf{x}'\mathbf{C}_{uu}^{(2a)}\mathbf{C}_{uu}^{(2a)}\mathbf{x}\right)$.

## Calculation of

$$\hat{\mathbf{e}}^{(3)}=\left(\mathbf{I}-\left[\begin{matrix} \mathbf{1} & \mathbf{x} & \mathbf{I} \end{matrix}\right]\left[\begin{matrix} \mathbf{1'1} & \mathbf{1'x} & \mathbf{1'} \\ \mathbf{x}'\mathbf{1} & \mathbf{x}'\mathbf{x} & \mathbf{x}' \\ \mathbf{1} & \mathbf{x} & \mathbf{I+\lambda}^{(2a)}\mathbf{A}^{\mathbf{-1}} \end{matrix}\right]^{-}\left[\begin{matrix} \mathbf{1'} \\ \mathbf{x'} \\ \mathbf{I} \end{matrix}\right]\right)\mathbf{y}$$

.

## In the part with the trace:

$$\begin{matrix} =\sigma_{u}^{2}tr(\left(\mathbf{I}-\left[\begin{matrix} \mathbf{1} & \mathbf{x} & \mathbf{I} \end{matrix}\right]\left[\begin{matrix} \mathbf{1'1} & \mathbf{1'x} & \mathbf{1'} \\ \mathbf{x}'\mathbf{1} & \mathbf{x}'\mathbf{x} & \mathbf{x}' \\ \mathbf{1} & \mathbf{x} & \mathbf{I+\lambda}^{(2a)}\mathbf{A}^{\mathbf{-1}} \end{matrix}\right]^{-}\left[\begin{matrix} \mathbf{1'} \\ \mathbf{x'} \\ \mathbf{I} \end{matrix}\right]\right)\mathbf{A}) \\ +\sigma_{e}^{2}tr\left(\mathbf{I}-\left[\begin{matrix} \mathbf{1} & \mathbf{x} & \mathbf{I} \end{matrix}\right]\left[\begin{matrix} \mathbf{1'1} & \mathbf{1'x} & \mathbf{1'} \\ \mathbf{x}'\mathbf{1} & \mathbf{x}'\mathbf{x} & \mathbf{x}' \\ \mathbf{1} & \mathbf{x} & \mathbf{I+\lambda}^{(2a)}\mathbf{A}^{\mathbf{-1}} \end{matrix}\right]^{-}\left[\begin{matrix} \mathbf{1'} \\ \mathbf{x'} \\ \mathbf{I} \end{matrix}\right]\right) \end{matrix}$$

$$\begin{matrix} =\sigma_{u}^{2}tr(\left(\mathbf{I}-\left[\begin{matrix} -\lambda^{(2a)}\mathbf{A}^{-1}\mathbf{C}_{u1}^{(3)} & -\lambda^{(2a)}\mathbf{A}^{-1}\mathbf{C}_{u\beta}^{(3)} & \mathbf{I}-\lambda^{(2a)}\mathbf{A}^{-1}\mathbf{C}_{uu}^{(3)} \end{matrix}\right]\left[\begin{matrix} \mathbf{1'} \\ \mathbf{x'} \\ \mathbf{I} \end{matrix}\right]\right)\mathbf{A}) \\ +\sigma_{e}^{2}\left(n-tr\left(\left[\begin{matrix} 1 & 0 & \mathbf{0} \\ 0 & 1 & \mathbf{0} \\ \mathbf{0} & \mathbf{0} & \mathbf{I}-\lambda^{(2a)}\mathbf{C}_{uu}^{(3)}\mathbf{A}^{-1} \end{matrix}\right]\right)\right) \end{matrix}$$

$$=\sigma_{u}^{2}tr\left(\mathbf{A}+\lambda^{(2a)}\mathbf{C}_{u1}^{(3)}\mathbf{1}'+\lambda^{(2a)}\mathbf{C}_{u\beta}^{(3)}\mathbf{x}'-\mathbf{A}+\lambda^{(2a)}\mathbf{C}_{uu}^{(3)}\right)+\sigma_{e}^{2}\left(tr(\lambda^{(2a)}\mathbf{A}^{-1}\mathbf{C}_{uu}^{(3)})-2\right)$$

$$=\sigma_{u}^{2}tr\left(\lambda^{(2a)}(\mathbf{I}-\lambda^{(2a)}\mathbf{A}^{-1}\mathbf{C}_{uu}^{(3)})\right)+\sigma_{e}^{2}\left(tr(\lambda^{(2a)}\mathbf{A}^{-1}\mathbf{C}_{uu}^{(3)})-2\right)$$

$=n\lambda^{(2a)}\sigma_{u}^{2}-2\sigma_{e}^{2}+(\sigma_{e}^{2}-\lambda^{(2a)}\sigma_{u}^{2})\lambda^{(2a)}tr(\mathbf{A}^{-1}\mathbf{C}_{uu}^{(3)})$.

## In the Part with the expectation:

$\begin{matrix} E(\mathbf{y})'\left(\mathbf{I}-\left[\begin{matrix} \mathbf{1} & \mathbf{x} & \mathbf{I} \end{matrix}\right]\left[\begin{matrix} \mathbf{1'1} & \mathbf{1'x} & \mathbf{1'} \\ \mathbf{x}'\mathbf{1} & \mathbf{x}'\mathbf{x} & \mathbf{x}' \\ \mathbf{1} & \mathbf{x} & \mathbf{I+\lambda}^{(2a)}\mathbf{A}^{\mathbf{-1}} \end{matrix}\right]^{-}\left[\begin{matrix} \mathbf{1'} \\ \mathbf{x'} \\ \mathbf{I} \end{matrix}\right]\right)E(\mathbf{y}) \\ =\left[\begin{matrix} \mu& \beta\end{matrix}\right]\left[\begin{matrix} \mathbf{1'} \\ \mathbf{x}' \end{matrix}\right]\left(\mathbf{I}-\left[\begin{matrix} \mathbf{1} & \mathbf{x} & \mathbf{I} \end{matrix}\right]\left[\begin{matrix} \mathbf{1'1} & \mathbf{1'x} & \mathbf{1'} \\ \mathbf{x}'\mathbf{1} & \mathbf{x}'\mathbf{x} & \mathbf{x}' \\ \mathbf{1} & \mathbf{x} & \mathbf{I+\lambda}^{(2a)}\mathbf{A}^{\mathbf{-1}} \end{matrix}\right]^{-}\left[\begin{matrix} \mathbf{1'} \\ \mathbf{x'} \\ \mathbf{I} \end{matrix}\right]\right)\left[\begin{matrix} \mathbf{1} & \mathbf{x} \end{matrix}\right]\left[\begin{matrix} \mu\\ \beta\end{matrix}\right] \\ =\left[\begin{matrix} \mu& \beta\end{matrix}\right]\left[\begin{matrix} \mathbf{1'1} & \mathbf{1'x} \\ \mathbf{x}'\mathbf{1} & \mathbf{x}'\mathbf{x} \end{matrix}\right]\left[\begin{matrix} \mu\\ \beta\end{matrix}\right]-\left[\begin{matrix} \mu& \beta\end{matrix}\right]\left[\begin{matrix} \mathbf{1'1} & \mathbf{1'x} & \mathbf{1'} \\ \mathbf{x}'\mathbf{1} & \mathbf{x}'\mathbf{x} & \mathbf{x}' \end{matrix}\right]\left[\begin{matrix} \mathbf{1'1} & \mathbf{1'x} & \mathbf{1'} \\ \mathbf{x}'\mathbf{1} & \mathbf{x}'\mathbf{x} & \mathbf{x}' \\ \mathbf{1} & \mathbf{x} & \mathbf{I+\lambda}^{(2a)}\mathbf{A}^{\mathbf{-1}} \end{matrix}\right]^{-}\left[\begin{matrix} \mathbf{1'1} & \mathbf{1'x} \\ \mathbf{x}'\mathbf{1} & \mathbf{x}'\mathbf{x} \\ \mathbf{1} & \mathbf{x} \end{matrix}\right]\left[\begin{matrix} \mu\\ \beta\end{matrix}\right] \\ =\left[\begin{matrix} \mu& \beta\end{matrix}\right]\left[\begin{matrix} \mathbf{1'1} & \mathbf{1'x} \\ \mathbf{x}'\mathbf{1} & \mathbf{x}'\mathbf{x} \end{matrix}\right]\left[\begin{matrix} \mu\\ \beta\end{matrix}\right]-\left[\begin{matrix} \mu& \beta\end{matrix}\right]\left[\begin{matrix} 1 & 0 & \mathbf{0} \\ 0 & 1 & \mathbf{0} \end{matrix}\right]\left[\begin{matrix} \mathbf{1'1} & \mathbf{1'x} \\ \mathbf{x}'\mathbf{1} & \mathbf{x}'\mathbf{x} \\ \mathbf{1} & \mathbf{x} \end{matrix}\right]\left[\begin{matrix} \mu\\ \beta\end{matrix}\right] \\ =\left[\begin{matrix} \mu& \beta\end{matrix}\right]\left[\begin{matrix} \mathbf{1'1} & \mathbf{1'x} \\ \mathbf{x}'\mathbf{1} & \mathbf{x}'\mathbf{x} \end{matrix}\right]\left[\begin{matrix} \mu\\ \beta\end{matrix}\right]-\left[\begin{matrix} \mu& \beta\end{matrix}\right]\left[\begin{matrix} \mathbf{1'1} & \mathbf{1'x} \\ \mathbf{x}'\mathbf{1} & \mathbf{x}'\mathbf{x} \end{matrix}\right]\left[\begin{matrix} \mu\\ \beta\end{matrix}\right] \\ =0 \end{matrix}$.

## total of

If expression of $\lambda^{(2a)}tr(\mathbf{A}^{-1}\mathbf{C}_{uu}^{(3)})$uses terms with only $\mathbf{A}$and $\mathbf{C}_{\mathbf{uu}}^{(2)}$, we know that:

$\left[\begin{matrix} \mathbf{C}_{\beta\beta}^{(3)} & \mathbf{C}_{\beta1}^{(3)} & \mathbf{C}_{\beta u}^{(3)} \\ \mathbf{C}_{1\beta}^{(3)} & \mathbf{C}_{11}^{(3)} & \mathbf{C}_{1u}^{(3)} \\ \mathbf{C}_{u\beta}^{(3)} & \mathbf{C}_{u1}^{(3)} & \mathbf{C}_{uu}^{(3)} \end{matrix}\right]\left[\begin{matrix} \mathbf{x}'\mathbf{x} & \mathbf{x}'\mathbf{1} & \mathbf{x}' \\ \mathbf{1'x} & \mathbf{1'1} & \mathbf{1'} \\ \mathbf{x} & \mathbf{1} & \mathbf{I+\lambda}^{(2a)}\mathbf{A}^{\mathbf{-1}} \end{matrix}\right]=\left[\begin{matrix} 1 & 0 & \mathbf{0} \\ 0 & 1 & \mathbf{0} \\ \mathbf{0} & \mathbf{0} & \mathbf{I} \end{matrix}\right]$so :

$\left[\begin{matrix} \mathbf{C}_{11}^{(3)} & \mathbf{C}_{1u}^{(3)} \\ \mathbf{C}_{u1}^{(3)} & \mathbf{C}_{uu}^{(3)} \end{matrix}\right]=\left[\begin{matrix} \mathbf{1'1} & \mathbf{1'} \\ \mathbf{1} & \mathbf{I+\lambda}^{(2a)}\mathbf{A}^{\mathbf{-1}} \end{matrix}\right]^{-1}+\left[\begin{matrix} \mathbf{1'1} & \mathbf{1'} \\ \mathbf{1} & \mathbf{I+\lambda}^{(2a)}\mathbf{A}^{\mathbf{-1}} \end{matrix}\right]^{-1}\left[\begin{matrix} \mathbf{1'x} \\ \mathbf{x} \end{matrix}\right]\mathbf{C}_{\beta\beta}^{(3)}\left[\begin{matrix} \mathbf{x}'\mathbf{1} & \mathbf{x}' \end{matrix}\right]\left[\begin{matrix} \mathbf{1'1} & \mathbf{1'} \\ \mathbf{1} & \mathbf{I+\lambda}^{(2a)}\mathbf{A}^{\mathbf{-1}} \end{matrix}\right]^{-1}$,

and with the matrices used in GRAMMAR, the equalities were:

$$\left[\begin{matrix} \mathbf{C}_{11}^{(3)} & \mathbf{C}_{1u}^{(3)} \\ \mathbf{C}_{u1}^{(3)} & \mathbf{C}_{uu}^{(3)} \end{matrix}\right]=\left[\begin{matrix} \mathbf{C}_{11}^{(2a)} & \mathbf{C}_{1u}^{(2a)} \\ \mathbf{C}_{u1}^{(2a)} & \mathbf{C}_{uu}^{(2a)} \end{matrix}\right]+\left[\begin{matrix} \mathbf{C}_{11}^{(2a)} & \mathbf{C}_{1u}^{(2a)} \\ \mathbf{C}_{u1}^{(2a)} & \mathbf{C}_{uu}^{(2a)} \end{matrix}\right]\left[\begin{matrix} \mathbf{1'x} \\ \mathbf{x} \end{matrix}\right]\mathbf{C}_{\beta\beta}^{(3)}\left[\begin{matrix} \mathbf{x}'\mathbf{1} & \mathbf{x}' \end{matrix}\right]\left[\begin{matrix} \mathbf{C}_{11}^{(2a)} & \mathbf{C}_{1u}^{(2a)} \\ \mathbf{C}_{u1}^{(2a)} & \mathbf{C}_{uu}^{(2a)} \end{matrix}\right]$$

Thus:

$$\left[\begin{matrix} \mathbf{C}_{11}^{(3)} & \mathbf{C}_{1u}^{(3)} \\ \mathbf{C}_{u1}^{(3)} & \mathbf{C}_{uu}^{(3)} \end{matrix}\right]=\left[\begin{matrix} \mathbf{C}_{11}^{(2a)} & \mathbf{C}_{1u}^{(2a)} \\ \mathbf{C}_{u1}^{(2a)} & \mathbf{C}_{uu}^{(2a)} \end{matrix}\right]+\left[\begin{matrix} \mathbf{C}_{11}^{(2a)} & \mathbf{C}_{1u}^{(2a)} \\ \mathbf{C}_{u1}^{(2a)} & \mathbf{C}_{uu}^{(2a)} \end{matrix}\right]\left[\begin{matrix} \mathbf{1'xC}_{\beta\beta}^{(3)}\mathbf{x}'\mathbf{1} & \mathbf{1'xC}_{\beta\beta}^{(3)}\mathbf{x}' \\ \mathbf{xC}_{\beta\beta}^{(3)}\mathbf{x}'\mathbf{1} & \mathbf{xC}_{\beta\beta}^{(3)}\mathbf{x}' \end{matrix}\right]\left[\begin{matrix} \mathbf{C}_{11}^{(2a)} & \mathbf{C}_{1u}^{(2a)} \\ \mathbf{C}_{u1}^{(2a)} & \mathbf{C}_{uu}^{(2a)} \end{matrix}\right]$$

$$\left[\begin{matrix} \mathbf{C}_{11}^{(3)} & \mathbf{C}_{1u}^{(3)} \\ \mathbf{C}_{u1}^{(3)} & \mathbf{C}_{uu}^{(3)} \end{matrix}\right]=\left[\begin{matrix} \mathbf{C}_{11}^{(2a)} & \mathbf{C}_{1u}^{(2a)} \\ \mathbf{C}_{u1}^{(2a)} & \mathbf{C}_{uu}^{(2a)} \end{matrix}\right]+\left[\begin{matrix} \mathbf{C}_{11}^{(2a)} & \mathbf{C}_{1u}^{(2a)} \\ \mathbf{C}_{u1}^{(2a)} & \mathbf{C}_{uu}^{(2a)} \end{matrix}\right]\left[\begin{matrix} 0 & 0 \\ 0 & \mathbf{xC}_{\beta\beta}^{(3)}\mathbf{x}' \end{matrix}\right]\left[\begin{matrix} \mathbf{C}_{11}^{(2a)} & \mathbf{C}_{1u}^{(2a)} \\ \mathbf{C}_{u1}^{(2a)} & \mathbf{C}_{uu}^{(2a)} \end{matrix}\right]$$

$\begin{matrix} \left[\begin{matrix} \mathbf{C}_{11}^{(3)} & \mathbf{C}_{1u}^{(3)} \\ \mathbf{C}_{u1}^{(3)} & \mathbf{C}_{uu}^{(3)} \end{matrix}\right]=\left[\begin{matrix} \mathbf{C}_{11}^{(2a)} & \mathbf{C}_{1u}^{(2a)} \\ \mathbf{C}_{u1}^{(2a)} & \mathbf{C}_{uu}^{(2a)} \end{matrix}\right]+\left[\begin{matrix} 0 & \mathbf{C}_{1u}^{(2a)}\mathbf{xC}_{\beta\beta}^{(3)}\mathbf{x}' \\ 0 & \mathbf{C}_{uu}^{(2a)}\mathbf{xC}_{\beta\beta}^{(3)}\mathbf{x}' \end{matrix}\right]\left[\begin{matrix} \mathbf{C}_{11}^{(2a)} & \mathbf{C}_{1u}^{(2a)} \\ \mathbf{C}_{u1}^{(2a)} & \mathbf{C}_{uu}^{(2a)} \end{matrix}\right] \\ \left[\begin{matrix} \mathbf{C}_{11}^{(3)} & \mathbf{C}_{1u}^{(3)} \\ \mathbf{C}_{u1}^{(3)} & \mathbf{C}_{uu}^{(3)} \end{matrix}\right]=\left[\begin{matrix} \mathbf{C}_{11}^{(2a)} & \mathbf{C}_{1u}^{(2a)} \\ \mathbf{C}_{u1}^{(2a)} & \mathbf{C}_{uu}^{(2a)} \end{matrix}\right]+\left[\begin{matrix} \mathbf{C}_{1u}^{(2a)}\mathbf{xC}_{\beta\beta}^{(3)}\mathbf{x}'\mathbf{C}_{u1}^{(2a)} & \mathbf{C}_{1u}^{(2a)}\mathbf{xC}_{\beta\beta}^{(3)}\mathbf{x}'\mathbf{C}_{uu}^{(2a)} \\ \mathbf{C}_{uu}^{(2a)}\mathbf{xC}_{\beta\beta}^{(3)}\mathbf{x}'\mathbf{C}_{u1}^{(2a)} & \mathbf{C}_{uu}^{(2a)}\mathbf{xC}_{\beta\beta}^{(3)}\mathbf{x}'\mathbf{C}_{uu}^{(2a)} \end{matrix}\right] \end{matrix}$.

Thus

$\mathbf{C}_{uu}^{(3)}=\mathbf{C}_{uu}^{(2)}+\mathbf{C}_{uu}^{(2a)}\mathbf{xC}_{\beta\beta}^{(3)}\mathbf{x}'\mathbf{C}_{uu}^{(2a)}$,

and since:

$\begin{matrix} \mathbf{C}_{\beta\beta}^{(3)}=\left(\mathbf{x}'\mathbf{x}-\left[\begin{matrix} \mathbf{x}'\mathbf{1} & \mathbf{x}' \end{matrix}\right]\left[\begin{matrix} \mathbf{C}_{11}^{(2a)} & \mathbf{C}_{1u}^{(2a)} \\ \mathbf{C}_{u1}^{(2a)} & \mathbf{C}_{uu}^{(2a)} \end{matrix}\right]\left[\begin{matrix} \mathbf{1'x} \\ \mathbf{x} \end{matrix}\right]\right)^{-1} \\ =\left(\mathbf{x}'\mathbf{x}-\left[\begin{matrix} 0 & \mathbf{x}' \end{matrix}\right]\left[\begin{matrix} \mathbf{C}_{11}^{(2a)} & \mathbf{C}_{1u}^{(2a)} \\ \mathbf{C}_{u1}^{(2a)} & \mathbf{C}_{uu}^{(2a)} \end{matrix}\right]\left[\begin{matrix} 0 \\ \mathbf{x} \end{matrix}\right]\right)^{-1} \\ =\left(\mathbf{x}'\mathbf{x}-\left[\begin{matrix} \mathbf{x}'\mathbf{C}_{u1}^{(2a)} & \mathbf{x}'\mathbf{C}_{uu}^{(2a)} \end{matrix}\right]\left[\begin{matrix} 0 \\ \mathbf{x} \end{matrix}\right]\right)^{-1} \\ =\left(\mathbf{x}'\mathbf{x}-\mathbf{x}'\mathbf{C}_{uu}^{(2a)}\mathbf{x}\right)^{-1} \end{matrix}$,

replacing $\mathbf{C}_{\beta\beta}^{(3)}$:

$$\begin{matrix} \mathbf{C}_{uu}^{(3)}=\mathbf{C}_{uu}^{(2a)}+\mathbf{C}_{uu}^{(2a)}\mathbf{xC}_{\beta\beta}^{(3)}\mathbf{x}'\mathbf{C}_{uu}^{(2a)} \\ \mathbf{C}_{uu}^{(3)}=\mathbf{C}_{uu}^{(2a)}+\mathbf{C}_{uu}^{(2a)}\mathbf{x}\left[\mathbf{x}'\mathbf{x}-\mathbf{x}'\mathbf{C}_{uu}^{(2a)}\mathbf{x}\right]^{-1}\mathbf{x}'\mathbf{C}_{uu}^{(2a)} \\ \mathbf{C}_{uu}^{(3)}=\mathbf{C}_{uu}^{(2a)}+\left[\mathbf{x}'\mathbf{x}-\mathbf{x}'\mathbf{C}_{uu}^{(2a)}\mathbf{x}\right]^{-1}\mathbf{C}_{uu}^{(2a)}\mathbf{xx}'\mathbf{C}_{uu}^{(2a)} \end{matrix}$$

the function of the trace becomes $\lambda^{(2a)}tr(\mathbf{A}^{-1}\mathbf{C}_{uu}^{(3)})$

$\begin{matrix} \lambda^{(2a)}tr(\mathbf{A}^{-1}\mathbf{C}_{uu}^{(3)})=\lambda^{(2a)}tr(\mathbf{A}^{-1}\left(\mathbf{C}_{uu}^{(2a)}+\left[\mathbf{x}'\mathbf{x}-\mathbf{x}'\mathbf{C}_{uu}^{(2)}\mathbf{x}\right]^{-1}\mathbf{C}_{uu}^{(2a)}\mathbf{xx}'\mathbf{C}_{uu}^{(2a)}\right)) \\ =\lambda^{(2a)}tr(\mathbf{A}^{-1}\mathbf{C}_{uu}^{(2a)})+\lambda^{(2a)}tr(\mathbf{A}^{-1}\mathbf{C}_{uu}^{(2a)}\mathbf{xx}'\mathbf{C}_{uu}^{(2a)})\left[\mathbf{x}'\mathbf{x}-\mathbf{x}'\mathbf{C}_{uu}^{(2a)}\mathbf{x}\right]^{-1} \\ =tr(\mathbf{I}-\mathbf{C}_{\mathbf{uu}}^{(2a)}\left(\mathbf{I}-\frac{1}{n}\mathbf{11'}\right))+\mathbf{x}'\left(\mathbf{I}-\mathbf{C}_{uu}^{(2a)}\left(\mathbf{I}-\frac{1}{n}\mathbf{11'}\right)\right)\mathbf{C}_{uu}^{(2a)}\mathbf{x}\left[\mathbf{x}'\mathbf{x}-\mathbf{x}'\mathbf{C}_{uu}^{(2a)}\mathbf{x}\right]^{-1} \\ =n-tr(\mathbf{C}_{\mathbf{uu}}^{(2a)})+\frac{1}{n}\mathbf{1'C}_{\mathbf{uu}}^{(2a)}\mathbf{1}+\frac{\mathbf{x}'\mathbf{C}_{uu}^{(2a)}\mathbf{x}-\mathbf{x}'\mathbf{C}_{uu}^{(2a)}\mathbf{C}_{uu}^{(2a)}\mathbf{x}+\mathbf{x}'\mathbf{C}_{uu}^{(2a)}\mathbf{11'C}_{uu}^{(2a)}\mathbf{x}/n}{\mathbf{x}'\mathbf{x}-\mathbf{x}'\mathbf{C}_{uu}^{(2a)}\mathbf{x}} \end{matrix}$,

and the expression of was

which was equal to

.

# Model 4: QTDT

For the QTDT method, many simplifications occurred when replacing $\mathbf{Q'Q}$by its expectation which was a diagonal matrix, easy to invert.

Thus:

$$E(\hat{\beta}_{w}^{(4)})=\frac{1}{tr(\mathbf{D})}(tr(\mathbf{D})\frac{n-1}{n})\beta=\frac{n-1}{n}\beta$$

$\begin{matrix} V(\hat{\beta}_{w}^{(4)})=\left[\mathbf{(Q'Q)}^{\mathbf{-}}\mathbf{Q'AQ(Q'Q)}^{\mathbf{-}}\right]_{3,3}\sigma_{u}^{2}+\left[\mathbf{(Q'Q)}^{\mathbf{-}}\right]_{¨3,3}\sigma_{e}^{2} \\ =\frac{1}{tr(\mathbf{D})}tr(\mathbf{AD})\frac{1}{tr(\mathbf{D})}\sigma_{u}^{2}+\frac{1}{tr(\mathbf{D})}\sigma_{e}^{2} \end{matrix}$.

And without inbreeding, $tr(\mathbf{AD})=tr(\mathbf{D})=\frac{n}{2}$thus:

$V(\hat{\beta}_{w}^{(4)})=\frac{2}{n}(\sigma_{u}^{2}+\sigma_{e}^{2})$.

When replacing $\mathbf{Q'Q}$by its expectation, the sum of the square of residuals was:

.

# Marginal expectation of quadratic forms according to distribution of genotypes

We will need $V(\mathbf{x})$.

The coefficients of the matrix $V(\mathbf{x})$were:

$$E((w_{i}-\bar{w})(w_{j}-\bar{w}))=E(w_{i}w_{j})-\frac{1}{n}\sum_{k=1}^{n} E(w_{i}w_{k})-\frac{1}{n}\sum_{k=1}^{n} E(w_{i}w_{k})+\frac{1}{n^{2}}\mathbf{1'A1}$$

$=a_{ij}-\frac{1}{n}\sum_{k=1}^{n} a_{ik}-\frac{1}{n}\sum_{k=1}^{n} a_{jk}+\frac{1}{n^{2}}\mathbf{1'A1}$.

Thus:

$V(\mathbf{x})=\mathbf{A}-\frac{1}{n}\mathbf{11}'\mathbf{A}-\frac{1}{n}\mathbf{A11}'+\frac{1}{n^{2}}\mathbf{11'A11}'$.

## Calculation of $E_{x}(\mathbf{x}'\mathbf{x})$

$\begin{matrix} E_{x}(\mathbf{x}'\mathbf{x})=tr(V(\mathbf{x}))+E(\mathbf{x}')E(\mathbf{x}') \\ =tr(\mathbf{A}-\frac{1}{n}\mathbf{11}'\mathbf{A}-\frac{1}{n}\mathbf{A11}'+\frac{1}{n^{2}}\mathbf{11'A11}') \\ =tr(\mathbf{A})-\frac{2}{n}\mathbf{1}'\mathbf{A1}+\frac{1}{n^{2}}\mathbf{1'A11'1} \\ =tr(\mathbf{A})-\frac{1}{n}\mathbf{1}'\mathbf{A1} \end{matrix}$.

## Calculation of $E_{x}(\mathbf{x}'\mathbf{Ax})$

$\begin{matrix} E_{x}(\mathbf{x}'\mathbf{Ax})=tr(\mathbf{A}V(\mathbf{x}))+E(\mathbf{x}')\mathbf{A}E(\mathbf{x}) \\ =tr(\mathbf{A}(\mathbf{A}-\frac{1}{n}\mathbf{11}'\mathbf{A}-\frac{1}{n}\mathbf{A11}'+\frac{1}{n^{2}}\mathbf{11'A11}')) \\ =tr(\mathbf{AA})-\frac{2}{n}\mathbf{1}'\mathbf{AA1}+\frac{1}{n^{2}}\mathbf{1'A11'A1} \end{matrix}$,

or for an easier interpretation:

$\begin{matrix} E_{x}(\mathbf{x}'\mathbf{Ax})=tr(\mathbf{AA})-\frac{2}{n}\mathbf{1}'\mathbf{AA1}+\frac{1}{n^{2}}\mathbf{1'A11'A1} \\ =tr(\mathbf{AA})-\frac{1}{n^{2}}\mathbf{1'A11'A1}-2\left(\frac{1}{n}\mathbf{1}'\mathbf{AA1}-\frac{1}{n^{2}}\mathbf{1'A11'A1}\right) \end{matrix}$,

because $tr(\mathbf{AA})-\frac{1}{n^{2}}\mathbf{1'A11'A1}$ was equal to the variance of the coefficients of the matrix $\mathbf{A}$multiplied by $n^{2}$and $\left(\frac{1}{n}\mathbf{1}'\mathbf{AA1}-\frac{1}{n^{2}}\mathbf{1'A11'A1}\right)$was the variance of the sum of columns of $\mathbf{A}$.

## Calculation of $E_{x}(\mathbf{x}'\mathbf{C}_{\mathbf{uu}}^{(2)}\mathbf{x})$

$\begin{matrix} E_{x}(\mathbf{x}'\mathbf{C}_{\mathbf{uu}}^{(2)}\mathbf{x})=tr(\mathbf{C}_{\mathbf{uu}}^{(2)}V(\mathbf{x}))= \\ tr(\mathbf{C}_{\mathbf{uu}}^{(2)}(\mathbf{A}-\frac{1}{n}\mathbf{11}'\mathbf{A}-\frac{1}{n}\mathbf{A11}'+\frac{1}{n^{2}}\mathbf{11'A11}')) \\ =tr(\mathbf{C}_{\mathbf{uu}}^{(2)}\mathbf{A})-\frac{1}{n}\mathbf{1}'\mathbf{AC}_{\mathbf{uu}}^{(2)}\mathbf{1}-\frac{1}{n}\mathbf{1}'\mathbf{C}_{\mathbf{uu}}^{(2)}\mathbf{A1}+\frac{1}{n^{2}}\mathbf{1'A11}'\mathbf{C}_{\mathbf{uu}}^{(2)}\mathbf{1} \\ =tr(\mathbf{A})-\lambda^{(2)}tr(\mathbf{C}_{\mathbf{uu}}^{(2)})-tr(\mathbf{C}_{\mathbf{u}1}^{(2)}\mathbf{1}'\mathbf{A})-\frac{1}{n}\mathbf{1}'\mathbf{AC}_{\mathbf{uu}}^{(2)}\mathbf{1}-\frac{1}{n}\mathbf{1}'\mathbf{C}_{\mathbf{uu}}^{(2)}\mathbf{A1}+\frac{1}{n^{2}}\mathbf{1'A11}'\mathbf{C}_{\mathbf{uu}}^{(2)}\mathbf{1} \\ =tr(\mathbf{A})-\lambda^{(2)}tr(\mathbf{C}_{\mathbf{uu}}^{(2)})+\frac{1}{n}tr(\mathbf{C}_{\mathbf{uu}}^{(2)}\mathbf{11}'\mathbf{A})-\frac{1}{n}\mathbf{1}'\mathbf{AC}_{\mathbf{uu}}^{(2)}\mathbf{1}-\frac{1}{n}\mathbf{1}'\mathbf{C}_{\mathbf{uu}}^{(2)}\mathbf{A1}+\frac{1}{n^{2}}\mathbf{1'A11}'\mathbf{C}_{\mathbf{uu}}^{(2)}\mathbf{1} \\ =tr(\mathbf{A})-\lambda^{(2)}tr(\mathbf{C}_{\mathbf{uu}}^{(2)})-\frac{1}{n}\mathbf{1}'\mathbf{C}_{\mathbf{uu}}^{(2)}\mathbf{A1}+\frac{1}{n^{2}}\mathbf{1'A11}'\mathbf{C}_{\mathbf{uu}}^{(2)}\mathbf{1} \\ =tr(\mathbf{A})-\lambda^{(2)}tr(\mathbf{C}_{\mathbf{uu}}^{(2)})-(\frac{1}{n}\mathbf{1}'\mathbf{C}_{\mathbf{uu}}^{(2)}\mathbf{A1}-\frac{1}{n^{2}}\mathbf{1'A11}'\mathbf{C}_{\mathbf{uu}}^{(2)}\mathbf{1}) \end{matrix}$,

using the permutation property of the trace in order to obtain a scalar.

## Calculation of $E_{x}(\mathbf{x}'\mathbf{C}_{\mathbf{uu}}^{(2)}\mathbf{C}_{\mathbf{uu}}^{(2)}\mathbf{x})$

$\begin{matrix} E_{x}(\mathbf{x}'\mathbf{C}_{\mathbf{uu}}^{(2)}\mathbf{C}_{\mathbf{uu}}^{(2)}\mathbf{x})=tr(\mathbf{C}_{\mathbf{uu}}^{(2)}\mathbf{C}_{\mathbf{uu}}^{(2)}V(\mathbf{x}))= \\ tr(\mathbf{C}_{\mathbf{uu}}^{(2)}\mathbf{C}_{\mathbf{uu}}^{(2)}(\mathbf{A}-\frac{1}{n}\mathbf{11}'\mathbf{A}-\frac{1}{n}\mathbf{A11}'+\frac{1}{n^{2}}\mathbf{11'A11}')) \\ =tr(\mathbf{C}_{\mathbf{uu}}^{(2)}\mathbf{C}_{\mathbf{uu}}^{(2)}\mathbf{A})-\frac{2}{n}\mathbf{1}'\mathbf{AC}_{\mathbf{uu}}^{(2)}\mathbf{C}_{\mathbf{uu}}^{(2)}\mathbf{1}+\frac{1}{n^{2}}\mathbf{1'A11}'\mathbf{C}_{\mathbf{uu}}^{(2)}\mathbf{C}_{\mathbf{uu}}^{(2)}\mathbf{1} \\ =tr(\mathbf{C}_{\mathbf{uu}}^{(2)}(\mathbf{A}-\lambda^{(2)}\mathbf{C}_{\mathbf{uu}}^{(2)}-\mathbf{C}_{\mathbf{u}1}^{(2)}\mathbf{1}'\mathbf{A}))-\frac{2}{n}\mathbf{1}'\mathbf{AC}_{\mathbf{uu}}^{(2)}\mathbf{C}_{\mathbf{uu}}^{(2)}\mathbf{1}+\frac{1}{n^{2}}\mathbf{1'A11}'\mathbf{C}_{\mathbf{uu}}^{(2)}\mathbf{C}_{\mathbf{uu}}^{(2)}\mathbf{1} \\ =tr(\mathbf{A})-\lambda^{(2)}tr(\mathbf{C}_{\mathbf{uu}}^{(2)})-tr(\mathbf{C}_{\mathbf{u}1}^{(2)}\mathbf{1}'\mathbf{A})-\lambda^{(2)}tr(\mathbf{C}_{\mathbf{uu}}^{(2)}\mathbf{C}_{\mathbf{uu}}^{(2)})-tr(\mathbf{C}_{\mathbf{uu}}^{(2)}\mathbf{C}_{\mathbf{u}1}^{(2)}\mathbf{1}'\mathbf{A})-\frac{2}{n}\mathbf{1}'\mathbf{AC}_{\mathbf{uu}}^{(2)}\mathbf{C}_{\mathbf{uu}}^{(2)}\mathbf{1}+\frac{1}{n^{2}}\mathbf{1'A11}'\mathbf{C}_{\mathbf{uu}}^{(2)}\mathbf{C}_{\mathbf{uu}}^{(2)}\mathbf{1} \\ =tr(\mathbf{A})-\lambda^{(2)}tr(\mathbf{C}_{\mathbf{uu}}^{(2)})-\lambda^{(2)}tr(\mathbf{C}_{\mathbf{uu}}^{(2)}\mathbf{C}_{\mathbf{uu}}^{(2)})-\mathbf{1}'\mathbf{AC}_{\mathbf{u}1}^{(2)}-\mathbf{1}'\mathbf{AC}_{\mathbf{uu}}^{(2)}\mathbf{C}_{\mathbf{u}1}^{(2)}-\frac{2}{n}\mathbf{1}'\mathbf{AC}_{\mathbf{uu}}^{(2)}\mathbf{C}_{\mathbf{uu}}^{(2)}\mathbf{1}+\frac{1}{n^{2}}\mathbf{1'A11}'\mathbf{C}_{\mathbf{uu}}^{(2)}\mathbf{C}_{\mathbf{uu}}^{(2)}\mathbf{1} \\ =tr(\mathbf{A})-\lambda^{(2)}tr(\mathbf{C}_{\mathbf{uu}}^{(2)})-\lambda^{(2)}tr(\mathbf{C}_{\mathbf{uu}}^{(2)}\mathbf{C}_{\mathbf{uu}}^{(2)})+\frac{1}{n}\mathbf{1}'\mathbf{AC}_{\mathbf{uu}}^{(2)}\mathbf{1}-\frac{1}{n}\mathbf{1}'\mathbf{AC}_{\mathbf{uu}}^{(2)}\mathbf{C}_{\mathbf{uu}}^{(2)}\mathbf{1}+\frac{1}{n^{2}}\mathbf{1'A11}'\mathbf{C}_{\mathbf{uu}}^{(2)}\mathbf{C}_{\mathbf{uu}}^{(2)}\mathbf{1} \end{matrix}$.

## Calculation of$E_{x}(\mathbf{x}'\mathbf{C}_{\mathbf{uu}}^{(2)}\mathbf{11}'\mathbf{C}_{\mathbf{uu}}^{(2)}\mathbf{x})$

$\begin{matrix} E_{x}(\mathbf{x}'\mathbf{C}_{\mathbf{uu}}^{(2)}\mathbf{11}'\mathbf{C}_{\mathbf{uu}}^{(2)}\mathbf{x})=tr(\mathbf{C}_{\mathbf{uu}}^{(2)}\mathbf{11}'\mathbf{C}_{\mathbf{uu}}^{(2)}V(\mathbf{x}))= \\ tr(\mathbf{C}_{\mathbf{uu}}^{(2)}\mathbf{11}'\mathbf{C}_{\mathbf{uu}}^{(2)}(\mathbf{A}-\frac{1}{n}\mathbf{11}'\mathbf{A}-\frac{1}{n}\mathbf{A11}'+\frac{1}{n^{2}}\mathbf{11'A11}')) \\ =tr(\mathbf{C}_{\mathbf{uu}}^{(2)}\mathbf{11}'\mathbf{C}_{\mathbf{uu}}^{(2)}\mathbf{A})-\frac{2}{n}\mathbf{1}'\mathbf{AC}_{\mathbf{uu}}^{(2)}\mathbf{11}'\mathbf{C}_{\mathbf{uu}}^{(2)}\mathbf{1}+\frac{1}{n^{2}}\mathbf{1'A11}'\mathbf{C}_{\mathbf{uu}}^{(2)}\mathbf{11}'\mathbf{C}_{\mathbf{uu}}^{(2)}\mathbf{1} \\ =tr(\mathbf{C}_{\mathbf{uu}}^{(2)}\mathbf{11}'(\mathbf{A}-\lambda^{(2)}\mathbf{C}_{\mathbf{uu}}^{(2)}-\mathbf{C}_{\mathbf{u}1}^{(2)}\mathbf{1}'\mathbf{A}))-\frac{2}{n}\mathbf{1}'\mathbf{AC}_{\mathbf{uu}}^{(2)}\mathbf{11}'\mathbf{C}_{\mathbf{uu}}^{(2)}\mathbf{1}+\frac{1}{n^{2}}\mathbf{1'A11}'\mathbf{C}_{\mathbf{uu}}^{(2)}\mathbf{11}'\mathbf{C}_{\mathbf{uu}}^{(2)}\mathbf{1} \\ =\mathbf{1}'\mathbf{AC}_{\mathbf{uu}}^{(2)}\mathbf{1}-\lambda^{(2)}\mathbf{1}'\mathbf{C}_{\mathbf{uu}}^{(2)}\mathbf{C}_{\mathbf{uu}}^{(2)}\mathbf{1}+\frac{1}{n}\mathbf{1}'\mathbf{AC}_{\mathbf{uu}}^{(2)}\mathbf{11}'\mathbf{C}_{\mathbf{uu}}^{(2)}\mathbf{1}-\frac{2}{n}\mathbf{1}'\mathbf{AC}_{\mathbf{uu}}^{(2)}\mathbf{11}'\mathbf{C}_{\mathbf{uu}}^{(2)}\mathbf{1}+\frac{1}{n^{2}}\mathbf{1'A11}'\mathbf{C}_{\mathbf{uu}}^{(2)}\mathbf{11}'\mathbf{C}_{\mathbf{uu}}^{(2)}\mathbf{1} \\ =\mathbf{1}'\mathbf{AC}_{\mathbf{uu}}^{(2)}\mathbf{1}-\lambda^{(2)}\mathbf{1}'\mathbf{C}_{\mathbf{uu}}^{(2)}\mathbf{C}_{\mathbf{uu}}^{(2)}\mathbf{1}-\frac{1}{n}\mathbf{1}'\mathbf{AC}_{\mathbf{uu}}^{(2)}\mathbf{11}'\mathbf{C}_{\mathbf{uu}}^{(2)}\mathbf{1}+\frac{1}{n^{2}}\mathbf{1'A11}'\mathbf{C}_{\mathbf{uu}}^{(2)}\mathbf{11}'\mathbf{C}_{\mathbf{uu}}^{(2)}\mathbf{1} \end{matrix}$.

## Calculation of$E_{x}(\mathbf{Q}'\mathbf{Q})$

With the notations:

$$a_{ii}=\frac{1}{4}a_{s_{i}s_{i}}+\frac{1}{4}a_{d_{i}d_{i}}+\frac{1}{2}a_{s_{i}d_{i}}+d_{ii}=1+f_{i}$$

where $a_{ij}$ is the relationship coefficient between individual *i* and *j*, $s_{i}$ the sire of the individual *i,* $d_{i}$the dam of individual *i,* $d_{ii}$the Mendelian sampling effect and $f_{i}$the inbreeding coefficient of *i*:

$d_{ii}=\frac{1}{2}-\frac{1}{4}(f_{s_{i}}+f_{d_{i}})$.

If we consider

$\mathbf{\zeta}=\frac{\mathbf{w}_{s}+\mathbf{w}_{d}}{2}$, so that $\mathbf{z}=\mathbf{\zeta}-\mathbf{1}\bar{w}$,

then

$\mathbf{x}-\mathbf{z}=\mathbf{w}-\mathbf{\zeta}$ and $\mathbf{z}-\mathbf{1}\bar{z}=\mathbf{\zeta}-\mathbf{1}\bar{\zeta}$which were replaced in each coefficient of $\mathbf{Q}$.

The following results were useful:

$$E(w_{i}w_{i})=a_{ii}$$

$$E(w_{i}w_{j})=a_{ij}$$

$$E(w_{i}\zeta_{i})=E(w_{i}\left(\frac{w_{s_{i}}+w_{d_{i}}}{2}\right))=\frac{1}{2}a_{is_{i}}+\frac{1}{2}a_{id_{i}}=\frac{1}{4}a_{s_{i}s_{i}}+\frac{1}{4}a_{d_{i}d_{i}}+\frac{1}{2}a_{s_{i}d_{i}}=a_{ii}-d_{ii}$$

$$E(w_{i}\zeta_{j})=a_{ij}$$

$$E(\zeta_{i}\zeta_{i})=a_{ii}-d_{ii}$$

$$E(\zeta_{i}\zeta_{j})=a_{ij}$$

with the diagonal matrix of Mendelian sampling coefficients $d_{ii}$

So, the coefficients of the matrix and were:

$$E(\mathbf{1}'(\mathbf{z}-\mathbf{1}\bar{z}))=E(\mathbf{1}'(\mathbf{\zeta}-\mathbf{1}\bar{\zeta}))=0$$

$$E(\mathbf{1}'(\mathbf{x}-\mathbf{z}))=E(\mathbf{1}'(\mathbf{w}-\mathbf{\zeta}))=0$$

.

## Calculation of $E_{x}(\mathbf{Q}'\mathbf{x})$

Using the previous results, expectations became:

$$E(\mathbf{1}'(\mathbf{w}-\bar{\mathbf{w}}))=0$$

.

## Calculation of $E_{x}(\mathbf{QAQ}')$

Using the previous results, expectations became:

,

and

.
