# Supplementary material for: Statistical distributions of test statistics used for quantitative trait association mapping in structured populations
Source: Genet Sel Evol. 2012 Nov 12;44(1):32. doi: 10.1186/1297-9686-44-32 (PMC3817592; doi:10.1186/1297-9686-44-32)
Supplement: Additional file 2 — Simulations. Details and standard errors of the simulations used to confirm the analytical results. [file 1297-9686-44-32-S2.docx]

# Additional file 2

# Simulations

# Simulation parameters

A certain number of simulations were performed in order to validate the algebraic formulae described in our paper. All four methods i.e. regression, QTDT, GRAMMAR and FASTA were tested. The present validation was restricted to the family structures and heritability values used in the “Comparison of methods” section of the paper. The population used for the simulations consisted of 600 genotyped individuals, offspring of 120, 20 and 10 sires that produced 5, 30 and 60 offspring, respectively. To do this, the genotypes for a SNP were simulated for sires and dams with minimum allele frequencies of 0.5, and the genotypes of the offspring were extrapolated from their parents' genotypes. Next, the polygenic values of the sires and offspring and the phenotypes of the offspring were computed with and without the effect of a corresponding QTL with an allele substitution effect of 0.20 (equivalent to a regression coefficient of 0.141 for phenotypic standard deviation or a QTL explaining 2% of the phenotypic variance). The robustness and power of each method were then evaluated using these two phenotypes (with or without a QTL) with a significance threshold of 5% (which is different from the 1% threshold used in the paper). The simulations were performed with heritability values ranging from 0 to 1 by 0.1 steps. 10 000 simulations were carried out for each scenario. In total, 1 320 000 simulations were performed.

To keep the figures easy to interpret, standard errors of type I error rate and power were not added, except in figure 7. They depend on the estimated frequency.

For the GRAMMAR and FASTA methods, the ASREML software [66] was used to estimate variance components. It should also be noted that the relationship matrix used for these two methods is derived from pedigree data and not genomic data.

Results are summarized in a table at the end of this additional file.

# Results

In this section, differences between theoretical and simulated values are given as a function of type 1 and 2 errors (robustness and power), which are both expressed in percentage. Theoretical values were computed using a R program named RobPower.

## Regression model

Figure 1 shows the simulation results (dashed lines) and theoretical results (solid lines) for the regression model. For robustness, the average absolute value of the difference between the curves was of 0.26 % ($\pm$ 0.25) and was maximal (1.09%) for families with 60 offspring and a heritability of 1. The difference was, on average, greater for larger family structures (0.46% for families with 60 offspring compared to 0.11% for families with 5 offspring). For power, the average absolute value of the difference between the curves was 0.37% ($\pm$ 0.17) and was maximal (0.76%) for families with 60 offspring and a heritability of 0.3. The difference was, on average, greater for family structures with 60 offspring per family (0.46%).

On the whole, the differences were small, hence validating the theoretical results for the regression model.


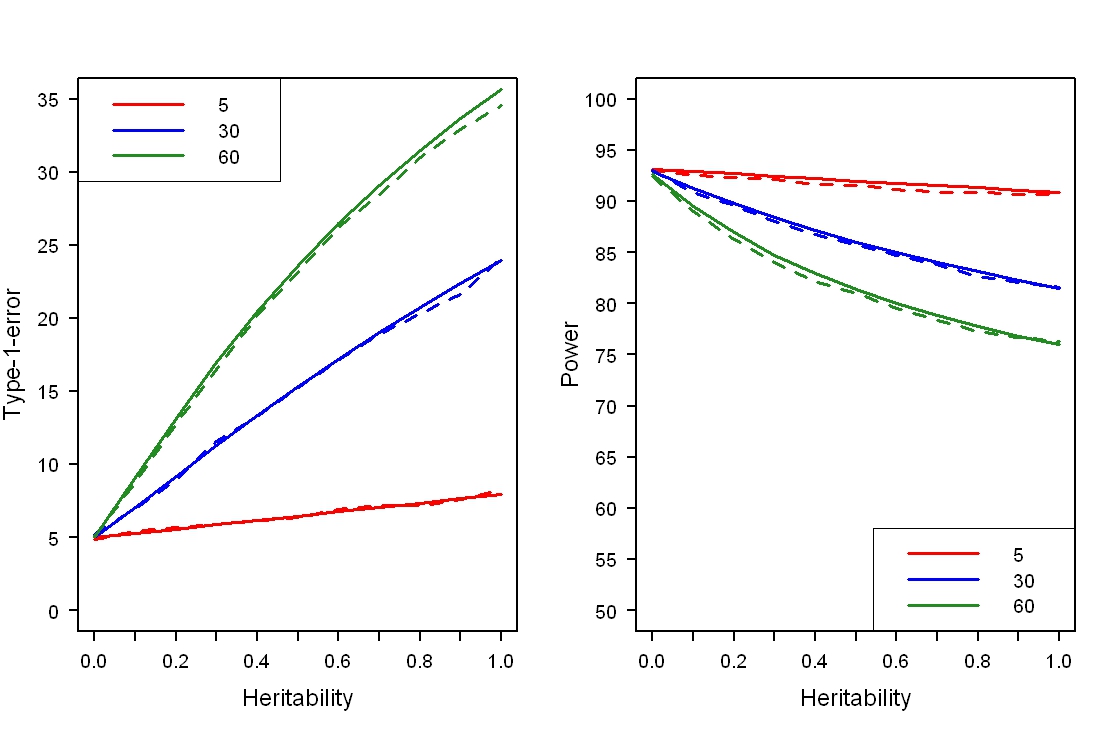


Figure 1- Robustness and power for the regression model

## qtdt model

Figure 2 shows the simulation results (dashed lines) and theoretical results (solid lines) for the QTDT model. For robustness, the average absolute value of the difference between the curves was 0.28% ($\pm$ 0.22) and was maximal (0.74%) for families with 60 offspring and a heritability of 0.7. The difference was, on average, greater for larger family structures (0.52% for families with 60 offspring compared to 0.11% for families with 5 offspring). For power, the average absolute value of the difference between the curves was 1.12% ($\pm$ 0.63) and was maximal (0.76%) for families with 60 offspring and a heritability of 0.3. The difference was, on average, greater for larger family structures (1.81% for families with 60 offspring compared to 0.47% for families with 5 offspring).

On the whole, the differences were relatively small, hence validating the theoretical results for the QTDT model.


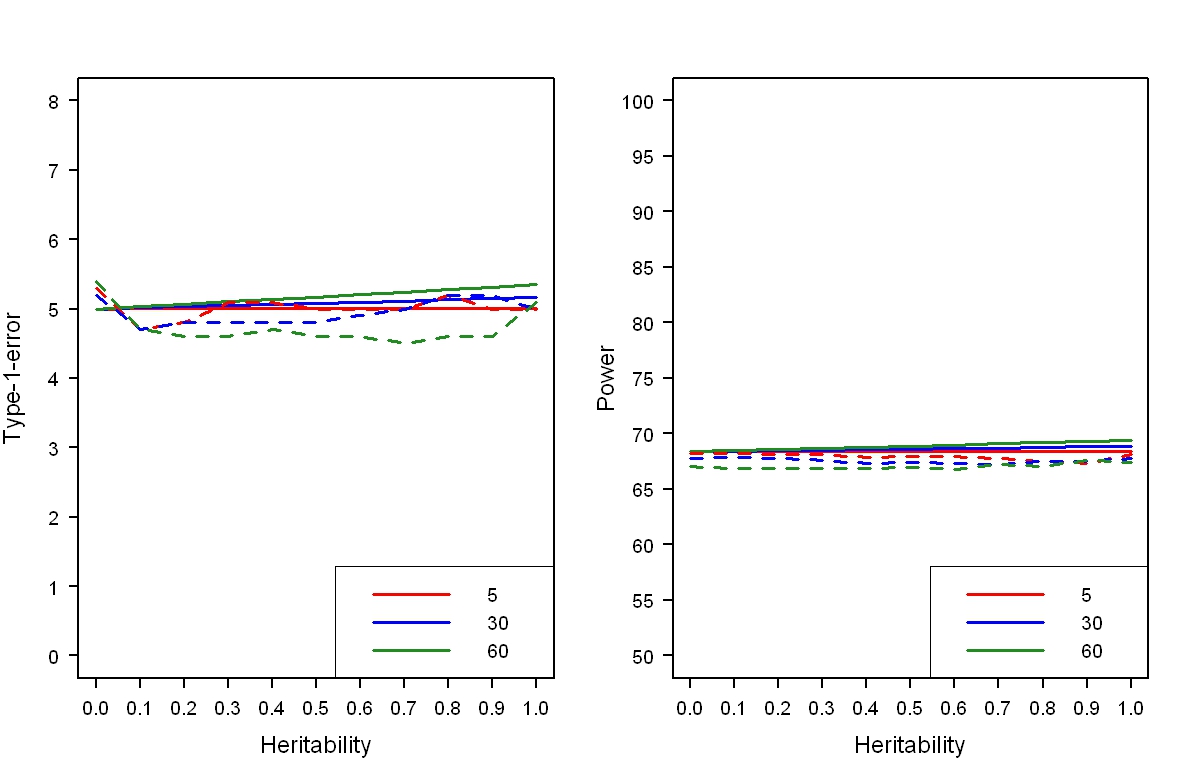


Figure 2 - Robustness and power for the QTDT model

## grammar model

Figure 3 shows the simulation results (dashed lines) and theoretical results (solid lines) for the grammar method. For robustness, the average absolute value of the difference between the curves was 0.22 % ($\pm$ 0.17) and was maximal (0.7%) for families with 60 offspring and a heritability value of 0. For power, the average absolute value of the difference between the curves was 0.58% ($\pm$ 0.71) and was maximal (2.97%) for families with 60 offspring and a heritability of 0.9. The difference was, on average, greater for larger family structures (1.16% for families with 60 offspring compared to 0.11% for families with 5 offspring). It should be noted that differences seem to increase with increasing heritability. A possible explanation is that the estimated heritability obtained with ASREML was biased (underestimated) for higher simulatedheritabilities. Figure 4 shows the distribution between expected (theoretical) and observed (simulated) heritabilities.


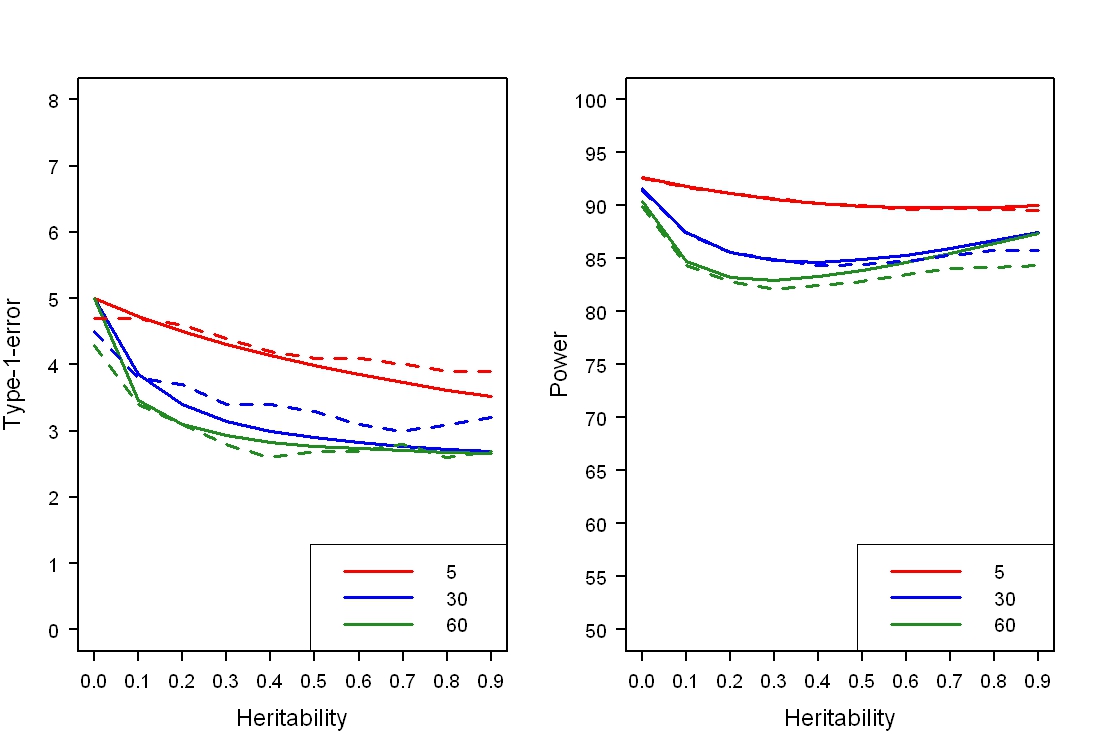


Figure 3 - Robustness and power for the grammar model


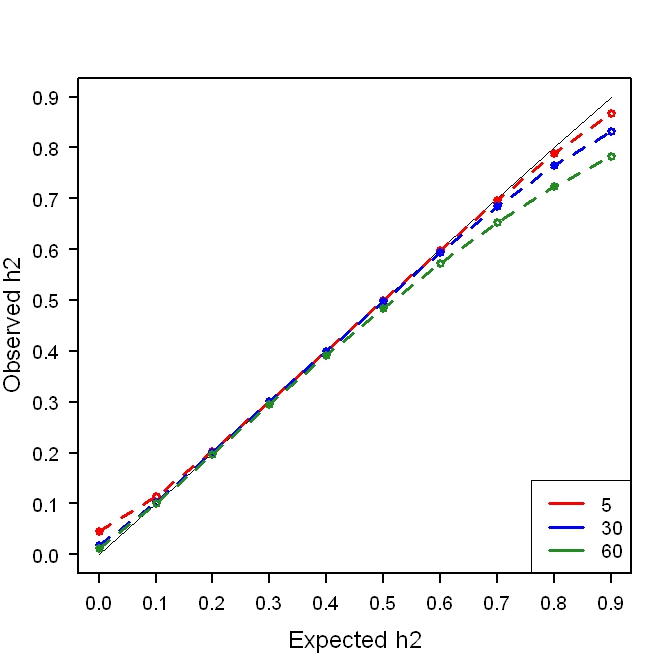


Figure 4 - Bias between expected and observed heritabilities for the grammar method

## fasta model

Figure 5 shows the simulation results (dashed lines) and theoretical results (solid lines) for the fasta model. For robustness, the average absolute value of the difference between the curves was 0.18% ($\pm$ 0.14) and was maximal (0.5%) for families with 30 offspring and a heritability of 0.6. For power, the average absolute value of the difference between the curves was 0.90% ($\pm$ 0.32) and was maximal (1.74%) for families with 60 offspring and a heritability of 1. The difference was, on average, greater for larger family structures (1.25% for families with 60 offspring compared to 0.59% for families with 5 offspring). As with the grammar method, it should be noted that differences seem to increase with increasing heritability and are potentially caused by a bias between the expected and observed heritabilities. Figure 6 shows the distribution between heritabilities.


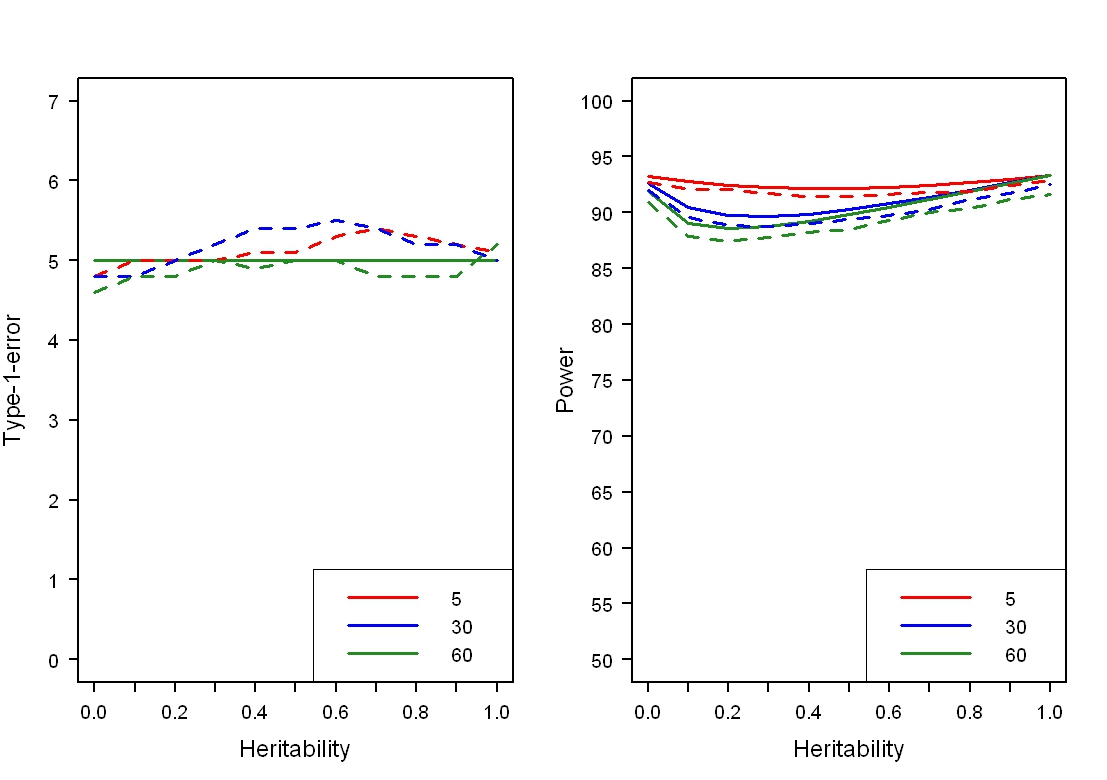


Figure 5 - Robustness and power for the fasta model


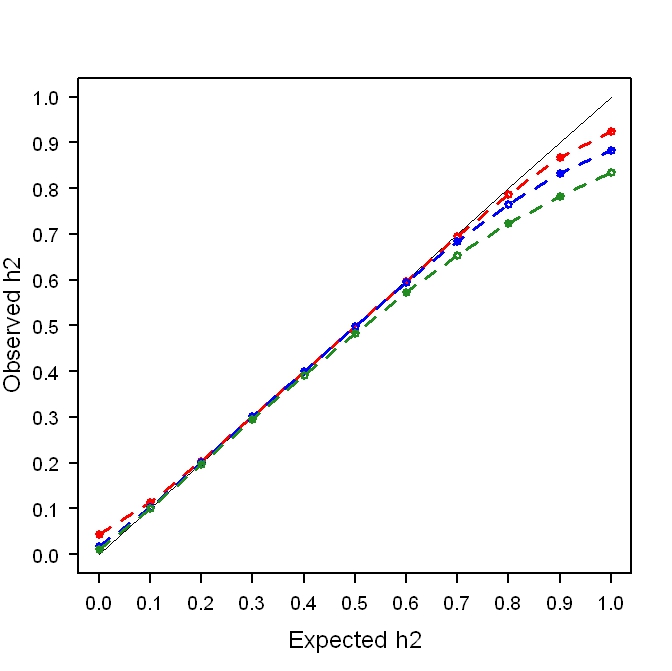


Figure 6 - Bias between expected and observed heritabilities for the FASTA method

## Standard error of estimates

Standard errors of the estimates are presented in figure 7 and depended on the estimated frequency. They were compared to the average deviation obtained with the four methods and are summarized in table 1. The average deviation of the simulations from the theoretical formulae were in general of the same order than the standard deviation (0.22% for 5% estimated) for type I error rate and slightly higher for power (0.36% for for 85% estimated). This may be due to artifacts with extremely high values of heritability.

Figure 7 - Standard error or type I error rate and power estimated by simulations (10 000 samples)

Table 1 – Average and maximum differences of type I error rate and power between simulated and theoretical results (%)

|  | Regression | grammar | Fasta | qtdt |
| --- | --- | --- | --- | --- |
| **Type I error rate** |  |  |  |  |
| Average difference | 0.26 | 0.22 | 0.18 | 0.28 |
| Maximum difference | 1.09 | 0.70 | 0.50 | 0.74 |
| **Power** |  |  |  |  |
| Average difference | 0.37 | 0.58 | 0.90 | 1.12 |
| Maximum difference | 0.76 | 2.97 | 1.74 | 2.19 |
