# Supplementary material for: Statistical distributions of test statistics used for quantitative trait association mapping in structured populations
Source: Genet Sel Evol. 2012 Nov 12;44(1):32. doi: 10.1186/1297-9686-44-32 (PMC3817592; doi:10.1186/1297-9686-44-32)
Supplement: Additional file 3 — RobPower. This is the directory including Rpackage and documentation. This package computes all type 1 errors and the power of any kind of design with: 1. The A matrix obtained either from the pedigree file or from a known matrix. 2. The D matrix obtained either from the pedigree file or from a known matrix. 3. A value of heritability (can be a single value or a vector). 4. A threshold (for example the nominal 5% threshold, 0.05). 5. The phenotypic variance explained by the QTL (can be a single value or a vector). 6. The methods to compute (can be a single value or a vector of characters). Methods are “reg” for regression model, “GRAMMAR”, “QTDT” and “FASTA”. 7. Genomic control (GC). If GC = TRUE then the GC value is computed (available for “reg” and “GRAMMAR” only). 8. Linkage disequilibrium (r2) between the marker tested and the QTL. [file 1297-9686-44-32-S3.zip › Documentation.pdf]

Contents

1 R function RobPower 2
1.1 Install RobPower 2
1.2 How to use RobPower ? 2
1.3 RobPower options 3
1.4 Examples 4
1.4.1 Example 1 : 4
1.4.2 Example 2 : 5

1 R function RobPower

RobPower is a R Program to compute algebric formulaes in references to the article "Influence of population structure on power and robustness of current association mapping tests" by S. Teyssèdre, J-M Elsen and A. Ricard.

1.1 Install RobPower

RobPower is a R Program. First, install R at http://www.r-project.org/. Then, download the file RobPower.rar at http://lien-vers-la-fonction and unzip it to a local directory on your computer.

1.2 How to use RobPower ?

1. Launch R
2. Change the Working Directory to the local directory where RobPower.rar was unzipped
3. Open the file Main.R
4. Execute the command

   ```
   source(file="fonctions/RobPower.r")
   ```

5. Make sure that your pedigree file is in format "ind sire dam is.perf" with no header and only numeric values. Ind, sire and dam are numeric values corresponding to an individual code and is.perf is a boolean (with value of 0 or 1) which determine if the individual "ind" is recorded or not. For example :

Table 1: Example of a pedigree file with 5 individuals where only 3 of them have their phenotypes been recorded

Ind	sire	dam	is.perf
1	0	0	0
2	0	0	0
3	1	2	1
4	1	0	1
5	3	4	1

6. Create a relationship matrix A from the pedigree. Two solutions :

- The A matrix is already available : If you already have the A matrix from all individuals of the pedigree then use the function recupA.R to get A and another matrix D (which is needed next) from only recorded individuals by executing the command :

  ```
  source(file="fonctions/recupA.r")
  Afull = read.table(file="name_of_A_matrix_file",header=FALSE)
  creation = recupA(fileped="name_of_pedigree_file",Afull)
  A = creation[[1]]
  D = creation[[2]]
  ```

- The A matrix is not avaible : If you want to compute the A matrix, you can use the R function creeA.R by executing the command :

  ```
  source(file="fonctions/creeA.r")
  creation = creeA(fileped="name_of_pedigree_file")
  A = creation[[1]]
  D = creation[[2]]
  ```

  but this step can be very long for large pedigree (>2000 individuals). It's better to use the program fortran 90 creeA.f90 to compute the A matrix and then to follow the instructions of the previous item.

7. You can now use RobPower

1.3 RobPower options

RobPower require the following arguments :

1. The A matrix (obtained previously)
2. The D matrix (obtained previously)
3. A value of heritability (can be a single value or a vector)
4. A threshold (for example the nominal 5% threshold, 0.05)
5. The phenotypic variance explained by the QTL (can be a single value or a vector)
6. The methods to compute (can be a single value or a vector of characters). Methods are "reg" for regression model, "grammar", "qtdt" and "fasta".
7. Genomic Control (GC). If GC=TRUE then the GC value is computing (available for "reg" and "grammar" only).
8. Linkage disequilibrium (r^2) between tested marker and QTL.

1.4 Examples

Here two examples which correspond to two pedigrees : one pedigree of 600 recorded progeny which come from a half-sib design with 10 sires with 60 progenies by sire (Example 1); and one pedigree which come from real data (Example 2).

1.4.1 Example 1 :

1. Create A matrix with R program creeA.r :

   ```
   creation = creeA(fileped="ped-article")
   A = creation[[1]]
   D = creation[[2]]
   ```

2. Compute RobPower for h2 in (0.1,0.2), a QTL that explained 0.1 and 0.5 of phenotypic variance and used all methods. We suppose here that the marker is the QTL so that the value of linkage disequilibrium between marker and QTL is 1.

   ```
   RobPower(A,D,h2=c(0.1,0.2),threshold=0.05,var.QTL=c(0.01,0.05),
   methods=c("reg","grammar","qtdt","fasta"),GC=FALSE, r2=1)
   ```

Results are :

```
$regression
$regression$Robustness
      h2 robustness
[1,] 0.1 0.09084824
[2,] 0.2 0.13153912
$regression$power
         0.01      0.05
0.1 0.6545135 0.9985542
0.2 0.6395986 0.9961300


$qtdt
$qtdt$Robustness
      h2 robustness
[1,] 0.1 0.05041958
[2,] 0.2 0.05084288
$qtdt$power
         0.01      0.05
0.1 0.4101220 0.9719398
```

```
0.2 0.4115157 0.9721697


$grammar
$grammar$Robustness
      h2 robustness
[1,] 0.1 0.03462133
[2,] 0.2 0.03093204
$grammar$power
         0.01      0.05
0.1 0.5538103 0.9980230
0.2 0.5303303 0.9976164


$fasta
$fasta$Robustness
      h2 robustness
[1,] 0.1       0.05
[2,] 0.2       0.05
$fasta$power
         0.01      0.05
0.1 0.6162696 0.9989204
0.2 0.6095338 0.9988134
```

1.4.2 Example 2 :

You already have a A matrix

1. Load A with :

   ```
   Afull = read.table(file="A.txt",header=FALSE)
   ```

2. Use recupA.r with :

   ```
   reation = recupA(fileped="ped-real",Afull)
   A = creation[[1]]
   D = creation[[2]]
   ```

3. Compute RobPower from h2 between 0.1 and 0.5, a QTL effect of 0.02 and reg and fasta. We suppose here that the tested marker is in linkage disequilibrium with the QTL with a value of 0.4.

   ```
   RobPower(A,D,h2=seq(0.1,0.5,0.1),threshold=0.05,var.QTL=0.02,
   methods=c("reg","fasta"),GC=TRUE,r2=0.4)
   ```

Results are :

```
$$regression
$regression$Robustness
      h2 robustness
[1,] 0.1 0.05525119
[2,] 0.2 0.06058368
[3,] 0.3 0.06597818
[4,] 0.4 0.07141766
[5,] 0.5 0.07688714

$regression$power
         0.02
0.1 0.5332676
0.2 0.5326952
0.3 0.5321597
0.4 0.5316574
0.5 0.5311852

$regression$GC
      h2       GC
[1,] 0.1 1.045440
[2,] 0.2 1.090910
[3,] 0.3 1.136410
[4,] 0.4 1.181939
[5,] 0.5 1.227498


$fasta
$fasta$Robustness
      h2 robustness
[1,] 0.1       0.05
[2,] 0.2       0.05
[3,] 0.3       0.05
[4,] 0.4       0.05
[5,] 0.5       0.05

$fasta$power
         0.02
0.1 0.5193962
0.2 0.5115203
0.3 0.5067154
0.4 0.5041759
```

```
0.5 0.5035639
```
